# Domino C–C/C–O bond formation: palladium-catalyzed regioselective synthesis of 7-iodobenzo[*b*]furans using 1,2,3-triiodobenzenes and benzylketones†

**DOI:** 10.1039/d1ra05730h

**Published:** 2021-09-08

**Authors:** Raed M. Al-Zoubi, Walid K. Al-Jammal, Michael J. Ferguson, Graham K. Murphy

**Affiliations:** Department of Chemistry, Jordan University of Science and Technology P.O. Box 3030 Irbid 22110 Jordan rmzoubi@just.edu.jo +962-2-7201071 +962-2-7201000 ext. 23651; Department of Chemistry, Gunning-Lemieux Chemistry Centre, University of Alberta Edmonton Alberta T6G2G2 Canada; Department of Chemistry, University of Waterloo Waterloo Ontario N2L3G1 Canada graham.murphy@uwaterloo.ca

## Abstract

A facile and efficient synthesis of 7-iodobenzo[*b*]furan derivatives *via* a highly regioselective tandem α-arylation/intramolecular *O*-arylation of 5-substituted-1,2,3-triiodobenzenes and benzylketones is described. Remarkably, the α-arylation coupling reactions initiate exclusively at the least sterically-hindered position of the triiodoarene, which results in a highly chemoselective transformation. The highest yields were observed in reactions between electron-poor 1,2,3-triiodoarenes and electron-rich benzylketones, yet the optimized reaction conditions were found to be tolerant to a wide range of different functional groups. This unprecedent synthesis of 7-iodobenzo[*b*]furans from 1,2,3-triiodobenzenes is scalable, general in scope, and provides easy access to valuable precursors for other chemical transformations.

## Abstract 2

A facile and unprecedented synthesis of 7-iodobenzo[*b*]furans *via* a highly regioselective tandem α-arylation/intramolecular *O*-arylation is reported that is efficient, scalable and creates versatile precursors for further chemical manipulation.

## Graphical abstract

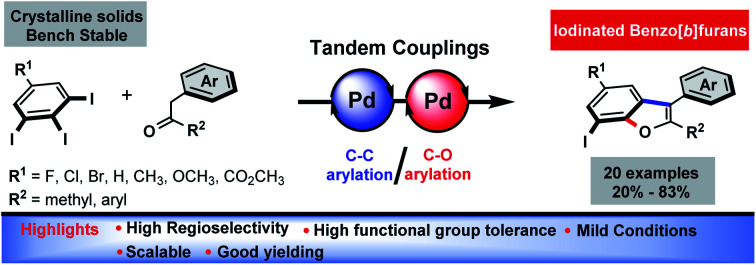

## Introduction

**Fig. 1:**
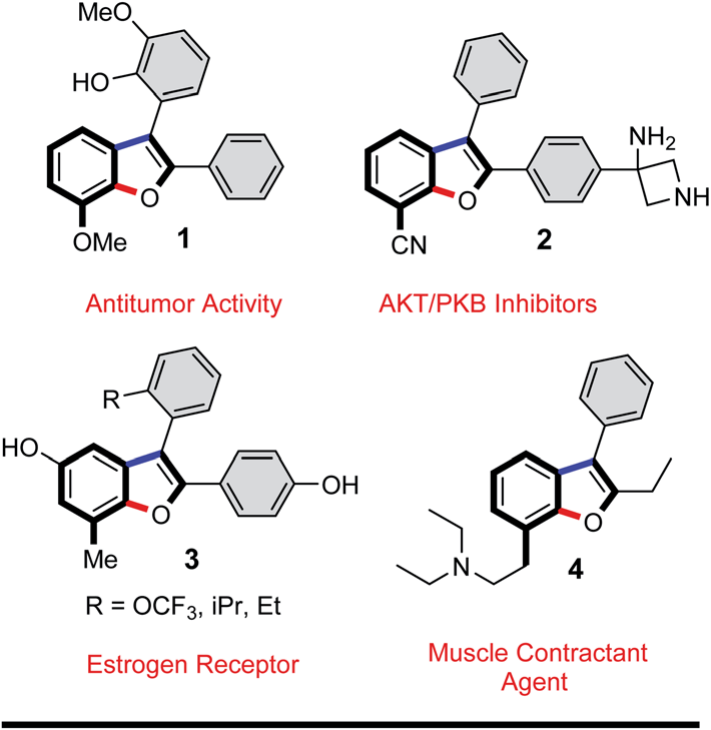
Some biologically active 2,3,7-trisubstituted benzo[*b*]furans in medicine.

Efficient annulation protocols that quickly access highly functionalized and valuable intermediates by means of site-selective functionalization of simple precursors is a powerful tool in synthetic chemistry and biology. In addition to the bond forming efficiency and atom-economy of these domino protocols, the potential for discovering new routes to access important functionalization patterns on privileged scaffolds is of great interest in organic materials and medicinal chemistry. Indeed, benzo[*b*]furans are ubiquitous structural motifs found as core components in organic materials such as organic transistors^1^ or organic solar cells,^2^ and in natural products such as anigopreissin A and amurensins L,^3^ and in pharmaceuticals such as 6-APB® (informally called benzo-Fury),^4^ antitumor agents,^5^ antimicrobials,^6^ 5-lipoxygenase inhibitors,^7^ and angiotensin II inhibitors.^8^ Bioactive examples of the specific 7-substituted 2,3-diarylbenzo[*b*]furan motif include 7-methoxy-2,3-diaryl-benzo[*b*]furan derivative 1 (Fig. 1), which is reported to inhibit proliferation of HeLa cells by apoptosis induction.^9^ The 7-cyano-2,3-diarylbenzofuran derivative 2 has inhibitory activity towards serine threonine kinase AKT for treating cancer,^10^ whereas the 7-methyl-2,3-diarylbenzofuran derivative 3 exhibits binding affinity to α- or β-subtype estrogen receptors.^11^ Lastly, the 7-aminoethyl-2,3-diarylbenzofuran derivative 4 is described as a muscle contractant with good urethral action for treatment of urinary incontinence.^12^ Among the numerous synthetic protocols that have been developed to make substituted benzo[*b*]furans,^6*b*,13^ the 2,3-diarylbenzo[*b*]furan scaffold has received considerable attention.^14^ Few of these methods employed tandem α-arylation/intramolecular *O*-arylations between haloarenes and ketones, even though transition metal-catalyzed domino reactions are efficient and possibly ideal methodologies.^13*i*,*k*,15^ In one example, Arisawa *et al.* reported a regioselective rhodium-catalyzed tandem C–C/C–O arylation between *ortho*-difluorobenzenes and ketones. In this case, the C–C coupling reaction took place at the least sterically hindered (and most electron deficient) C–F bond giving, as the state of the art, 6-substituted 2,3-diarylbenzo[*b*]furans in good yields.^15*a*^ In another example, Willis *et al.* reported a two-step process for the synthesis of benzo[*b*]furan derivatives *via* two independent palladium catalyzed C–C/C–O arylation reactions between polyhaloarenes and ketones. In this instance, the first step involved an excellent chemo- and regioselective C–C coupling at the iodo substituent, even in the presence of bromine and/or fluorine substituents, giving the products in good yields.^15*d*,*e*^ Given the importance of 2,3-diarylbenzo[*b*]furan derivatives in literature,^16^ and building on our interest in transformations of 5-substituted-1,2,3-triiodobenzenes,^17^ we too were encouraged to develop a new protocol to quickly access this motif. Our goal was to generate densely functionalized benzo[*b*]furan motifs, all with iodine at C-7 to serve as a precursor for further synthetic manipulation. Herein, we report our efforts to develop the first domino C–C/C–O arylation reaction between 1,2,3-triiodobenzenes and benzylketones to make 7-iodo-2,3-diarylbenzo[*b*]furan derivatives.

**Scheme 1:**
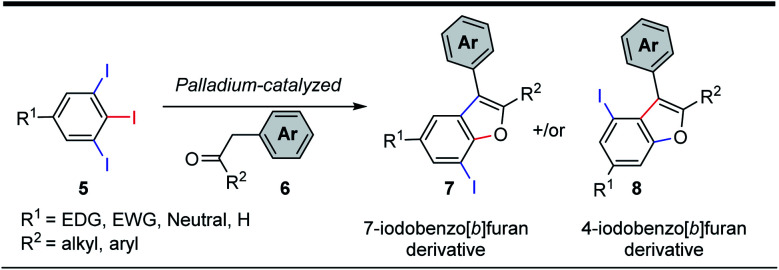
Possible iodinated benzofuran regioisomers from C–C/C–O arylations of 5-substituted-1,2,3-triiodobenzenes.

The 5-substituted-1,2,3-triiodobenzene starting materials (5) were synthesized from anilines or benzoic acid derivatives.^18^ Their C–I bonds are regiochemically differentiated (Scheme 1, red *vs.* blue), and as such, two regioisomeric C–C/C–O arylation products are possible: the 7-iodo-2,3-disubstituted benzo[*b*]furan (7) and the 4-iodo-2,3-disubstituted benzo[*b*]furan (8). It was expected using an equimolar loading of ketone 6 would be sufficient for achieving the tandem C–C/C–O arylations across two of the aryliodide groups, and that the configuration of the benzo[*b*]furan products would depend solely on the site of initial C–I bond activation.

To explore this hypothesis, 1,2,3-triiodobenzene 5a and 1,2-bis(4-methoxyphenyl)ethan-1-one (6a) were used as model substrates for reaction optimization, which is summarized in Table 1. The initial reaction of 5a with 1.0 equiv. of 6a under Miura conditions^15*f*^ (0.1 equiv. Pd(OAc)_2_, 0.2 equiv. PPh_3_ in anhydrous DMF at 120 °C for 12 h) provided 18% yield of benzo[*b*]furan product 7a (Table 1, entry 1). The reaction was highly regioselective for initiation at the least sterically hindered of C–I bonds and gave the 7-iodobenzo[*b*]furan product 7a as the sole isomer, with none of regioisomer 8a being observed. Changing the solvent to toluene improved the yield of the reaction to 27% (entry 2), whereas increasing the loading of both the ligand and catalyst only slightly increased the yields, to 29% and 32%, respectively (entries 3 and 4). The optimal reaction concentration was found to be 0.1 M (entries 5 and 6), and the addition of basic additives (*e.g.* Cs_2_CO_3_) proved detrimental (entries 7 and 8). Changing to the more reactive catalyst tetrakis(triphenylphosphine)palladium(0) provided 37% yield of benzo[*b*]furan product 7a (entry 9), and under these conditions, the addition of Cs_2_CO_3_ proved beneficial, providing 7a in 64% yield (entries 10–12). Raising the reaction temperature to 130 °C increased the yield to 75% (entry 13), and any further attempts to improve the reaction (solvent, base, reaction apparatus) were found to be unproductive (entries 14–17). A final reaction using the optimized conditions (entry 13) showed the tandem α-arylation/intramolecular *O*-arylation reaction also performs on gram scale, giving 7a in 67% yield (entry 18).

**Table 1:** Condition for one pot domino C–C/C–O arylations reaction 1,2,3-triiodobenzene 5 with 1,2-bis(4-methoxy phenyl)ethane-1-one^a^

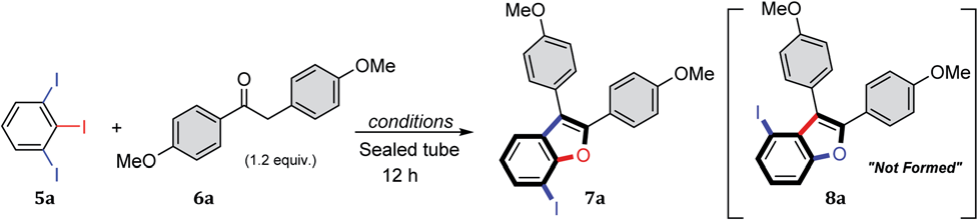						
Entry	Catalyst (mol%)	Ligand (mol%)	Base (equiv.)	Solvent [M]	*T* (°C)	Yield of 7a^b^ (%)
1	Pd(OAc)_2_ (**10**)	PPh_3_ (**20**)	NA	DMF, [**0.2**]	120	18%
2	Pd(OAc)_2_ (**10**)	PPh_3_ (**20**)	NA	Toluene, [**0.2**]	120	27%
3	Pd(OAc)_2_ (**10**)	PPh_3_ (**30**)	NA	Toluene, [**0.2**]	120	29%
4	Pd(OAc)_2_ (**20**)	PPh_3_ (**30**)	NA	Toluene, [**0.2**]	120	32%
5	Pd(OAc)_2_ (**20**)	PPh_3_ (**30**)	NA	Toluene, [**0.15**]	120	34%
6	Pd(OAc)_2_ (**20**)	PPh_3_ (**30**)	NA	Toluene, [**0.1**]	120	39%
7	Pd(OAc)_2_ (**20**)	PPh_3_ (**30**)	Cs_2_CO_3_ (**2**)	Toluene, [**0.1**]	120	37%
8	Pd(OAc)_2_ (**20**)	PPh_3_ (**30**)	Cs_2_CO_3_ (**3**)	Toluene, [**0.1**]	120	35%
9	Pd(PPh_3_)_4_ (**20**)	NA	NA	Toluene, [**0.1**]	120	37%
10	Pd(PPh_3_)_4_ (**20**)	NA	Cs_2_CO_3_ (**2**)	Toluene, [**0.1**]	120	54%
11	Pd(PPh_3_)_4_ (**20**)	NA	Cs_2_CO_3_ (**3**)	Toluene, [**0.1**]	120	64%
12	Pd(PPh_3_)_4_ (**20**)	NA	Cs_2_CO_3_ (**4**)	Toluene, [**0.1**]	120	65%
**13**	**Pd(PPh**_**3**_ **)**_**4**_ **(20)**	**NA**	**Cs**_**2**_ **CO**_**3**_ **(3)**	**Toluene, [0.1]**	**130**	**75%**
14	Pd(PPh_3_)_4_ (**20**)	NA	Cs_2_CO_3_ (**3**)	Toluene, [**0.2**]	130	61%
15	Pd(PPh_3_)_4_ (**20**)	NA	K_2_CO_3_ (**3**)	Toluene, [**0.1**]	130	59%
16	Pd(PPh_3_)_4_ (**20**)	NA	Cs_2_CO_3_ (**3**)	*O*-Xylene, [**0.1**]	130	51%
17^c^	Pd(PPh_3_)_4_ (**20**)	NA	Cs_2_CO_3_ (**3**)	Toluene, [**0.1**]	130	29%
18^d^	Pd(PPh_3_)_4_ (**20**)	NA	Cs_2_CO_3_ (**3**)	Toluene, [**0.1**]	130	67%

a Conditions: All reactions were carried out using 0.65 mmol (1.0 equiv., 0.1 M) of 1,2,3-triiodobenzene 5 in 6.5 solvent.

b Isolated yields.

c Reflux was used.

d 1.0 gram scale (2.19 mmol).

**Scheme 2:**
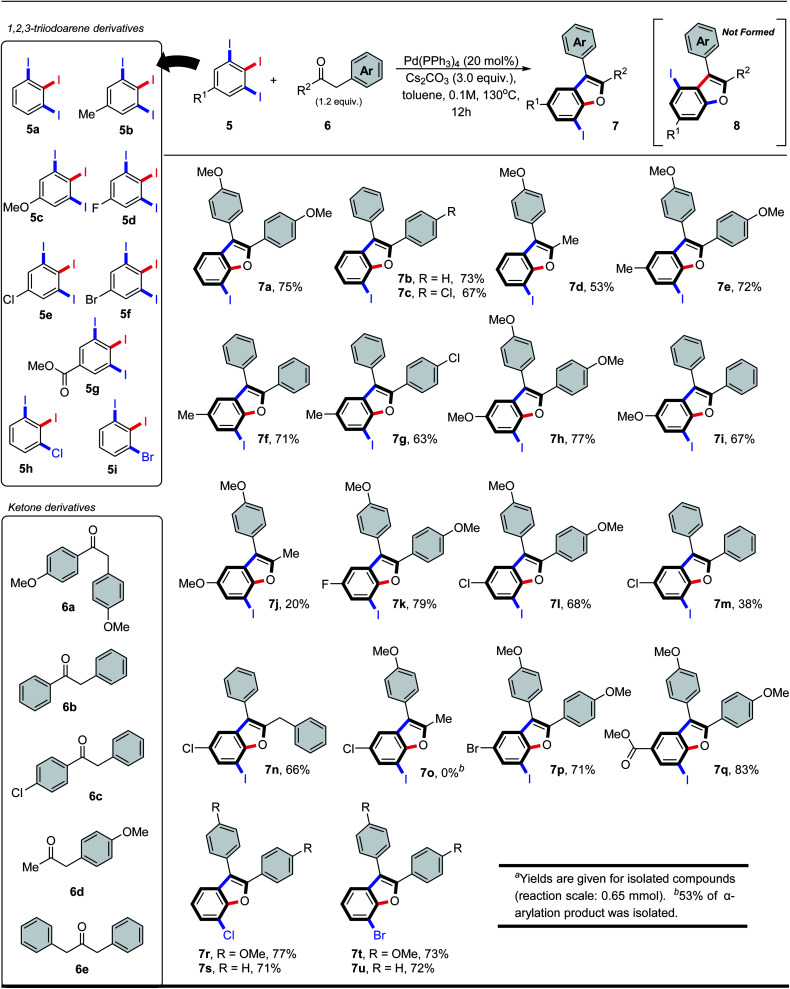
7-Iodinated benzofurans *via* regioselective tandem C–C/C–O arylations of 1,2,3-triiodobenzene and benzylketone derivative.^*a*^

Having identified optimal reaction conditions, we then investigated the scope of the α-arylation/intramolecular *O*-arylation reaction. 5-Substituted-1,2,3-triiodo-benzenes (5) were reacted with a series of acetophenone (6a–6c) and phenylacetone (6d, 6e) derivatives, and in each case they gave benzo[*b*]furan products 7 as the sole regioisomer (see Scheme 2). While the nature of the substituent (5, R^1^) was found to impact the reactivity observed in the domino process, it had no impact on the site-selectivity of the initial C–C bond forming step. For instance, 1,2,3-triiodoarenes bearing electron-poor/neutral substituents (5a, 5d, 5e, 5g) afforded higher isolated yields of the benzo[*b*]furan products (Scheme 2, 7a–7c, 7k, 7l and 7q), whereas substrates bearing electron-rich substituents (*e.g.*5c) afforded moderate isolated yields of products 7h–7j. Switching from acetophenone to phenylacetone derivatives (*e.g.*6d, 6e) was feasible, although this resulted in a general decrease in isolated yields of products 7d, 7j, 7n and 7o. Acetophenone derivatives possessing electron-rich substituents were found equivalent, if not slightly better than neutral derivatives, providing equivalent or higher isolated yields of the products (*e.g.*7a*vs.*7b, 7e*vs.*7f, 7l*vs.*7m). The highest isolated yield of the α-arylation/intramolecular *O*-arylation product was observed from electron-poor 1,2,3-triiodoarene (5d, 5g) reacting with electron-rich acetophenone 6a, giving products 7k and 7q. We also tested the reaction compatibility when one of the iodides was replaced with either bromide or chloride. The aryl chloride 5h reacted with acetophenone derivatives 6a and 6b to give products 7r and 7s in 77 and 71% yield, respectively. A similar test between aryl bromide 5i and acetophenones 6a and 6b also gave products 7t and 7u in 73 and 72% yield, respectively. In all these trials, no coupling was observed with either the bromo or chloro substituents, and the yields remained consistent with the analogous iodine-containing derivatives 7a or 7b.

**Fig. 2:**
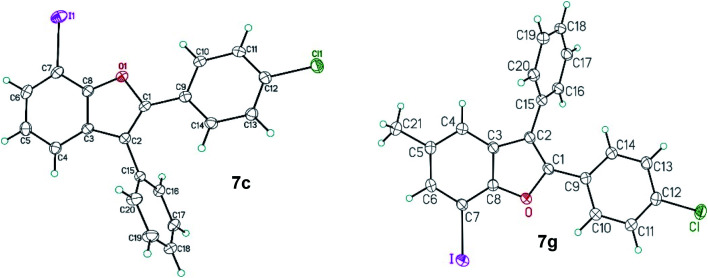
ORTEP view of 2-(4-chlorophenyl)-7-iodo-3-phenyl-1-benzo[*b*]furan 7c and 2-(4-chlorophenyl)-7-iodo-5-methyl-3-phenyl-1-benzo[*b*]furan 7g. Thermal Gaussian ellipsoids at 30% probability level.

The regiochemical outcome for the 7-iodo-2,3-diarylbenzo[*b*]furan products was confirmed using X-ray diffraction methods for two C–C/C–O arylation products, 2-(4-chlorophenyl)-7-iodo-3-phenyl-1-benzo[*b*]furan 7c and 2-(4-chlorophenyl)-7-iodo-5-methyl-3-phenyl-1-benzo[*b*]furan 7g (Fig. 2).^19^

**Scheme 3:**
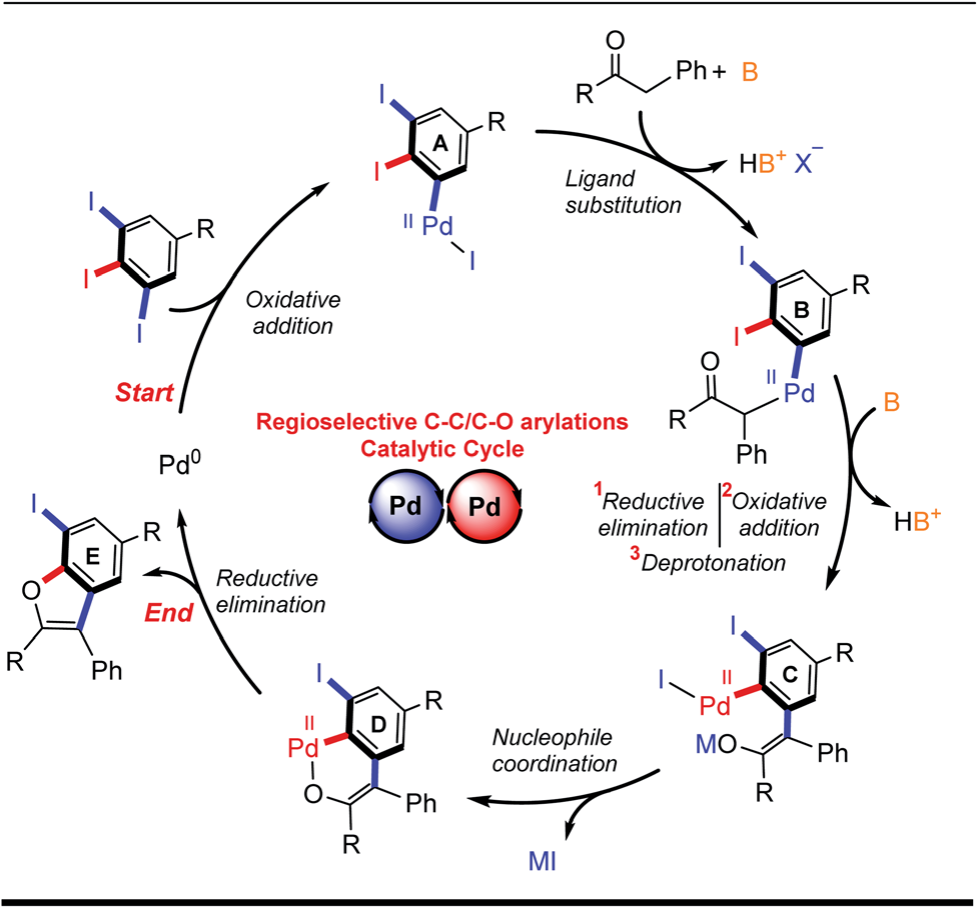
Proposed catalytic cycle for regioselective domino α-arylation/intramolecular *O*-arylation reaction of 5-substituted-1,2,3-triiodobenzene.

Based on our experimental results and on previously-reported mechanistic studies,^15*d*–*f*,20^ a reasonable catalytic cycle for the palladium-catalyzed domino C–C/C–O arylation reaction of 5-substituted-1,2,3-triiodobenzenes is proposed (Scheme 3). It begins with oxidative addition at the least sterically hindered C–I site of 5, giving Pd^II^ adduct A. Enolization of ketone 6 is followed by coordination with A and ligand exchange, giving Pd^II^-intermediate B. Sequential reductive elimination, directed oxidative addition and deprotonation leads to Pd^II^ intermediate C. Intramolecular coordination with the enolate forms intermediate D, which can undergo reductive elimination to provide the desired benzo[*b*]furan 7 and regenerate the Pd^0^ catalyst for initiating another catalytic cycle.

In conclusion, a facile and unprecedented synthesis of 5-substituted-7-iodo-2,3-diarylbenzo[*b*]furan derivatives is reported. The reaction occurs *via* a highly regioselective, tandem α-arylation/intramolecular *O*-arylation between 5-substituted-1,2,3-triiodobenzenes and either acetophenone or phenylacetone derivatives, and the structures of the desired products were confirmed by X-ray diffraction. The reaction tolerated a variety of different of functional groups, and the products were isolated in yields up to 83%. The α-arylation reactions occurred solely at the terminal C–I bonds, as they were the least sterically-congested sites, facilitating the initial oxidative insertion by the catalyst.

In no instance was the regioisomeric product observed, and aryliodides possessing similarly-congested C–Br or C–Cl bonds were equally selective in their reactions. Finally, the highest isolated yields of the benzo[*b*]furan products were observed between electron-poor 1,2,3-triiodoarenes and electron-rich acetophenones. Various derivatives of 7 have already shown promising preliminary antimicrobial activities, and our results on this will be disclosed in due course.

## Experimental

### General

All commercial reagents and chromatography solvents were used as obtained unless otherwise stated. Ethanol, toluene, ethyl acetate, hexanes, anhydrous sodium sulfate (Na_2_SO_4_, BDH), Pd(PPh_3_)_4_ (Strem Chemicals). Anhydrous solvents were distilled over appropriate drying agents prior to use. Analytical thin layer chromatography (TLC) was performed on Merck silica gel 60 F_254_. Merck Silica gel 60 (0.063–0.2 mm) was used for column chromatography. Visualization of TLC was accomplished with UV light (254 nm). NMR spectra were recorded on a Bruker-Avance 400 MHz spectrometer. The residual solvent protons (^1^H) or the solvent carbon (^13^C) were used as internal standards. ^1^H-NMR data are presented as follows: chemical shift in ppm (*δ*) downfield from trimethylsilane (multiplicity, integration, coupling constant). The following abbreviations are used in reporting NMR data: s, singlet; bs, broad singlet; d, doublet; t, triplet; q, quartet; dq, doublet of quartets; dd, doublet of doublets; m, multiplet. High resolution mass spectra were recorded using Chemical Ionization (CI) and electrospray ionization (ESI) techniques.

### General procedure for palladium-catalyzed regioselective domino α-arylation/intramolecular *O*-arylation of 5-substituted-1,2,3-triiodoarenes and benzylketones

A flame-dried round bottom flask equipped with a condenser was charged with 5-substituted-1,2,3-triiodobenzene (5a–5i, 0.65 mmol, 1.0 equiv.), ketone (6a–6e, 0.78 mmol, 1.2 equiv.), tetrakis(triphenylphosphine)palladium(0) (20 mol%), cesium carbonate (3.0 equiv.) and 6.5 mL toluene (0.1 M). The reaction mixture was sealed with a septum, purged with argon and then heated to 130 °C for 12 h. The reaction was cooled to room temperature, diluted with 15 mL of distilled water and extracted with ethyl acetate (2 × 50 mL). The organic layers were combined and washed with brine, dried over Na_2_SO_4_, filtered and concentrated under reduced pressure. The crude product was purified by flash chromatography (15% EtOAc/hexanes) to yield the pure desired product.

#### Synthesis of 7-iodo-2,3-bis(4-methoxyphenyl)benzo[*b*]furan (7a)

The title compound was prepared using the general procedure and isolated as white solid (75% yields). IR (cast film, cm^−1^) 3017, 2922, 1589, 1575, 1208, 1128, 1027, 697. *δ*H (400 MHz, *d*-CDCl_3_) *δ*: 7.62–7.66 (m, 3H), 7.38–7.42 (m, 3H), 6.95–7.1 (m, 3H), 6.85–6.88 (d, 2H, *J* = 8.84 Hz), 3.89 (s, 3H), 3.83 (s, 3H). *δ*C (100 MHz, *d*-CDCl_3_) *δ*: 160.0, 159.0, 154.2, 151.1, 133.1, 131.0, 130.9, 128.7, 124.9, 124.6, 123.1, 119.9, 116.6, 114.7, 114.1, 74.9, 55.5, 55.4. Mp: 117–118 °C. HRMS (EI) *m*/*z* for C_22_H_17_IO_3_ [M]^+^: calcd exact 456.0222; found, 456.0217.

#### Synthesis of 7-iodo-2,3-diphenylbenzo[*b*]furan (7b)

The title compound was prepared using the general procedure and isolated as white solid (73% yields). IR (cast film, cm^−1^) 2998, 2975, 1602, 1589, 982, 702. *δ*H (400 MHz, *d*-CDCl_3_) *δ*: 7.68–7.70 (m, 3H), 7.43–7.50 (m, 6H), 7.30–7.35 (m, 3H), 7.00 (dd, 1H, *J*^*1*^ = 7.8 Hz, *J*^*2*^ = 7.7 Hz). *δ*C (100 MHz, *d*-CDCl_3_) *δ*: 154.4, 151.1, 133.7, 132.7, 130.5, 130.3, 129.9, 128.9, 128.7, 128.1, 127.3, 124.8, 120.3, 118.5. 75.0. Mp: 107–109 °C. HRMS (EI) *m*/*z* for C_20_H_13_IO [M]^+^: calcd exact 396.0011; found, 396.0008.

#### Synthesis of 2-(4-chlorophenyl)-7-iodo-3-phenylbenzo[*b*]furan (7c)

The title compound was prepared using the general procedure for and isolated as white solid (67% yields). IR (cast film, cm^−1^) 3011, 2989, 1584, 1562, 1018, 875, 771. *δ*_H_ (400 MHz, *d*-CDCl_3_) *δ*: 7.69 (d, 1H, *J* = 7.64 Hz), 7.61 (d, 2H, *J* = 8.56 Hz), 7.42–7.50 (m, 6H), 7.30 (2H), 7.00 (dd, 1H, *J*_1_ = 7.68, *J*_2_ = 7.76 Hz). *δ*C (100 MHz, *d*-CDCl_3_) *δ*: 154.4, 149.9, 134.8, 133.9, 132.4, 130.4, 129.8, 129.4, 128.9, 128.8, 128.4, 128.3, 124.9, 120.3, 118.9, 75.0. Mp: 105–107 °C. HRMS (EI) *m*/*z* for C_20_H_12_ClIO [M]^+^: calcd exact 429.9621; found, 429.9617.

#### Synthesis of 7-iodo-3-(4-methoxyphenyl)-2-methylbenzo[*b*]furan (7d)

The title compound was prepared using the general procedure and isolated as pale-yellow oil (53% yields). IR (cast film, cm^−1^) 3025, 2998, 1602, 1586, 1208, 1127, 938, 706. *δ*_H_ (400 MHz, *d*-CDCl_3_) *δ*: 7.60 (d, 1H, *J* = 7.64 Hz), 7.48 (d, 1H, *J* = 7.76 Hz), 7.39 (d, 1H, *J* = 8.48 Hz), 6.95–7.10 (m, 3H), 3.87 (s, 3H), 2.56 (s, 3H). *δ*_C_ (100 MHz, *d*-CDCl_3_) *δ*: 159.0, 151.8, 132.5, 130.2, 129.4, 124.8, 124.4, 119.6, 117.7, 114.6, 114.5, 74.8, 55.5, 13.1. HRMS (EI) *m*/*z* for C_16_H_13_IO_2_ [M]^+^: calcd 363.9960; found, 363.9956.

#### Synthesis of 7-iodo-2,3-bis(4-methoxyphenyl)-5-methylbenzo[b]furan (7e)

The title compound was prepared using the general procedure and isolated as pale-yellow oil (72% yields). IR (cast film, cm^−1^) 3051, 3007, 1208, 1157, 972, 751. *δ*_H_ (400 MHz, *d*-CDCl_3_) *δ*: 7.61 (d, 2H, *J* = 8.96 Hz), 7.47 (s, 1H), 7.38 (d, 2H, *J* = 8.68 Hz), 7.16 (s, 1H), 7.00 (d, 2H, *J* = 8.72 Hz), 6.85 (d, 2H, *J* = 8.92 Hz), 3.88 (s, 3H), 3.82 (s, 3H), 2.37 (s, 3H). *δ*_C_ (100 MHz, *d*-CDCl_3_) *δ*: 159.9, 159.3, 152.7, 151.2, 134.4, 134.1, 131.0, 130.8, 128.6, 125.2, 123.2, 119.9, 116.3, 114.7, 114.1, 74.36, 55.5, 55.4, 21.1. HRMS (EI) *m*/*z* for C_23_H_19_IO_3_ [M]^+^: calcd 470.0379; found, 470.0375.

#### Synthesis of 7-iodo-5-methyl-2,3-diphenylbenzo[*b*]furan (7f)

The title compound was prepared using the general procedure and isolated as pale-yellow oil (71% yields). IR (cast film, cm^−1^) 3022, 2937, 1602, 1586, 976, 772. *δ*_H_ (400 MHz, *d*-CDCl_3_) *δ*: 7.67 (dd, 2H, *J*_1_ = 2.16 Hz, *J*_2_ = 8.0 Hz), 7.52 (s, 1H), 7.42–7.50 (m, 5H), 7.30–7.33 (m, 3H), 7.20 (s, 1H), 2.39 (s, 3H). δ_C_ (100 MHz, *d*-CDCl_3_) *δ*: 152.9, 151.2, 134.7, 132.9, 130.4, 130.4, 129.9, 129.2, 128.8, 128.6, 127.9, 127.2, 125.7, 120.2, 118.2, 74.5, 21.1. HRMS (EI) *m*/*z* for C_21_H_15_IO [M]^+^: calcd 410.0168; found, 410.0166.

#### Synthesis of 2-(4-chlorophenyl)-7-iodo-5-methyl-3-phenylbenzo[*b*]furan (7g)

The title compound was prepared using the general procedure and isolated as pale-yellow solid (63% yields). IR (cast film, cm^−1^) 3004, 2984, 1601, 1598, 932, 695. *δ*_H_ (400 MHz, *d*-CDCl_3_) *δ*: 7.59 (d, 2H, *J* = 8.52 Hz), 7.53 (s, 3H), 7.40–7.50 (m, 5H), 7.28 (d, 2H, *J* = 8.56 Hz), 7.19 (s, 1H). *δ*C (100 MHz, *d*-CDCl_3_) *δ*: 152.9, 150.1, 134.9, 134.8, 134.6, 132.5, 130.3, 129.8, 129.3, 128.9, 128.4, 128.2, 120.2, 118.7, 74.5, 21.1. Mp: 188–189 °C. HRMS (EI) *m*/*z* for C_21_H_14_ClIO [M]^+^: calcd 443.9778; found, 443.9769.

#### Synthesis of 7-iodo-5-methoxy-2,3-bis(4-methoxyphenyl)benzo[*b*]furan (7h)

The title compound was prepared using the general procedure and isolated as colorless oil (77% yields). IR (cast film, cm^−1^) 3012, 2997, 1614, 1601, 1253, 1165, 948, 758. *δ*_H_ (400 MHz, *d*-CDCl_3_) *δ*: 7.55–7.62 (m, 2H), 7.35–7.43 (m, 3H), 7.00 (d, 2H, *J* = 8.56 Hz), 6.83–6.88 (m, 3H), 3.89 (s, 3H), 3.82 (s, 3H), 3.79 (s, 3H). *δ*C (100 MHz, *d*-CDCl_3_) *δ*: 159.9, 159.3, 156.8, 152.1, 149.5, 130.9, 128.6, 125.1, 123.2, 121.3, 114.7, 114.6, 114.1, 114.0, 103.0, 74.5, 56.3, 55.5, 55.4. HRMS (EI) *m*/*z* for C_23_H_19_IO_4_ [M]^+^: calcd exact 486.0328; found, 486.0324.

#### Synthesis of 7-iodo-5-methoxy-2,3-diphenylbenzo[*b*]furan (7i)

The title compound was prepared using the general procedure and isolated as colorless oil (67% yields). IR (cast film, cm^−1^) 3021, 2992, 1592, 1128, 984, 783. *δ*H (400 MHz, *d*-CDCl_3_) *δ*: 7.64–7.70 (m, 2H), 7.46–7.50 (m, 5H), 7.31–7.33 (m, 4H), 6.88 (d, 1H, *J* = 2.32 Hz), 3.79 (s, 3H). *δ*C (100 MHz, *d*-CDCl_3_) *δ*: 156.9, 152.1, 149.8, 132.8, 130.4, 130.3, 129.8, 129.3, 128.8, 128.6, 128.0, 127.2, 122.0, 118.7, 103.2, 74.7, 56.4. HRMS (EI) *m*/*z* for C_21_H_15_IO_2_ [M]^+^: calcd 426.0117; found, 426.0113.

#### Synthesis of 7-iodo-5-methoxy-3-(4-methoxyphenyl)-2-methyl benzo[*b*]furan (7j)

The title compound was prepared using the general procedure and isolated as colorless oil (20% yields). IR (cast film, cm^−1^) 3008, 2984, 1608, 1554, 1242, 1125, 968, 884, 745. *δ*_H_ (400 MHz, *d*-CDCl_3_) *δ*: 7.37 (d, 2H, *J* = 8.56 Hz), 7.21 (d, 1H, *J* = 2.20 Hz), 7.02 (d, 2H, *J* = 8.56 Hz), 6.95 (d, 1H, *J* = 2.16 Hz), 3.87 (s, 3H), 3.79 (s, 3H), 2.52 (s, 3H). *δ*C (100 MHz, *d*-CDCl_3_) *δ*: 159.0, 156.7, 152.8, 149.8, 130.2, 129.3, 124.9, 120.4, 117.9, 114.5, 103.2, 74.3, 56.4, 55.5, 13.1. HRMS (EI) *m*/*z* for C_17_H_15_IO_3_ [M]^+^: calcd exact 394.0066; found, 394.0060.

#### Synthesis of 5-fluoro-7-iodo-2,3-bis(4-methoxyphenyl)benzo[*b*]furan (7k)

The title compound was prepared using the general procedure and isolated as colorless oil (79% yields). IR (cast film, cm^−1^) 3010, 2997, 1588, 1548, 1154, 1113, 744. *δ*_H_ (400 MHz, *d*-CDCl_3_) *δ*: 7.62 (d, 2H, *J* = 8.68 Hz), 7.34–7.40 (m, 3H), 7.05 (dd, 2H, *J*^1^ = 2.04 Hz, *J*^2^ = 8.36 Hz), 7.00 (d, 2H, *J* = 8.64 Hz), 6.86 (d, 2H, *J* = 8.68 Hz), 3.88 (s, 3H), 3.82 (s, 3H). *δ*C (100 MHz, *d*-CDCl_3_) *δ*: 160.4, 160.3, 159.5, 158.0, 152.9, 150.9, 130.9, 128.7, 124.5, 122.8, 120.6, 120.3, 116.9, 116.8, 114.8, 114.2, 105.8, 105.5, 73.9, 73.8, 55.5, 55.4. HRMS (EI) *m*/*z* for C_22_H_16_FIO_3_ [M]^+^: calcd 474.0128; found, 474.0126.

#### Synthesis of 5-chloro-7-iodo-2,3-bis(4-methoxyphenyl)benzo[*b*]furan (7l)

The title compound was prepared using the general procedure and isolated as pale-yellow oil (68% yields). IR (cast film, cm^−1^) 3001, 2984, 1584, 1543, 1216, 1128, 857, 687. *δ*_H_ (400 MHz, *d*-CDCl_3_) *δ*: 7.60–7.64 (m, 3H), 7.34–7.40 (m, 3H), 7.00 (d, 2H, *J* = 8.44 Hz), 6.86 (d, 2H, *J* = 8.68 Hz), 3.89 (s, 3H), 3.82 (s, 3H). *δ*C (100 MHz, *d*-CDCl_3_) *δ*: 160.3, 159.5, 152.9, 152.6, 132.3, 131.6, 130.9, 129.2, 128.7, 124.3, 122.6, 119.5, 116.2, 114.8, 114.2, 74.8, 55.5, 55.4. HRMS (EI) *m*/*z* for C_22_H_16_ClIO_3_ [M]^+^: calcd 489.9833; found, 489.9829.

#### Synthesis of 5-chloro-7-iodo-2,3-diphenylbenzo[*b*]furan (7m)

The title compound was prepared using the general procedure and isolated as white solid (38% yields). IR (cast film, cm^−1^) 2995, 1604, 1589, 972, 761. *δ*_H_ (400 MHz, *d*-CDCl_3_) *δ*: 7.60–7.70 (m, 3H), 7.40–7.50 (m, 6H), 7.30–7.35 (m, 3H). *δ*_C_ (100 MHz, *d*-CDCl_3_) *δ*: 153.2, 152.6, 132.9, 132.0, 131.2, 129.8, 129.7, 129.5, 129.4, 129.3, 128.7, 128.4, 125.7, 119.9, 118.1, 75.0. Mp: 105–106 °C. HRMS (EI) *m*/*z* for C_20_H_12_ClIO [M]^+^: calcd 429.9621; found, 429.9618.

#### Synthesis of 2-benzyl-5-chloro-7-iodo-3-phenylbenzo[*b*]furan (7n)

The title compound was prepared using the general procedure and isolated as pale-yellow oil (66% yields). IR (cast film, cm^−1^) 3024, 2994, 1599, 1584, 861, 782. *δ*_H_ (400 MHz, *d*-CDCl_3_) *δ*: 7.62 (d, 1H, *J* = 1.72 Hz), 7.39–7.55 (m, 6H), 7.23–7.35 (m, 5H), 4.22 (s, 2H). *δ*C (100 MHz, *d*-CDCl_3_) *δ*: 154.9, 153.6, 137.3, 132.4, 131.6, 129.7, 129.3, 129.2, 129.1, 128.9, 128.7, 128.0, 126.9, 119.7, 119.1, 75.1, 33.1. HRMS (EI) *m*/*z* for C_21_H_14_ClIO [M]^+^: calcd 443.9778; found, 443.9775.

#### Synthesis of 5-bromo-7-iodo-2,3-bis(4-methoxyphenyl)benzo[*b*]furan (7p)

The title compound was prepared using the general procedure and isolated as pale-yellow oil (71% yields). IR (cast film, cm^−1^) 2998, 1605, 1592, 1281, 1186, 864, 731. *δ*_H_ (400 MHz, *d*-CDCl_3_) *δ*: 7.74 (d, 1H, *J* = 1.6 Hz), 7.61 (d, 2H, *J* = 8.8 Hz), 7.49 (d, 1H, *J* = 1.5 Hz), 7.35 (d, 2H, s, 2H, *J* = 8.56 Hz), 7.00 (d, 2H, *J* = 8.6 Hz), 6.86 (d, 2H, *J* = 9.43 Hz), 3.88 (s, 3H), 3.82 (s, 3H). *δ*C (100 MHz, *d*-CDCl_3_) *δ*: 160.3, 159.6, 153.3, 152.4, 134.7, 132.3, 130.9, 128.7, 128.6, 124.2, 122.5, 116.5, 116.1, 114.8, 114.2, 75.4, 55.5, 55.4. HRMS (EI) *m*/*z* for C_22_H_16_BrIO_3_ [M]^+^: calcd 533.9327; found, 533.9322.

#### Synthesis of methyl 7-iodo-2,3-bis(4-methoxyphenyl)benzo[*b*]furan-5-carboxylate (7q)

The title compound was prepared using the general procedure and isolated as colorless oil (83% yields). IR (cast film, cm^−1^) 3017, 2989, 1756, 1612, 1588, 1260, 1228, 837, 731. *δ*H (400 MHz, *d*-CDCl_3_) *δ*: 8.36 (s, 1H), 8.01 (s, 1H), 7.63 (d, 2H, *J* = 8.44 Hz), 7.39 (d, 2H, *J* = 8.28 Hz), 7.02 (d, 2H, *J* = 8.24 Hz), 6.87 (d, 2H, *J*^1^ = 8.48 Hz), 3.91 (s, 3H), 3.89 (s, 3H), 3.82 (s, 3H). *δ*C (100 MHz, *d*-CDCl_3_) *δ*: 166.3, 160.3, 159.6, 156.7, 152.4, 134.6, 131.0, 130.7, 128.7, 127.0, 124.2, 122.5, 122.2, 116.9, 114.9, 114.2, 74.5, 55.5, 55.4, 52.4. HRMS (EI) *m*/*z* for C_24_H_19_IO_5_ [M]^+^: calcd 514.0277; found, 514.0273.

#### Synthesis of 7-chloro-2,3-bis(4-methoxyphenyl)benzo[*b*]furan (7r)

The title compound was prepared using the general procedure and isolated as colorless oil (77% yields). IR (cast film, cm^−1^) 3013, 2984, 1609, 1591, 1268, 1153, 855, 694. *δ*_H_ (400 MHz, *d*-CDCl_3_) *δ*: 7.63 (d, 2H, *J* = 8.64 Hz), 7.39 (d, 2H, *J* = 8.36 Hz), 7.34 (d, 1H, *J* = 7.72 Hz), 7.28 (dd, 1H, *J*^1^ = 7.76 Hz, *J*^2^ = 6.8 Hz), 7.15 (t, 1H, *J* = 7.76 Hz), 7.01 (d, 2H, *J* = 8.4 Hz), 6.86 (d, 2H, *J* = 8.64 Hz), 3.89 (s, 3H), 3.82 (s, 3H). *δ*C (100 MHz, *d*-CDCl_3_) *δ*: 16.1, 159.4, 151.6, 149.7, 132.5, 131.0, 128.7, 124.8, 124.4, 123.8, 123.0, 118.4, 116.6, 116.2, 114.7, 114.1, 55.5, 55.4. HRMS (EI) *m*/*z* for C_22_H_17_ClO_3_ [M]^+^: calcd 364.0866; found, 364.0865.

#### Synthesis of 7-chloro-2,3-diphenylbenzo[*b*]furan (7s)

The title compound was prepared using the general procedure for palladium-catalyzed regioselective domino α-arylation/intramolecular *O*-arylation reaction and isolated as white solid (71% yields). The spectroscopic data for this compound are matched the previous report by W. Zeng, W. Wu, H. Jiang, L. Huang, Y. Sun, Z. Chen and X. Li, *Chem. Commun.*, 2013, **49**, 6611–6613.

#### Synthesis of 7-bromo-2,3-bis(4-methoxyphenyl)benzo[*b*]furan (7t)

The title compound was prepared using the general procedure and isolated as pale-yellow oil (73% yields). IR (cast film, cm^−1^) 3005, 2998, 1601, 1576, 1291, 1183, 911, 844. *δ*_H_ (400 MHz, *d*-CDCl_3_) *δ*: 7.63 (d, 2H, *J* = 8.88 Hz), 7.37–7.45 (m, 4H), 7.09 (t, 1H, *J* = 7.76 Hz), 7.00 (d, 2H, *J* = 8.68 Hz), 6.86 (d, 2H, *J* = 8.88 Hz), 3.89 (s, 3H), 3.82 (s, 3H). *δ*C (100 MHz, *d*-CDCl_3_) *δ*: 160.1, 159.4, 151.5, 151.1, 132.1, 131.0, 128.7, 127.2, 124.8, 124.2, 123.0, 119.0, 116.3, 114.7, 114.1, 103.9, 55.5, 55.4. HRMS (EI) *m*/*z* for C_22_H_17_BrO_3_ [M]^+^: calcd 408.0361; found, 408.0355.

#### Synthesis of 7-bromo-2,3-diphenylbenzo[*b*]furan (7u)

The title compound was prepared using the general procedure for palladium-catalyzed regioselective domino α-arylation/intramolecular *O*-arylation reaction and isolated as white solid (72% yields). The spectroscopic data for this compound are matched the previous report by W. Zeng, W. Wu, H. Jiang, L. Huang, Y. Sun, Z. Chen and X. Li, *Chem. Commun.*, 2013, **49**, 6611–6613.

## Conflicts of interest

There are no conflicts to declare.

## Supplementary Material

RA-011-D1RA05730H-s001

RA-011-D1RA05730H-s002
