# Supplementary material for: Domino C–C/C–O bond formation: palladium-catalyzed regioselective synthesis of 7-iodobenzo[b]furans using 1,2,3-triiodobenzenes and benzylketones
Source: RSC Adv. 2021 Sep 8;11(48):30069–77. doi: 10.1039/d1ra05730h (PMC9040925; doi:10.1039/d1ra05730h)
Supplement: RA-011-D1RA05730H-s001 [file RA-011-D1RA05730H-s001.pdf]

Electronic Supplementary Material (ESI) for RSC Advances.
This journal is © The Royal Society of Chemistry 2021

Jordan University of Science and Technology – Department of Chemistry
University of Waterloo – Department of Chemistry

DOMINO C-C/C-O BOND FORMATION: PALLADIUM-CATALYZED REGIOSELECTIVE SYNTHESIS OF 7-IODOBENZO[B]FURANS USING 1,2,3-TRIIODOBENZENES AND BENZYLKETONES

Supporting Information

Table of Contents

Table of Contents i

1. Experimental Details and Compound Data 1

1.1 General Information 1

1.2 General procedure for palladium-catalyzed regioselective domino α-arylation/intramolecular *O*-arylation of 5-substituted-1,2,3-triiodoarenes 1

1.2.1 *Synthesis of 7-iodo-2,3-bis(4-methoxyphenyl)benzo[b]furan* (**7a**) 2
1.2.2 *Synthesis of 7-iodo-2,3-diphenylbenzo[b]furan* (**7b**) 2
1.2.3 *Synthesis of 2-(4-chlorophenyl)-7-iodo-3-phenylbenzo[b]furan* (**7c**) 3
1.2.4 *Synthesis of 7-iodo-3-(4-methoxyphenyl)-2-methylbenzo[b]furan* (**7d**) 3
1.2.5 *Synthesis of 7-iodo-2,3-bis(4-methoxyphenyl)-5-methylbenzo[b]furan* (**7e**) 3
1.2.6 *Synthesis of 7-iodo-5-methyl-2,3-diphenylbenzo[b]furan* (**7f**) 4
1.2.7 *Synthesis of 2-(4-chlorophenyl)-7-iodo-5-methyl-3-phenylbenzo[b]furan* (**7g**) 4
1.2.8 *Synthesis of 7-iodo-5-methoxy-2,3-bis(4-methoxyphenyl)benzo[b]fur*an (**7h**) 5
1.2.9 *Synthesis of 7-iodo-5-methoxy-2,3-diphenylbenzo[b]furan* (**7i**) 5
1.2.10 *Synthesis of 7-iodo-5-methoxy-3-(4-methoxyphenyl)-2-methylbenzo[b]furan* (**7j**) 6
1.2.11 *Synthesis of 5-fluoro-7-iodo-2,3-bis(4-methoxyphenyl)benzo[b]furan* (**7k**) 6
1.2.12 *Synthesis of 5-chloro-7-iodo-2,3-bis(4-methoxyphenyl)benzo[b]furan* (**7l**) 7
1.2.13 *Synthesis of 5-chloro-7-iodo-2,3-diphenylbenzo[b]furan* (**7m**) 7
1.2.14 *Synthesis of 2-benzyl-5-chloro-7-iodo-3-phenylbenzo[b]furan* (**7n**) 7
1.2.15 *Synthesis of 5-bromo-7-iodo-2,3-bis(4-methoxyphenyl)benzo[b]furan* (**7p**) 8
1.2.16 *Synthesis of methyl 7-iodo-2,3-bis(4-methoxyphenyl)benzo[b]furan-5-carboxylate* (**7q**) 8
1.2.17 *Synthesis of 7-chloro-2,3-bis(4-methoxyphenyl)benzo[b]furan* (**7r**) 10
1.2.18 *Synthesis of 7-chloro-2,3-diphenylbenzo[b]furan* (**7s**) 10
1.2.19 *Synthesis of 7-bromo-2,3-bis(4-methoxyphenyl)benzo[b]furan* (**7t**) 9
1.2.20 *Synthesis of 7-bromo-2,3-diphenylbenzo[b]furan* (**7u**) 9
1.2.21 *Synthesis of 1-(5-chloro-2,3-diiodophenyl)-1-(4-methoxyphenyl)propan-2-one* 10

1.3 NMR Spectra for New Compounds 12

1.3.1 *^{1}H-NMR of 7-iodo-2,3-bis(4-methoxyphenyl)benzo[b]furan (**7a**) in d-$CDCl_3$ at 25 °C.* 12
1.3.2 *^{13}C-NMR of 7-iodo-2,3-bis(4-methoxyphenyl)benzo[b]furan (**7a**) in d-$CDCl_3$ at 25 °C.* 13
1.3.3 *^{1}H-NMR of 7-iodo-2,3-diphenylbenzo[b]furan (**7b**) in d-$CDCl_3$ at 25 °C.* 14
1.3.4 *^{13}C-NMR of 7-iodo-2,3-diphenylbenzo[b]furan (**7b**) in d-$CDCl_3$ at 25 °C.* 15
1.3.5 *^{1}H-NMR of 2-(4-chlorophenyl)-7-iodo-3-phenylbenzo[b]furan (**7c**) in d-$CDCl_3$ at 25 °C.* 16
1.3.6 *^{13}C-NMR of 2-(4-chlorophenyl)-7-iodo-3-phenylbenzo[b]furan (**7c**) in d-$CDCl_3$ at 25 °C.* 17
1.3.7 *^{1}H-NMR of 7-iodo-3-(4-methoxyphenyl)-2-methylbenzofuran (**7d**) in d-$CDCl_3$ at 25 °C* 18
1.3.8 *^{13}C-NMR of 7-iodo-3-(4-methoxyphenyl)-2-methylbenzo[b]furan (**7d**) in d-$CDCl_3$ at 25 °C* 19
1.3.9 *^{1}H-NMR of 7-iodo-2,3-bis(4-methoxyphenyl)-5-methylbenzo[b]furan (**7e**) in d-$CDCl_3$ at 25 °C* 20
1.3.10 ^{13}C-NMR of 7-iodo-2,3-bis(4-methoxyphenyl)-5-methylbenzo[b]furan (**7e**) in d-$CDCl_3$ at 25 °C. 21
1.3.11 *^{1}H-NMR of 7-iodo-5-methyl-2,3-diphenylbenzo[b]furan (**7f**) in d-$CDCl_3$ at 25 °C.* 22
1.3.12 *^{13}C-NMR of 7-iodo-5-methyl-2,3-diphenylbenzo[b]furan (**7f**) in d-$CDCl_3$ at 25 °C.* 23
1.3.13 *^{1}H-NMR of 2-(4-chlorophenyl)-7-iodo-5-methyl-3-phenylbenzo[b]furan (**7g**) in d-$CDCl_3$ at 25 °C.* 24
1.3.14 *^{13}C-NMR of 2-(4-chlorophenyl)-7-iodo-5-methyl-3-phenylbenzo[b]furan (**7g**) in d-$CDCl_3$ at 25 °C.* 25
1.3.15 *^{1}H-NMR of 7-iodo-5-methoxy-2,3-bis(4-methoxyphenyl)benzo[b]furan (**7h**) in d-$CDCl_3$ at 25 °C.* 26
1.3.16 *^{13}C-NMR of 7-iodo-5-methoxy-2,3-bis(4-methoxyphenyl)benzo[b]furan (**7h**) in d-$CDCl_3$ at 25 °C.* 27
1.3.17 *^{1}H-NMR of 7-iodo-5-methoxy-2,3-diphenylbenzo[b]furan (**7i**) in d-$CDCl_3$ at 25 °C.* 28
1.3.18 *^{13}C-NMR of 7-iodo-5-methoxy-2,3-diphenylbenzo[b]furan (**7i**) in d-$CDCl_3$ at 25 °C.* 29
1.3.19 *^{1}H-NMR of 7-iodo-5-methoxy-3-(4-methoxyphenyl)-2-methylbenzo[b]furan (**7j**) in d-$CDCl_3$ at 25 °C.* 30

1.3.20 *^{13}C-NMR of 7-iodo-5-methoxy-3-(4-methoxyphenyl)-2-methylbenzo[b]furan (**7j**) in d-CDCl$_3$ at 25 ^{o}C.* 31
1.3.21 *^{1}H-NMR of 5-fluoro-7-iodo-2,3-bis(4-methoxyphenyl)benzo[b]furan nzofuran (**7k**) in d-CDCl$_3$ at 25 ^{o}C* 32
1.3.22 *^{13}C-NMR of 5-fluoro-7-iodo-2,3-bis(4-methoxyphenyl)benzo[b]furan (**7k**) in d-CDCl$_3$ at 25 ^{o}C.* 33
1.3.23 *^{1}H-NMR of 5-chloro-7-iodo-2,3-bis(4-methoxyphenyl)benzo[b]furan (**7l**) in d-CDCl$_3$ at 25 ^{o}C* 34
1.3.24 *^{13}C-NMR of 5-chloro-7-iodo-2,3-bis(4-methoxyphenyl)benzo[b]furan (**7l**) in d-CDCl$_3$ at 25 ^{o}C.* 35
1.3.25 *^{1}H-NMR of 5-chloro-7-iodo-2,3-diphenylbenzo[b]furan (**7m**) in d-CDCl$_3$ at 25 ^{o}C* 36
1.3.26 *^{13}C-NMR of 5-chloro-7-iodo-2,3-diphenylbenzo[b]furan (**7m**) in d-CDCl$_3$ at 25 ^{o}C.* 37
1.3.27 *^{1}H-NMR of 2-benzyl-5-chloro-7-iodo-3-phenylbenzo[b]furan (**7n**) in d-CDCl$_3$ at 25 ^{o}C* 38
1.3.28 *^{13}C-NMR of 2-benzyl-5-chloro-7-iodo-3-phenylbenzo[b]furan (**7n**) in d-CDCl$_3$ at 25 ^{o}C.* 39
1.3.1 *^{1}H-NMR of 5-bromo-7-iodo-2,3-bis(4-methoxyphenyl)benzo[b]furan (**7p**) in d-CDCl$_3$ at 25 ^{o}C* 40
1.3.2 *^{13}C-NMR of 5-bromo-7-iodo-2,3-bis(4-methoxyphenyl)benzo[b]furan (**7p**) in d-CDCl$_3$ at 25 ^{o}C.* 41
1.3.3 *^{1}H-NMR of methyl 7-iodo-2,3-bis(4-methoxyphenyl)benzo[b]furan-5-carboxylate (**7q**) in d-CDCl$_3$ at 25 ^{o}C....* 42
1.3.4 *^{13}C-NMR of methyl 7-iodo-2,3-bis(4-methoxyphenyl)benzo[b]furan-5-carboxylate (**7q**) in d-CDCl$_3$ at 25 ^{o}C...* 43
1.3.1 *^{1}H-NMR of 7-chloro-2,3-bis(4-methoxyphenyl)benzo[b]furan (**7r**) in d-CDCl$_3$ at 25 ^{o}C* 46
1.3.2 *^{13}C-NMR of 7-chloro-2,3-bis(4-methoxyphenyl)benzo[b]furan (**7r**) in d-CDCl$_3$ at 25 ^{o}C.* 47
1.3.3 *^{1}H-NMR of 7-bromo-2,3-bis(4-methoxyphenyl)benzo[b]furan (**7t**) in d-CDCl$_3$ at 25 ^{o}C* 44
1.3.4 *^{13}C-NMR of 7-bromo-2,3-bis(4-methoxyphenyl)benzo[b]furan (**7t**) in d-CDCl$_3$ at 25 ^{o}C.* 45
1.3.5 *^{1}H-NMR of 1-(5-chloro-2,3-diiodophenyl)-1-(4-methoxyphenyl)propan-2-one in d-CDCl$_3$ at 25 ^{o}C* 48
1.3.6 *^{13}C-NMR of 1-(5-chloro-2,3-diiodophenyl)-1-(4-methoxyphenyl)propan-2-one in d-CDCl$_3$ at 25 ^{o}C.* 49

1.4 X-ray of new compounds 50

1.4.1 *X-ray data of 2-(4-chlorophenyl)-7-iodo-3-phenyl-1-benzo[b]furan (**7c**)* 50
1.4.2 *X-ray data of 2-(4-chlorophenyl)-7-iodo-5-methyl-3-phenyl-1-benzo[b]furan (**7g**)* 62

1. Experimental Details and Compound Data

1.1 General Information

All commercial reagents and chromatography solvents were used as obtained unless otherwise stated. Ethanol, toluene, ethyl acetate, hexanes, anhydrous sodium sulfate (Na_2SO_4, BDH). Anhydrous solvents were distilled over appropriate drying agents prior to use. Analytical thin layer chromatography (TLC) was performed on Merck silica gel 60 F_{254}. Merck Silica gel 60 (0.063 - 0.2 mm) was used for column chromatography. Visualization of TLC was accomplished with UV light (254 nm). NMR spectra were recorded on a Bruker-Avance 400 MHz spectrometer. The residual solvent protons (^1H) or the solvent carbon (^{13}C) were used as internal standards. ^1H-NMR data are presented as follows: chemical shift in ppm (δ) downfield from trimethylsilane (multiplicity, integration, coupling constant). The following abbreviations are used in reporting NMR data: s, singlet; bs, broad singlet; d, doublet; t, triplet; q, quartet; dq, doublet of quartets; dd, doublet of doublets; m, multiplet. High resolution mass spectra were recorded using Chemical Ionization (CI) and Electrospray ionization (ESI) techniques.

1.2 General procedure for palladium-catalyzed regioselective domino α-arylation/intramolecular O-arylation of 5-substituted-1,2,3-triiodoarenes and benzylketones

A flame-dry round bottom flask with condenser was charged with 5-substituted-1,2,3-triiodobenzene (0.65 mmol, 1.0 equiv.), benzylketone (0.78 mmol, 1.2 equiv.), tetrakis-(triphenylphosphine)palladium(0) (20 mol%), cesium carbonate (3.0 equiv.) and 6.5 mL toluene (0.1M). The reaction mixture was sealed with sepata, purged with argon

and then heated to 130 °C for 12 h. The reaction was cooled down to room temperature, 15 mL of distilled water was added and extracted with ethyl acetate (2 x 50 mL). The organic layers were combined and washed with brine, dried with Na_2SO_4, filtered and then concentrated under reduced pressure. The crude product was purified by flash chromatography (15% EthOAc/Hexanes) to yield the pure desired product.

1.2.1 Synthesis of 7-iodo-2,3-bis(4-methoxyphenyl)benzo[b]furan (7a)

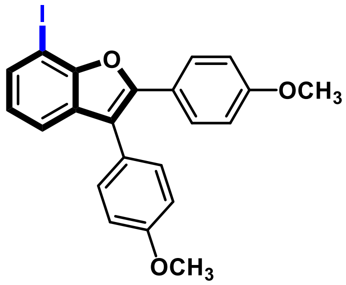

The title compound was prepared using the general procedure and isolated as white solid (**75%** yields). **IR** (cast film, cm^{-1}) 3017, 2922, 1589, 1575, 1208, 1128, 1027, 697. **δ_H** (400 MHz, *d*-$CDCl_3$) δ: 7.62-7.66 (m, 3H), 7.38-7.42 (m, 3H), 6.95-7.1 (m, 3H), 6.85-6.88 (d, 2H, *J* = 8.84 Hz), 3.89 (s, 3H), 3.83 (s, 3H). **δ_C** (100 MHz, *d*-$CDCl_3$) δ: 160.0, 159.0, 154.2, 151.1, 133.1, 131.0, 130.9, 128.7, 124.9, 124.6, 123.1, 119.9, 116.6, 114.7, 114.1, 74.9, 55.5, 55.4. **M.p**: 117-118°C. **HRMS** (EI) m/z for $C_{22}H_{17}IO_3$ $[M]^+$: calcd. Exact 456.0222; found, 456.0217

1.2.2 Synthesis of 7-iodo-2,3-diphenylbenzo[b]furan (7b)

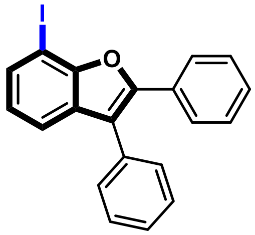

The title compound was prepared using the general procedure and isolated as white solid (**73%** yields). **IR** (cast film, cm^{-1}) 2998, 2975, 1602, 1589, 982, 702. **δ_H** (400 MHz, *d*-$CDCl_3$) δ: 7.68-7.70 (m, 3H), 7.43-7.50 (m, 6H), 7.30-7.35 (m, 3H), 7.00 (dd, 1H, *J* = 7.8 Hz, *J* = 7.7 Hz). **δ_C** (100 MHz, *d*-$CDCl_3$) δ: 154.4, 151.1, 133.7, 132.7, 130.5, 130.3, 129.9, 128.9, 128.7, 128.1, 127.3, 124.8, 120.3, 118.5. 75.0. **M.p**: 107-109°C. **HRMS** (EI) m/z for $C_{20}H_{13}IO$ $[M]^+$: calcd. Exact 396.0011; found, 396.0008.

1.2.3 Synthesis of 2-(4-chlorophenyl)-7-iodo-3-phenylbenzo[b]furan (7c)

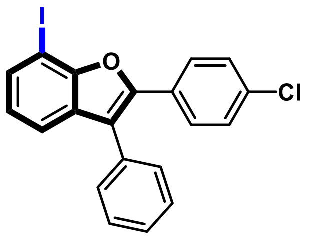

The title compound was prepared using the general procedure for and isolated as white solid (**67%** yields). **IR** (cast film, cm^{-1}) 3011, 2989, 1584, 1562, 1018, 875, 771. **δH** (400 MHz, *d*-CDCl$_3$) δ: 7.69 (d, 1H, *J* = 7.64 Hz), 7.61 (d, 2H, *J* = 8.56 Hz), 7.42-7.50 (m, 6H), 7.30 (2H), 7.00 (dd, 1H, J_1 = 7.68, J_2 = 7.76 Hz). **δC** (100 MHz, *d*-CDCl$_3$) δ: 154.4, 149.9, 134.8, 133.9, 132.4, 130.4, 129.8, 129.4, 128.9, 128.8, 128.4, 128.3, 124.9, 120.3, 118.9, 75.0. **M.p**: 105-107°C. **HRMS** (EI) m/z for $C_{20}H_{12}ClIO$ [M]$^+$: calcd. Exact 429.9621; found, 429.9617.

1.2.4 Synthesis of 7-iodo-3-(4-methoxyphenyl)-2-methylbenzo[b]furan (7d)

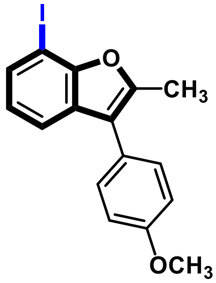

The title compound was prepared using the general procedure and isolated as pale-yellow oil (**53%** yields). **IR** (cast film, cm^{-1}) 3025, 2998, 1602, 1586, 1208, 1127, 938, 706. **δH** (400 MHz, *d*-CDCl$_3$) δ: 7.60 (d, 1H, *J* = 7.64 Hz), 7.48 (d, 1H, *J* = 7.76 Hz), 7.39 (d, 1H, *J* = 8.48 Hz), 6.95-7.10 (m, 3H), 3.87 (s, 3H), 2.56 (s, 3H). **δC** (100 MHz, *d*-CDCl$_3$) δ: 159.0, 151.8, 132.5, 130.2, 129.4, 124.8, 124.4, 119.6, 117.7, 114.6, 114.5, 74.8, 55.5, 13.1. **HRMS** (EI) m/z for $C_{16}H_{13}IO_2$ [M]$^+$: calcd. 363.9960; found, 363.9956.

1.2.5 Synthesis of 7-iodo-2,3-bis(4-methoxyphenyl)-5-methylbenzo[b]furan (7e)

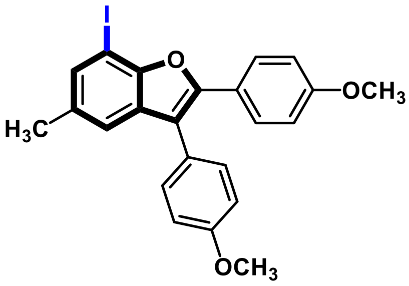

The title ompound was prepared using the general procedure and isolated as pale-yellow oil (**72%** yields). **IR** (cast film, cm^{-1}) 3051, 3007, 1208, 1157, 972, 751. **δH** (400 MHz, *d*-CDCl$_3$) δ: 7.61 (d, 2H, *J* = 8.96 Hz), 7.47 (s,

1H), 7.38 (d, 2H, *J* = 8.68 Hz), 7.16 (s, 1H), 7.00 (d, 2H, *J* = 8.72 Hz), 6.85 (d, 2H, *J* = 8.92 Hz), 3.88 (s, 3H), 3.82 (s, 3H), 2.37 (s, 3H). **δc** (100 MHz, *d*-$CDCl_3$) δ: 159.9, 159.3, 152.7, 151.2, 134.4, 134.1, 131.0, 130.8, 128.6, 125.2, 123.2, 119.9, 116.3, 114.7, 114.1, 74.36, 55.5, 55.4, 21.1. **HRMS** (EI) m/z for $C_{23}H_{19}IO_3$ $[M]^+$: calcd. 470.0379; found, 470.0375.

1.2.6 Synthesis of 7-iodo-5-methyl-2,3-diphenylbenzo[b]furan (7f)

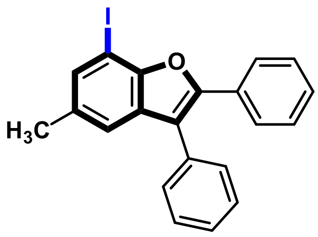

The title compound was prepared using the general procedure and isolated as pale-yellow oil (**71%** yields). **IR** (cast film, cm^{-1}) 3022, 2937, 1602, 1586, 976, 772. **δH** (400 MHz, *d*-$CDCl_3$) δ: 7.67 (dd, 2H, J_1 = 2.16Hz, J_2 = 8.0Hz), 7.52 (s, 1H), 7.42-7.50 (m, 5H), 7.30-7.33 (m, 3H), 7.20 (s, 1H), 2.39 (s, 3H). **δc** (100 MHz, *d*-$CDCl_3$) δ: 152.9, 151.2, 134.7, 132.9, 130.4, 130.4, 129.9, 129.2, 128.8, 128.6, 127.9, 127.2, 125.7, 120.2, 118.2, 74.5, 21.1. **HRMS** (EI) m/z for $C_{21}H_{15}IO$ $[M]^+$: calcd. 410.0168; found, 410.0166.

1.2.7 Synthesis of 2-(4-chlorophenyl)-7-iodo-5-methyl-3-phenylbenzo[b]furan (7g)

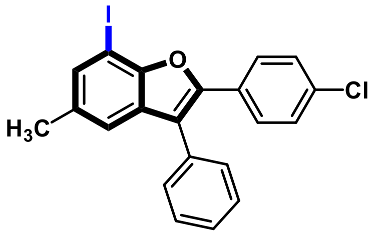

The title compound was prepared using the general procedure and isolated as pale-yellow solid (**63%** yields). **IR** (cast film, cm^{-1}) 3004, 2984, 1601, 1598, 932, 695. **δH** (400 MHz, *d*-$CDCl_3$) δ: 7.59 (d, 2H, J = 8.52 Hz), 7.53 (s, 3H), 7.40-7.50 (m, 5H), 7.28 (d, 2H, J = 8.56Hz), 7.19 (s, 1H). **δc** (100 MHz, *d*-$CDCl_3$) δ: 152.9, 150.1, 134.9, 134.8, 134.6, 132.5, 130.3, 129.8, 129.3, 128.9, 128.4, 128.2, 120.2, 118.7, 74.5, 21.1. **M.p**: 188-189°C. **HRMS** (EI) m/z for $C_{21}H_{14}ClIO$ $[M]^+$: calcd. 443.9778; found, 443.9769.

1.2.8 Synthesis of 7-iodo-5-methoxy-2,3-bis(4-methoxyphenyl)benzo[b]furan (7h)

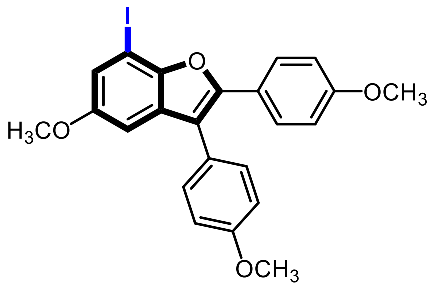

The title compound was prepared using the general procedure and isolated as colorless oil (**77%** yields). **IR** (cast film, cm^{-1}) 3012, 2997, 1614, 1601, 1253, 1165, 948, 758. **δ_H** (400 MHz, *d*-$CDCl_3$) δ: 7.55-7.62 (m, 2H), 7.35-7.43 (m, 3H), 7.00 (d, 2H, *J* = 8.56 Hz), 6.83-6.88 (m, 3H), 3.89 (s, 3H), 3.82 (s, 3H), 3.79 (s, 3H). **δ_C** (100 MHz, *d*-$CDCl_3$) δ:159.9, 159.3, 156.8, 152.1, 149.5, 130.9, 128.6, 125.1, 123.2, 121.3, 114.7, 114.6, 114.1, 114.0, 103.0, 74.5, 56.3, 55.5, 55.4. **HRMS** (EI) m/z for $C_{23}H_{19}IO_4$ $[M]^+$: calcd. Exact 486.0328; found, 486.0324.

1.2.9 Synthesis of 7-iodo-5-methoxy-2,3-diphenylbenzo[b]furan (7i)

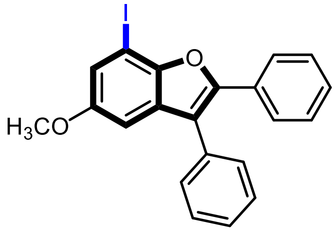

The title compound was prepared using the general procedure and isolated as colorless oil (**67%** yields). **IR** (cast film, cm^{-1}) 3021, 2992, 1592, 1128, 984, 783. **δ_H** (400MHz, *d*-$CDCl_3$) δ: 7.64-7.70 (m, 2H), 7.46-7.50 (m, 5H), 7.31-7.33 (m, 4H), 6.88 (d, 1H, *J* = 2.32 Hz), 3.79 (s, 3H). **δ_C** (100 MHz, *d*-$CDCl_3$) δ: 156.9, 152.1, 149.8, 132.8, 130.4, 130.3, 129.8, 129.3, 128.8, 128.6, 128.0, 127.2, 122.0, 118.7, 103.2, 74.7, 56.4. **HRMS** (EI) m/z for $C_{21}H_{15}IO_2$ $[M]^+$: calcd. 426.0117; found, 426.0113.

1.2.10 *Synthesis of 7-iodo-5-methoxy-3-(4-methoxyphenyl)-2-methyl benzo[b]furan (7j)*

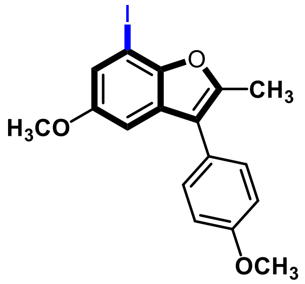

The title compound was prepared using the general procedure and isolated as colorless oil (**20%** yields). **IR** (cast film, cm^{-1}) 3008, 2984, 1608, 1554, 1242, 1125, 968, 884, 745. **δ_H** (400 MHz, *d*-$CDCl_3$) δ: 7.37 (d, 2H, *J* = 8.56 Hz), 7.21 (d, 1H, *J* = 2.20 Hz), 7.02 (d, 2H, *J* = 8.56 Hz), 6.95 (d, 1H, *J* = 2.16 Hz), 3.87 (s, 3H), 3.79 (s, 3H), 2.52 (s, 3H). **δ_C** (100 MHz, *d*-$CDCl_3$) δ: 159.0, 156.7, 152.8, 149.8, 130.2, 129.3, 124.9, 120.4, 117.9, 114.5, 103.2, 74.3, 56.4, 55.5, 13.1. **HRMS** (EI) m/z for $C_{17}H_{15}IO_3$ $[M]^+$: calcd. Exact 394.0066; found, 394.0060.

1.2.11 *Synthesis of 5-fluoro-7-iodo-2,3-bis(4-methoxyphenyl)benzo[b]furan (7k)*

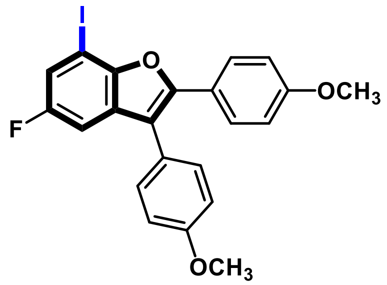

The title compound was prepared using the general procedure and isolated as colorless oil (**79%** yields). **IR** (cast film, cm^{-1}) 3010, 2997, 1588, 1548, 1154, 1113, 744. **δ_H** (400 MHz, *d*-$CDCl_3$) δ: 7.62 (d, 2H, *J* = 8.68 Hz), 7.34-7.40 (m, 3H), 7.05 (dd, 2H, J^1 = 2.04 Hz, J^2 = 8.36 Hz), 7.00 (d, 2H, *J* = 8.64 Hz), 6.86 (d, 2H, *J* = 8.68 Hz), 3.88 (s, 3H), 3.82 (s, 3H). **δ_C** (100 MHz, *d*-$CDCl_3$) δ: 160.4, 160.3, 159.5, 158.0, 152.9, 150.9, 130.9, 128.7, 124.5, 122.8, 120.6, 120.3, 116.9, 116.8, 114.8, 114.2, 105.8, 105.5, 73.9, 73.8, 55.5, 55.4. **HRMS** (EI) m/z for $C_{22}H_{16}FIO_3$ $[M]^+$: calcd. 474.0128; found, 474.0126.

1.2.12 Synthesis of 5-chloro-7-iodo-2,3-bis(4-methoxyphenyl)benzo[b]furan (7l)

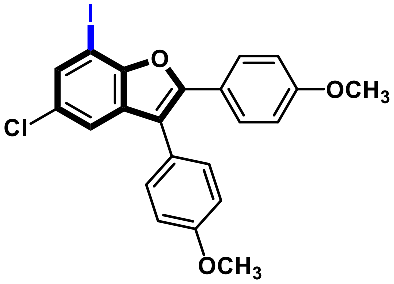

The title compound was prepared using the general procedure and isolated as pale-yellow oil (**68%** yields). **IR** (cast film, cm^{-1}) 3001, 2984, 1584, 1543, 1216, 1128, 857, 687. **δH** (400 MHz, *d*-$CDCl_3$) δ: 7.60-7.64 (m, 3H), 7.34-7.40 (m, 3H), 7.00 (d, 2H, *J* = 8.44 Hz), 6.86 (d, 2H, *J* = 8.68 Hz), 3.89 (s, 3H), 3.82 (s, 3H). **δC** (100 MHz, *d*-$CDCl_3$) δ: 160.3, 159.5, 152.9, 152.6, 132.3, 131.6, 130.9, 129.2, 128.7, 124.3, 122.6, 119.5, 116.2, 114.8, 114.2, 74.8, 55.5, 55.4. **HRMS** (EI) m/z for $C_{22}H_{16}ClIO_3$ $[M]^+$: calcd. 489.9833; found, 489.9829.

1.2.13 Synthesis of 5-chloro-7-iodo-2,3-diphenylbenzo[b]furan (7m)

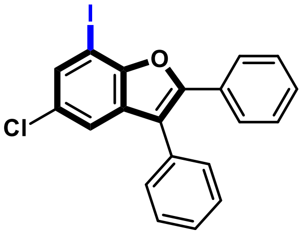

The title compound was prepared using the general procedure and isolated as white solid (**38%** yields). **IR** (cast film, cm^{-1}) 2995, 1604, 1589, 972, 761. **δH** (400 MHz, *d*-$CDCl_3$) δ: 7.60-7.70 (m, 3H), 7.40-7.50 (m, 6H), 7.30-7.35 (m, 3H). **δC** (100 MHz, *d*-$CDCl_3$) δ: 153.2, 152.6, 132.9, 132.0, 131.2, 129.8, 129.7, 129.5, 129.4, 129.3, 128.7, 128.4, 125.7, 119.9, 118.1, 75.0. **M.p**: 105-106 °C. **HRMS** (EI) m/z for $C_{20}H_{12}ClIO$ $[M]^+$: calcd. 429.9621; found, 429.9618.

1.2.14 Synthesis of 2-benzyl-5-chloro-7-iodo-3-phenylbenzo[b]furan (7n)

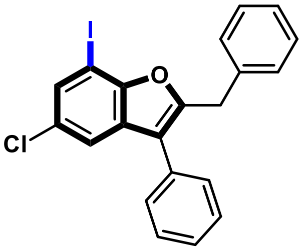

The title compound was prepared using the general procedure and isolated as pale-yellow oil (**66%** yields). **IR** (cast film, cm^{-1}) 3024, 2994, 1599, 1584, 861, 782. **δH** (400 MHz, *d*-$CDCl_3$) δ:

7.62 (d, 1H, *J* = 1.72 Hz), 7.39-7.55 (m, 6H), 7.23-7.35 (m, 5H), 4.22 (s, 2H). **δ_C** (100 MHz, *d*-$CDCl_3$) δ: 154.9, 153.6, 137.3, 132.4, 131.6, 129.7, 129.3, 129.2, 129.1, 128.9, 128.7, 128.0, 126.9, 119.7, 119.1, 75.1, 33.1. **HRMS** (EI) m/z for $C_{21}H_{14}ClIO$ $[M]^+$: calcd. 443.9778; found, 443.9775.

1.2.15 Synthesis of 5-bromo-7-iodo-2,3-bis(4-methoxyphenyl)benzo[b]furan (7p)

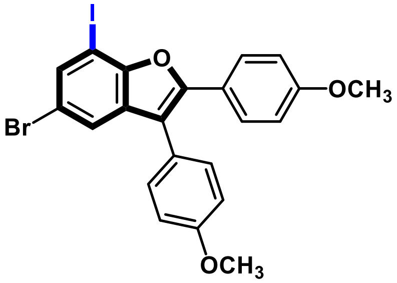

The title compound was prepared using the general procedure and isolated as pale-yellow oil (**71%** yields). **IR** (cast film, cm^{-1}) 2998, 1605, 1592, 1281, 1186, 864, 731. **δ_H** (400 MHz, *d*-$CDCl_3$) δ: 7.74 (d, 1H, *J* = 1.6 Hz), 7.61 (d, 2H, *J* = 8.8 Hz), 7.49 (d, 1 H, *J* = 1.5 Hz), 7.35 (d, 2H, (s, 2H, *J* = 8.56 Hz), 7.00 (d, 2H, *J* = 8.6 Hz), 6.86 (d, 2H, *J* = 9.43 Hz), 3.88 (s, 3H), 3.82 (s, 3H). **δ_C** (100 MHz, *d*-$CDCl_3$) δ: 160.3, 159.6, 153.3, 152.4, 134.7, 132.3, 130.9, 128.7, 128.6, 124.2, 122.5, 116.5, 116.1, 114.8, 114.2, 75.4, 55.5, 55.4. **HRMS** (EI) m/z for $C_{22}H_{16}BrIO_3$ $[M]^+$: calcd. 533.9327; found, 533.9322.

1.2.16 Synthesis of methyl 7-iodo-2,3-bis(4-methoxyphenyl)benzo[b]furan-5-carboxylate (7q)

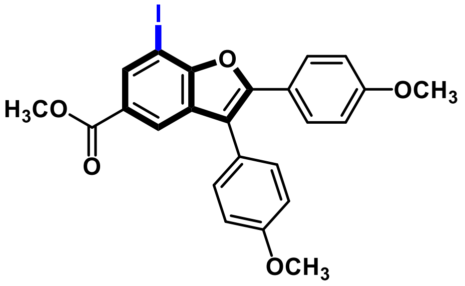

The title compound was prepared using the general procedure and isolated as colorless oil (**83%** yields). **IR** (cast film, cm^{-1}) 3017, 2989, 1756, 1612, 1588, 1260, 1228, 837, 731. **δ_H** (400 MHz, *d*-$CDCl_3$) δ: 8.36 (s, 1H), 8.01 (s, 1H), 7.63 (d, 2H, *J* = 8.44 Hz), 7.39 (d, 2H, *J* = 8.28 Hz), 7.02 (d, 2H, *J* = 8.24 Hz), 6.87 (d, 2H, J^1 = 8.48 Hz),

3.91 (s, 3H), 3.89 (s, 3H), 3.82 (s,3H). **δC** (100 MHz, *d*-$CDCl_3$) δ: 166.3, 160.3, 159.6, 156.7, 152.4, 134.6, 131.0, 130.7, 128.7, 127.0, 124.2, 122.5, 122.2, 116.9, 114.9, 114.2, 74.5, 55.5, 55.4, 52.4. **HRMS** (EI) m/z for $C_{24}H_{19}IO_5$ $[M]^+$: calcd. 514.0277; found, 514.0273.

1.2.17 Synthesis of 7-chloro-2,3-bis(4-methoxyphenyl)benzo[b]furan (7r)

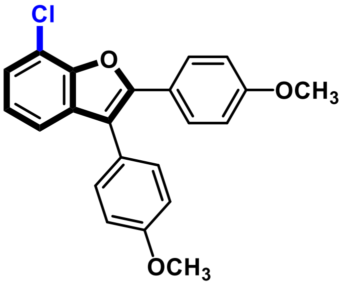

The title compound was prepared using the general procedure and isolated as colorless oil (**77%** yields). **IR** (cast film, cm^{-1}) 3013, 2984, 1609, 1591, 1268, 1153, 855, 694. **δH** (400 MHz, *d*-$CDCl_3$) δ: 7.63 (d, 2H, *J* = 8.64 Hz), 7.39 (d, 2H, *J* = 8.36 Hz), 7.34 (d, 1H, *J* = 7.72 Hz), 7.28 (dd, 1H, J^1 = 7.76 Hz, J^2 = 6.8 Hz), 7.15 (t, 1H, *J* = 7.76 Hz), 7.01 (d, 2H, *J* = 8.4 Hz), 6.86 (d, 2H, *J* = 8.64 Hz), 3.89 (s, 3H), 3.82 (s, 3H). **δC** (100 MHz, *d*-$CDCl_3$) δ: 16.1, 159.4, 151.6, 149.7, 132.5, 131.0, 128.7, 124.8, 124.4, 123.8, 123.0, 118.4, 116.6, 116.2, 114.7, 114.1, 55.5, 55.4. **HRMS** (EI) m/z for $C_{22}H_{17}ClO_3$ $[M]^+$: calcd. 364.0866; found, 364.0865.

1.2.18 Synthesis of 7-chloro-2,3-diphenylbenzo[b]furan (7s)

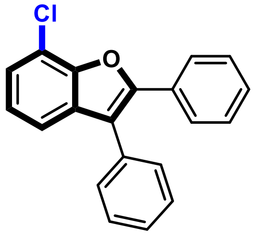

The title compound was prepared using the general procedure for palladium-catalyzed regioselective domino α-arylation/intramolecular O-arylation reaction and isolated as white solid (**71%** yields). The spectroscopic data for this compound are matched the previous report by *Chem. Commun*., **2013**, *49*, 6611-6613.

1.2.19 Synthesis of 7-bromo-2,3-bis(4-methoxyphenyl)benzo[b]furan (7t)

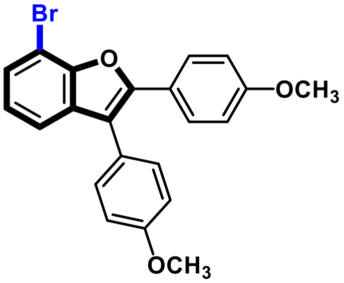

The title compound was prepared using the general procedure and isolated as pale-yellow oil (**73%** yields). **IR** (cast film, cm^{-1}) 3005, 2998, 1601, 1576, 1291, 1183, 911, 844. **δ_H** (400 MHz, *d*-$CDCl_3$) δ: 7.63 (d, 2H, *J* = 8.88 Hz), 7.37-7.45 (m, 4H), 7.09 (t, 1H, *J* = 7.76 Hz), 7.00 (d, 2H, *J* = 8.68 Hz), 6.86 (d, 2H, *J* = 8.88 Hz), 3.89 (s, 3H), 3.82 (s, 3H). **δ_C** (100 MHz, *d*-$CDCl_3$) δ: 160.1, 159.4, 151.5, 151.1, 132.1, 131.0, 128.7, 127.2, 124.8, 124.2, 123.0, 119.0, 116.3, 114.7, 114.1, 103.9, 55.5, 55.4. **HRMS** (EI) m/z for $C_{22}H_{17}BrO_3$ $[M]^+$: calcd. 408.0361; found, 408.0355.

1.2.20 Synthesis of 7-bromo-2,3-diphenylbenzo[b]furan (7u)

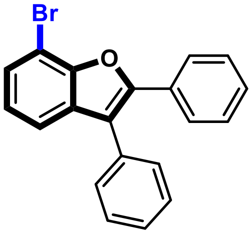

The title compound was prepared using the general procedure for palladium-catalyzed regioselective domino α-arylation/intramolecular O-arylation reaction and isolated as white solid (**72%** yields). The spectroscopic data for this compound are matched the previous report by *Chem. Commun*., **2013**, *49*, 6611-6613.

1.2.21 Synthesis of 1-(5-chloro-2,3-diiodophenyl)-1-(4-methoxyphenyl)propan-2-one

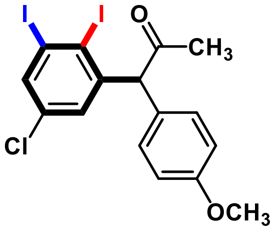

The title compound was prepared using the general procedure and isolated as pale-yellow oil (**53%** yields). **IR** (cast film, cm^{-1}) 3012, 2985, 1715, 1625, 1601, 1253, 1107, 837, 721. **δ_H** (400 MHz, *d*-$CDCl_3$) δ: 7.78 (d, 1H, *J* = 2.2 Hz), 7.12 (d, 2H, *J* = 8.56 Hz), 6.94 (d, 2H, *J* = 8.6 Hz), 6.78 (d, 1H, *J* = 2.2 Hz), 5.55 (s, 1H), 3.83 (s, 3H), 2.30 (s, 3H). **δ_C** (100 MHz, *d*-$CDCl_3$) δ: 205.2, 159.6, 146.8, 137.9, 135.3, 130.8, 129.6, 128.2, 115.0,

113.4, 110.8, 71.9, 55.5, 30.9. **HRMS** (ESI) m/z for $C_{16}H_{14}ClI_2O_2$ $[M+H]^+$: calcd. 526.8772; found, 526.8765.

1.3 NMR Spectra for New Compounds

1.3.1 ^{1}H-NMR of 7-iodo-2,3-bis(4-methoxyphenyl)benzofuran (7a) in d-$CDCl_3$ at 25 ^{o}C.

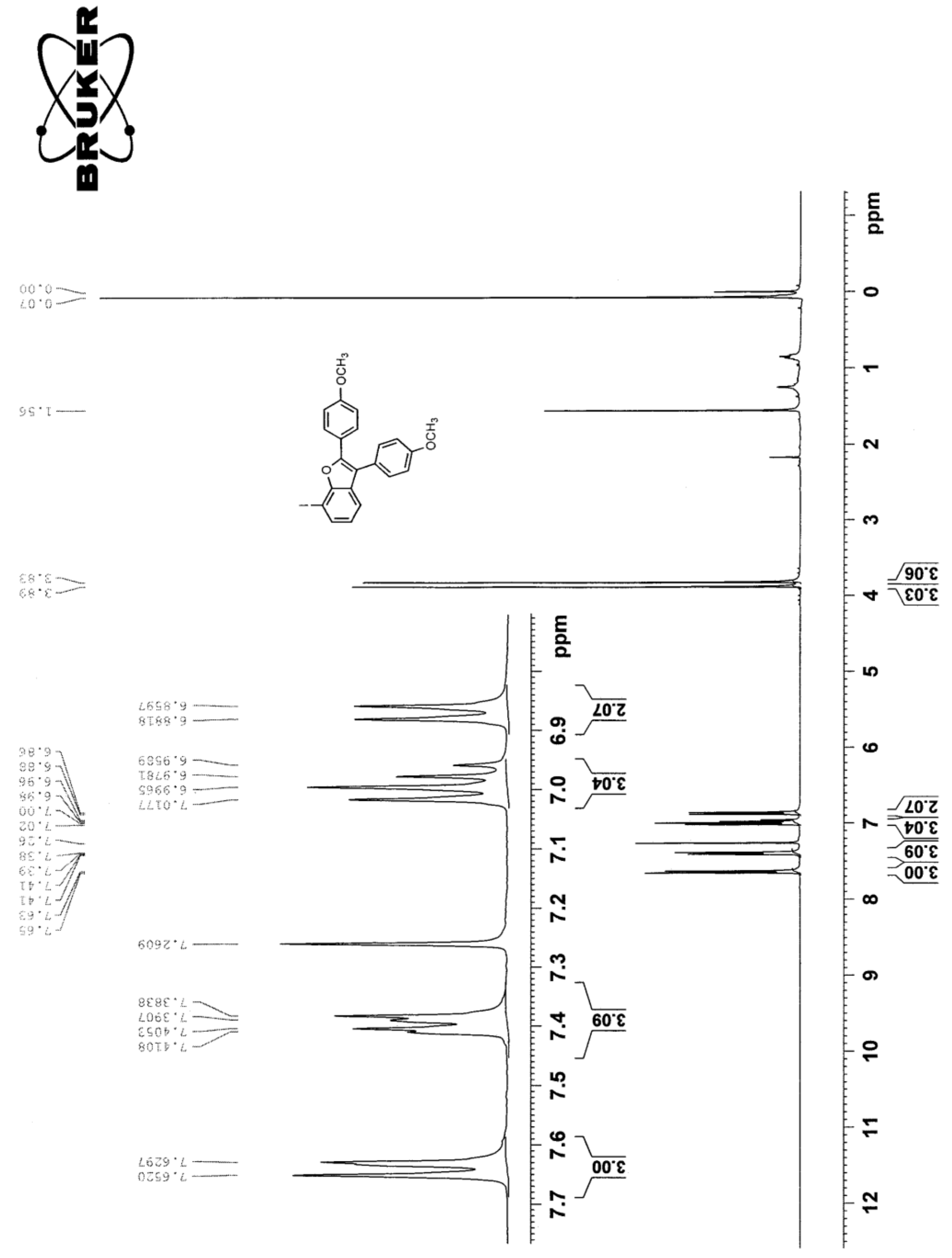

1.3.2 ^{13}C-NMR of 7-iodo-2,3-bis(4-methoxyphenyl)benzofuran (7a) in d-$CDCl_3$ at 25 ^{o}C.

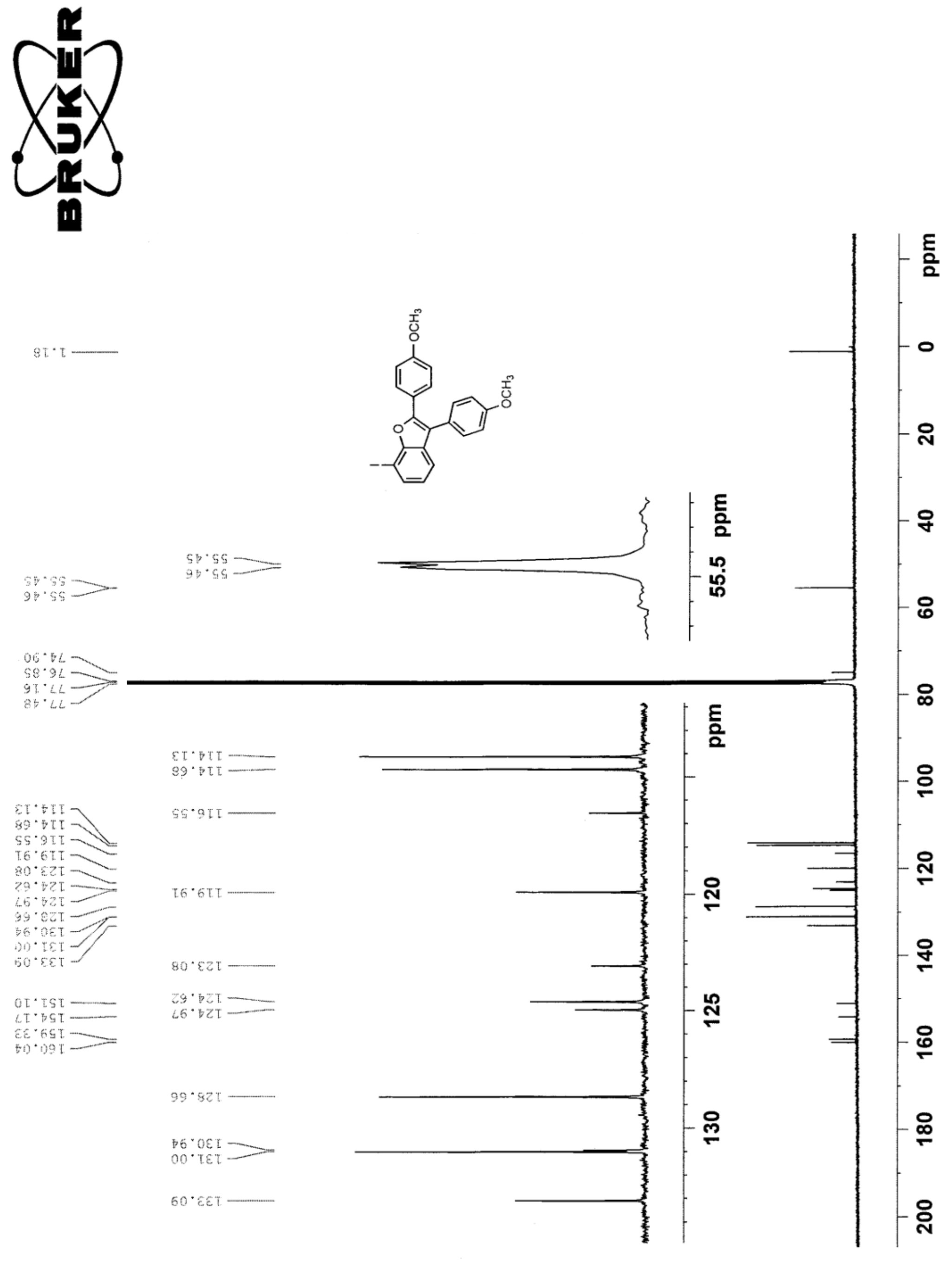

1.3.3 ^{1}H-NMR of 7-iodo-2,3-diphenylbenzofuran (7b) in d-$CDCl_3$ at 25 ^oC.

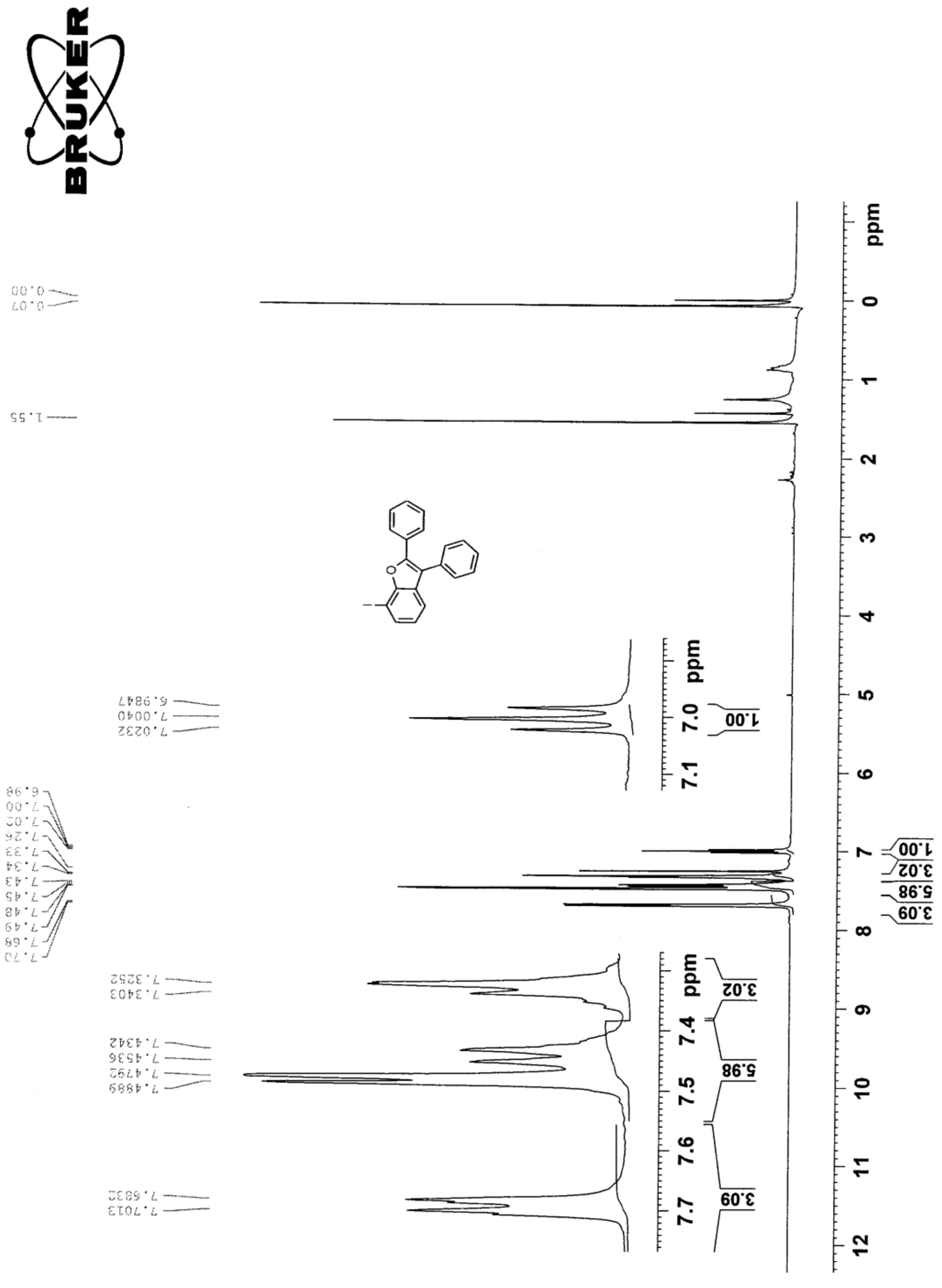

1.3.4 ^{13}C-NMR of 7-iodo-2,3-diphenylbenzofuran (7b) in d-$CDCl_3$ at 25 °C.

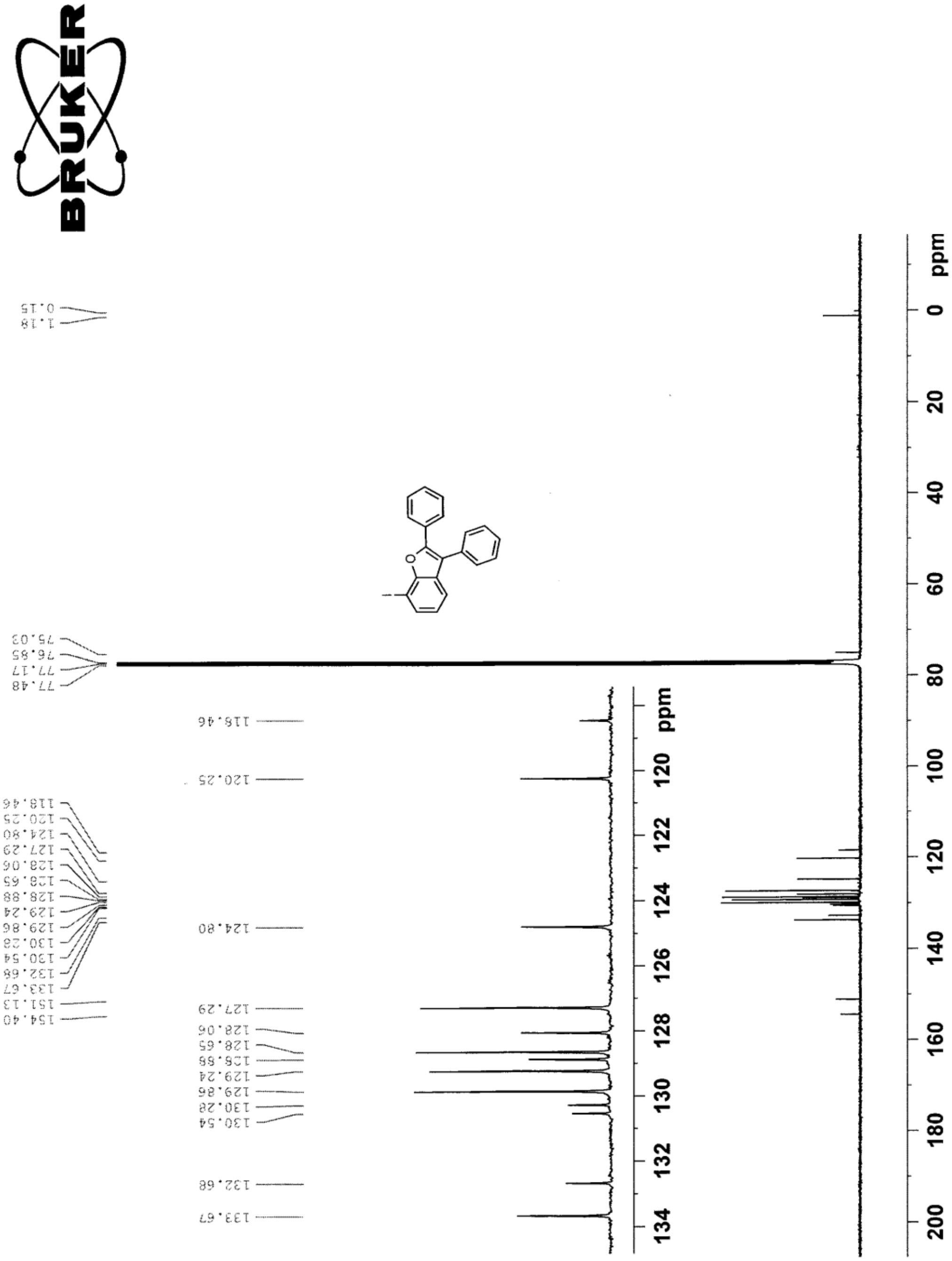

R. M. Al-Zoubi, W. K. Al-Jammal, M.J. Ferguson and G. K. Murphy

1.3.5 ¹H-NMR of 2-(4-chlorophenyl)-7-iodo-3-phenylbenzofuran (7c) in d-$CDCl_3$ at 25 °C.

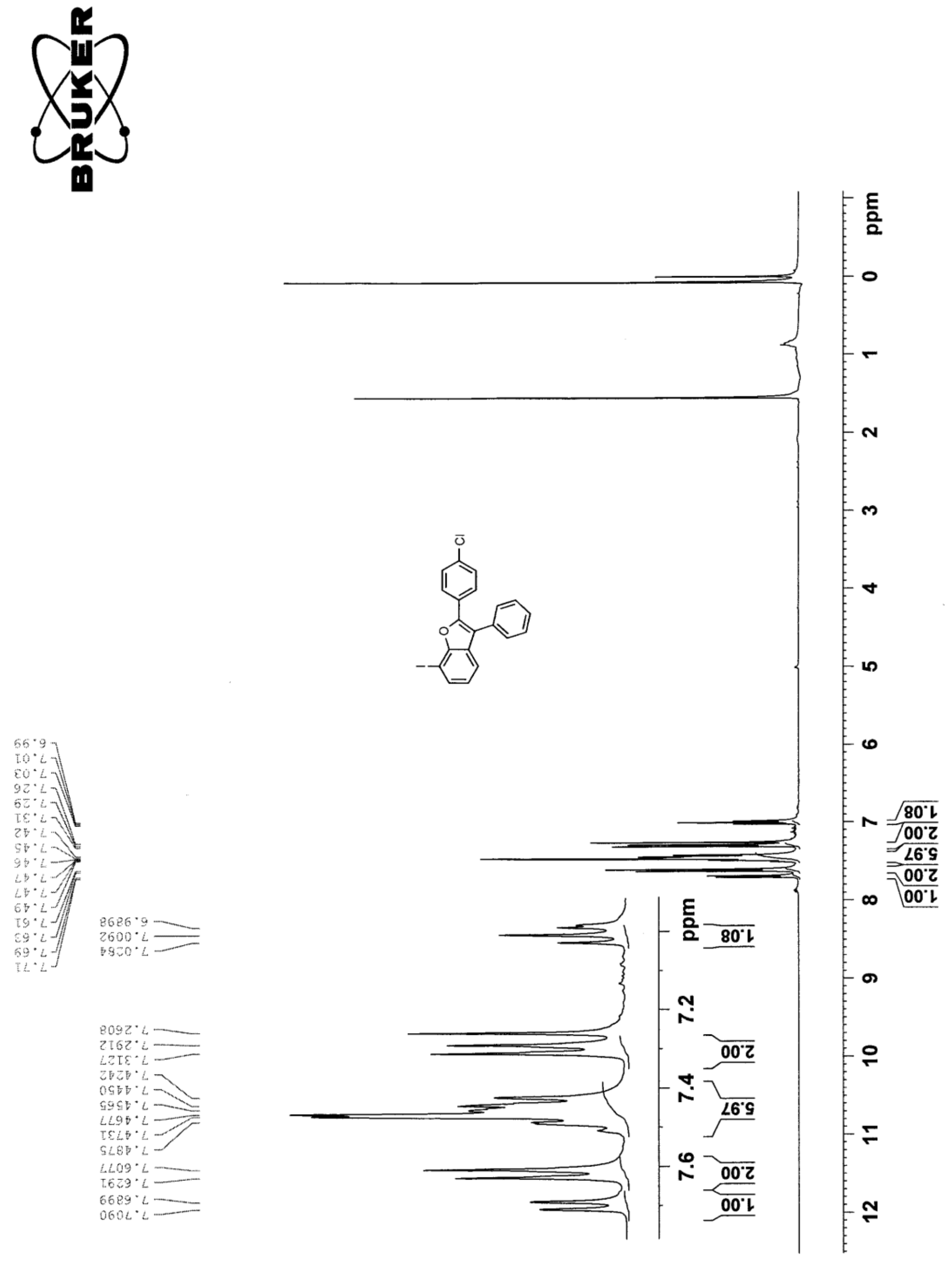

1.3.6 ^{13}C-NMR of 2-(4-chlorophenyl)-7-iodo-3-phenylbenzofuran (7c) in d-CDCl$_3$ at 25 ^{o}C.

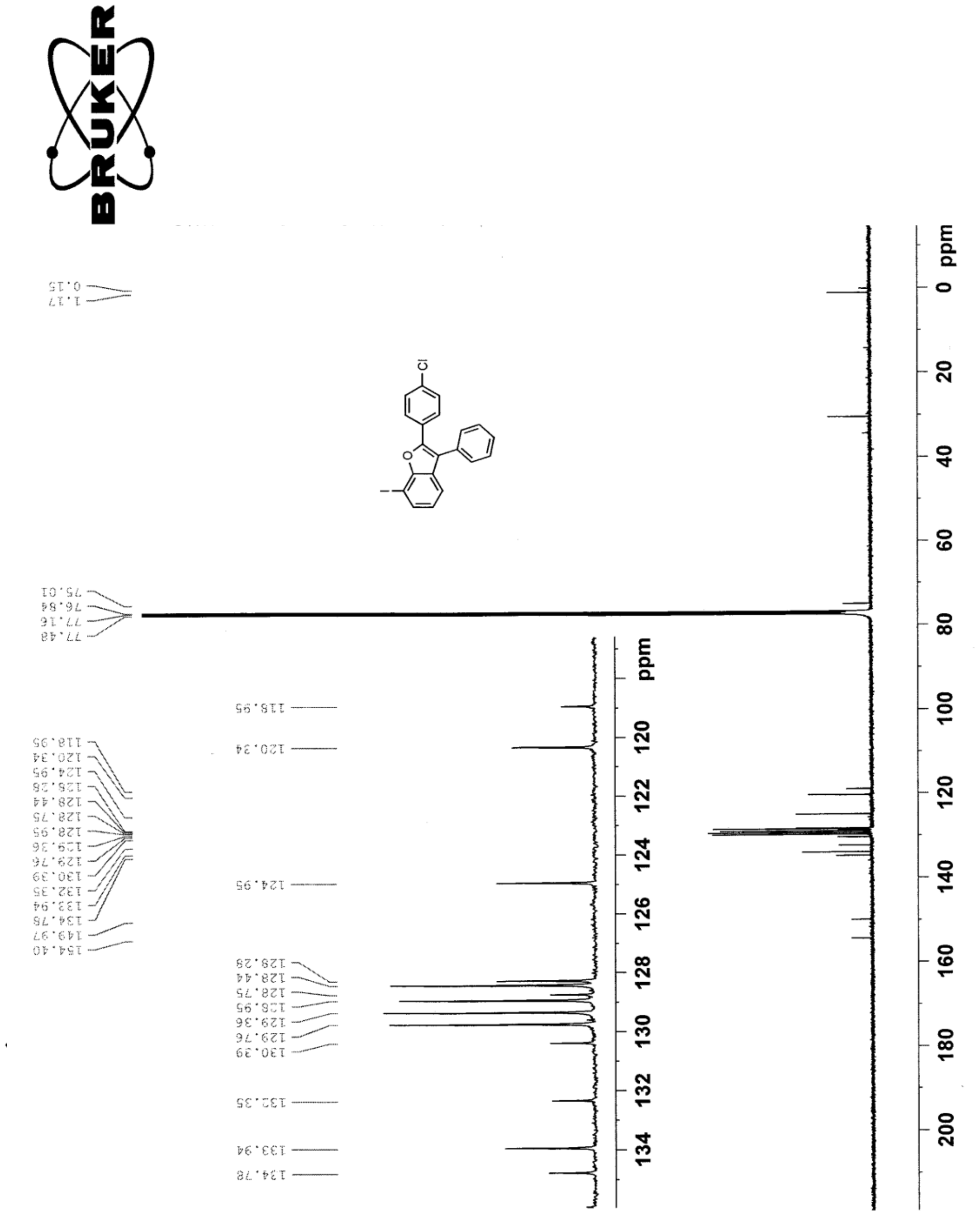

1.3.7 ^{1}H-NMR of 7-iodo-3-(4-methoxyphenyl)-2-methylbenzofuran (7d) in d-$CDCl_3$ at 25 ^{o}C

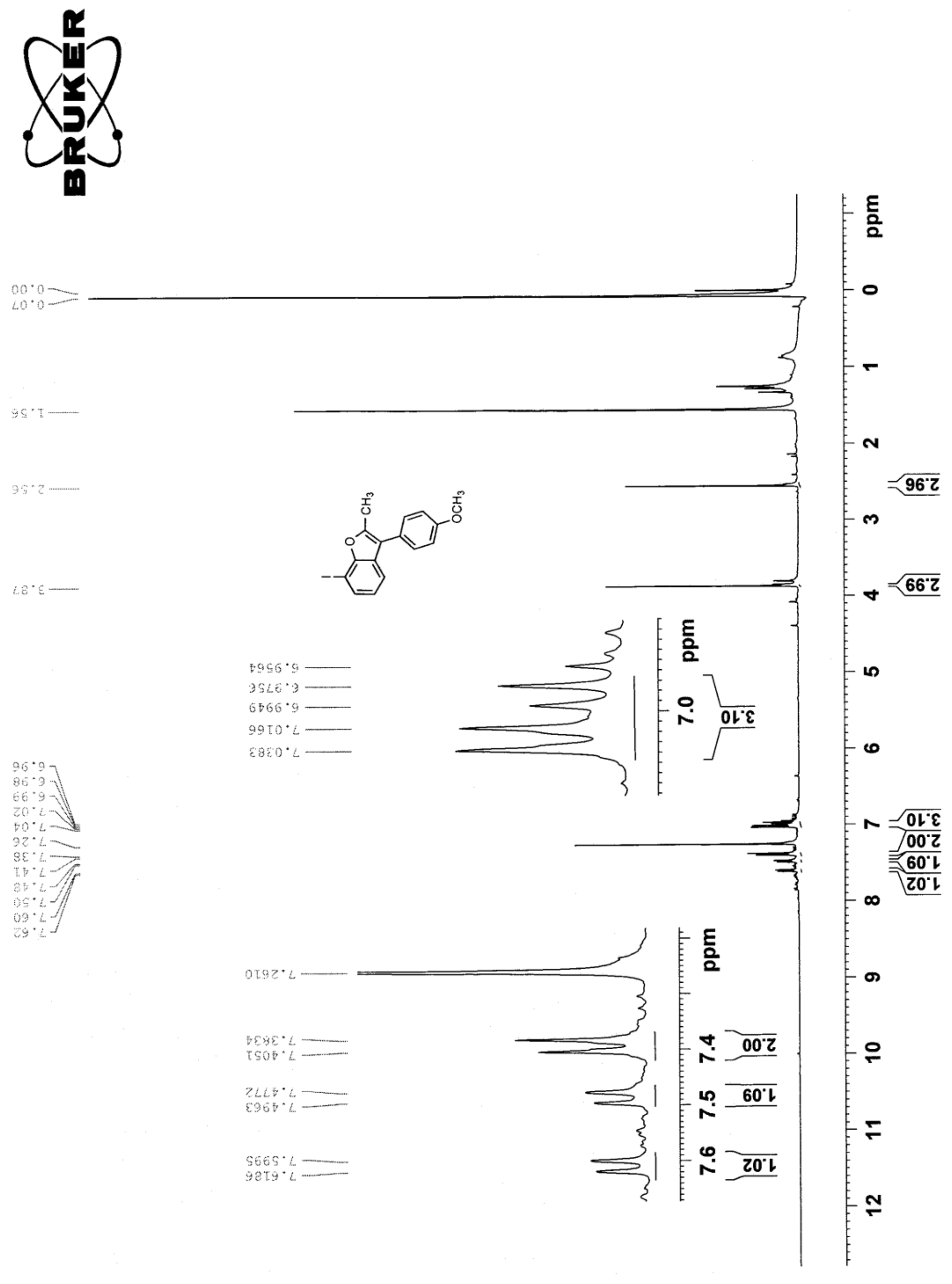

1.3.8 ^{13}C-NMR of 7-iodo-3-(4-methoxyphenyl)-2-methylbenzofuran (7d) in d-$CDCl_3$ at 25 ^{o}C.

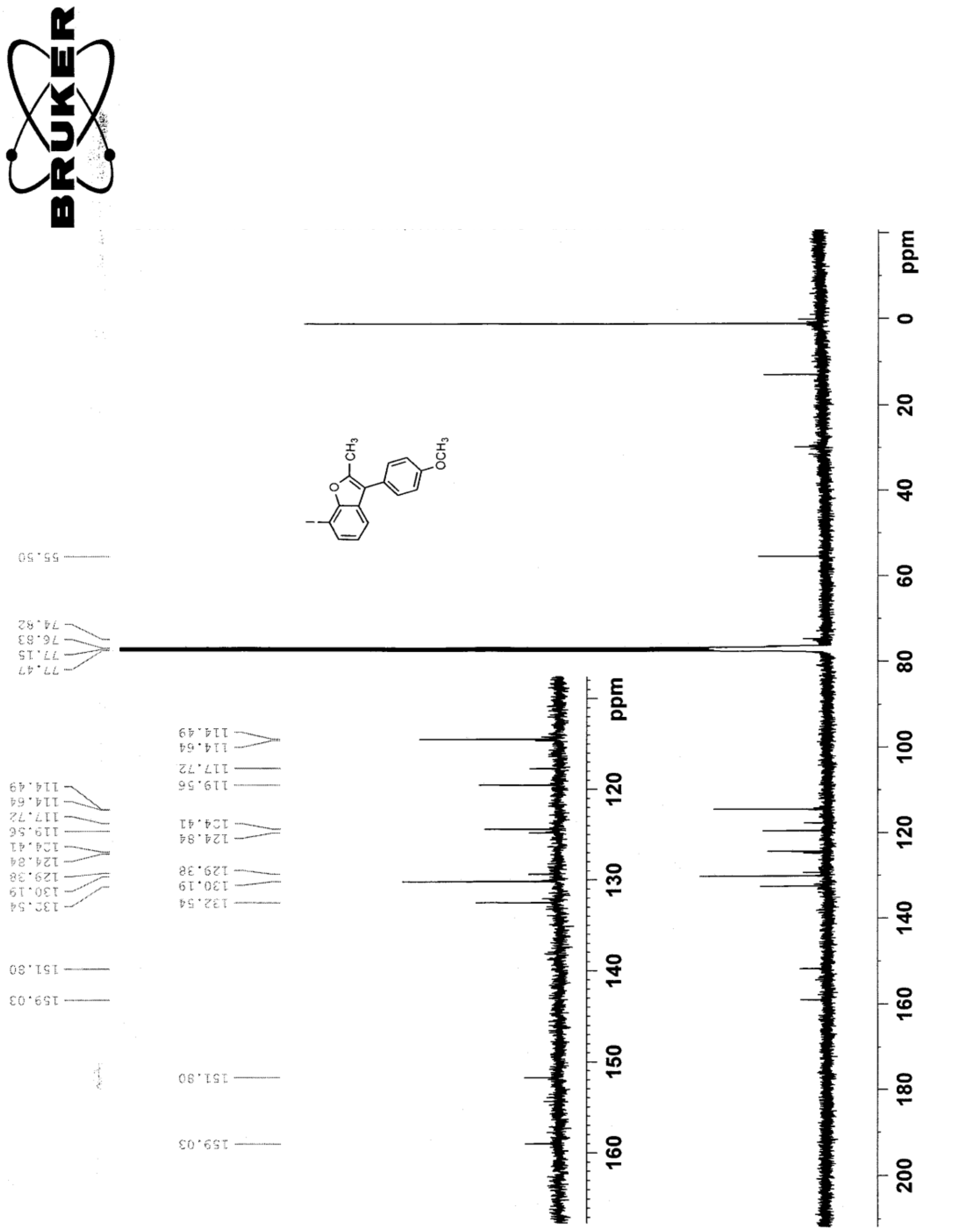

R. M. Al-Zoubi, W. K. Al-Jammal, M.J. Ferguson and G. K. Murphy

1.3.9 ^{1}H-NMR of 7-iodo-2,3-bis(4-methoxyphenyl)-5-methylbenzofuran (7e) in d-CDCl$_3$ at 25 ^{o}C

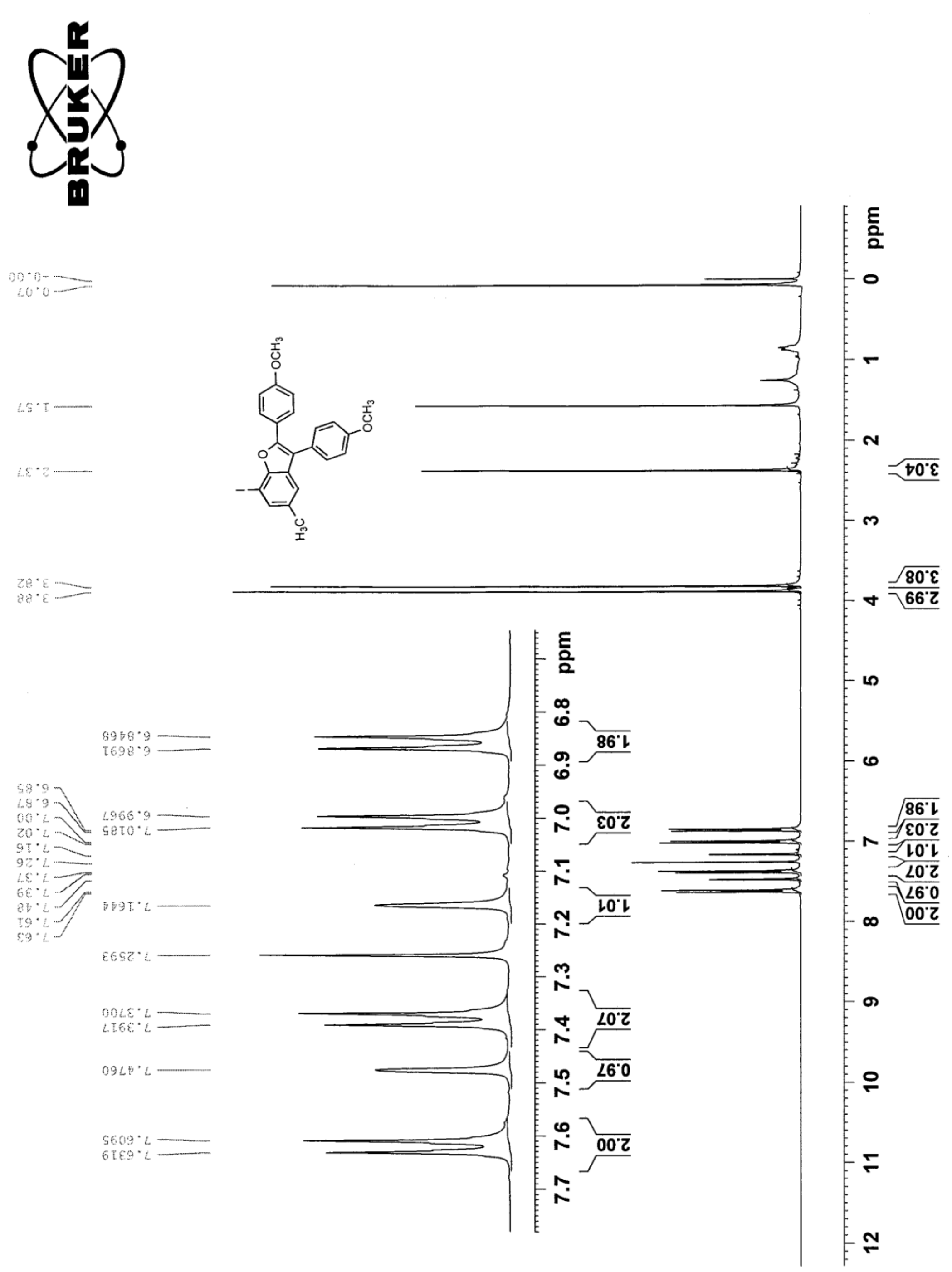

1.3.10 ^{13}C-NMR of 7-iodo-2,3-bis(4-methoxyphenyl)-5-methylbenzofuran (7e) in d-$CDCl_3$ at 25 °C.

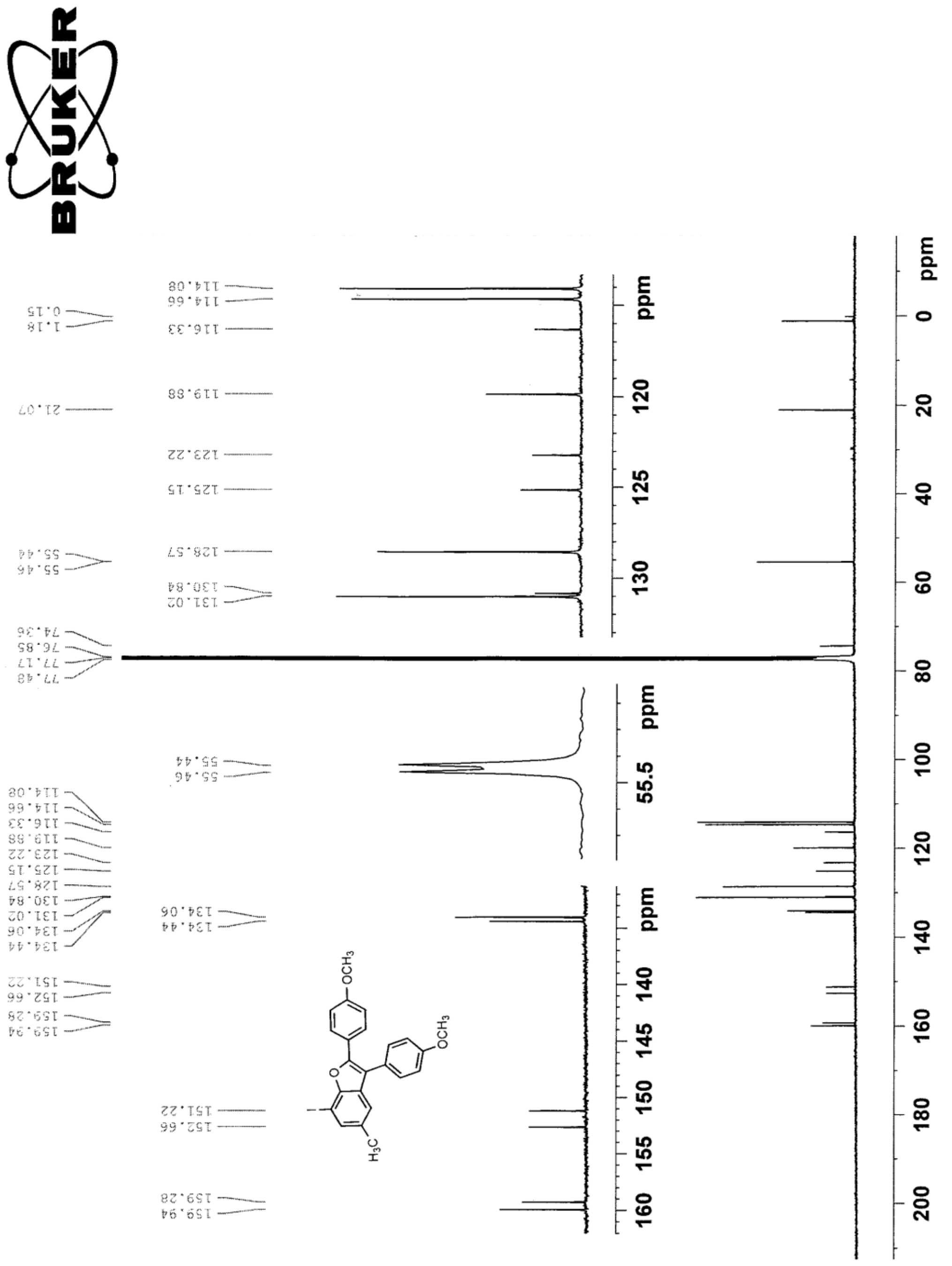

R. M. Al-Zoubi, W. K. Al-Jammal, M.J. Ferguson and G. K. Murphy

1.3.11 ^{1}H-NMR of 7-iodo-5-methyl-2,3-diphenylbenzofuran (7f) in d-$CDCl_3$ at 25 ^{o}C.

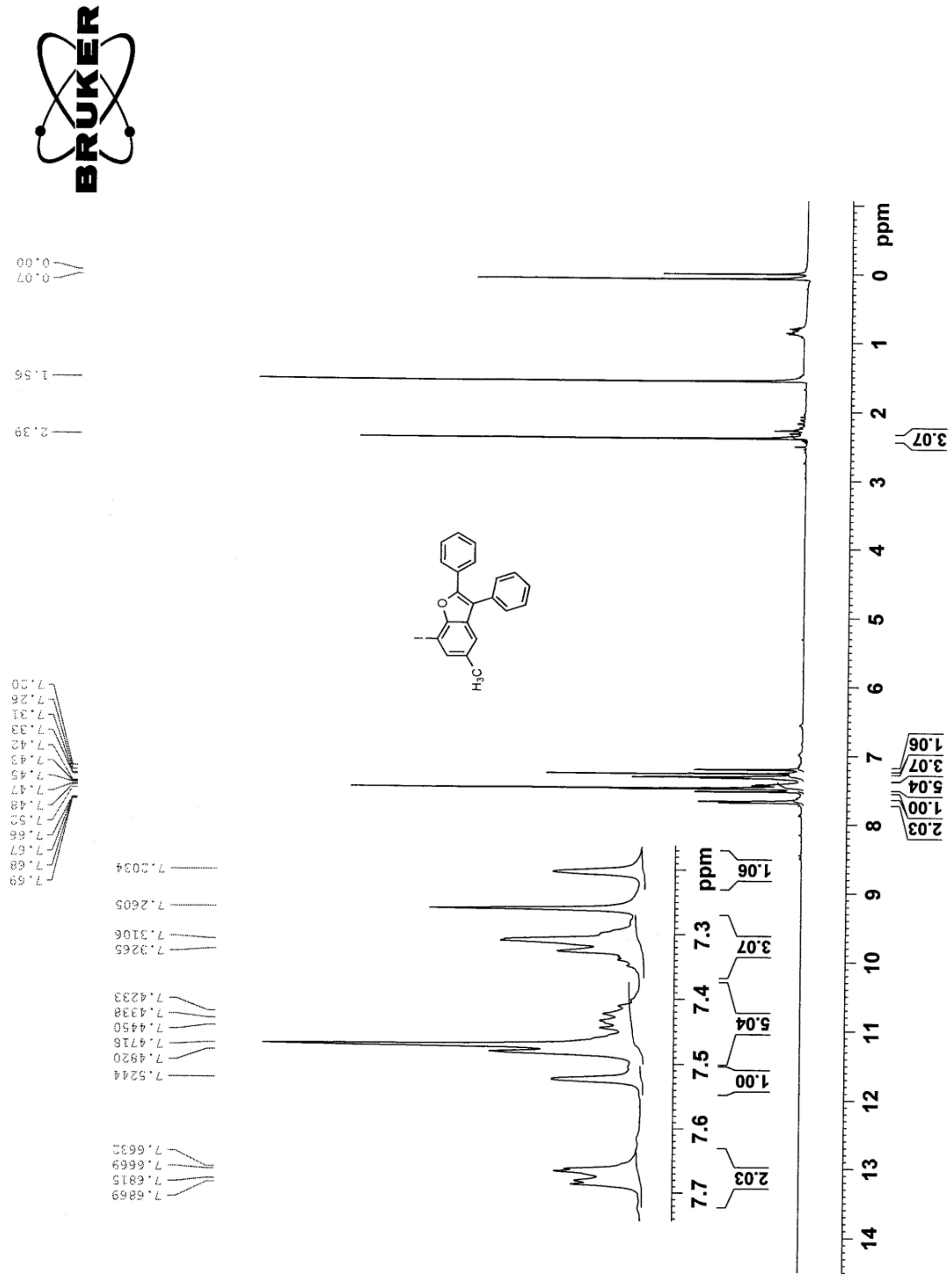

1.3.12 ^{13}C-NMR of 7-iodo-5-methyl-2,3-diphenylbenzofuran (7f) in d-$CDCl_3$ at 25 ^{o}C.

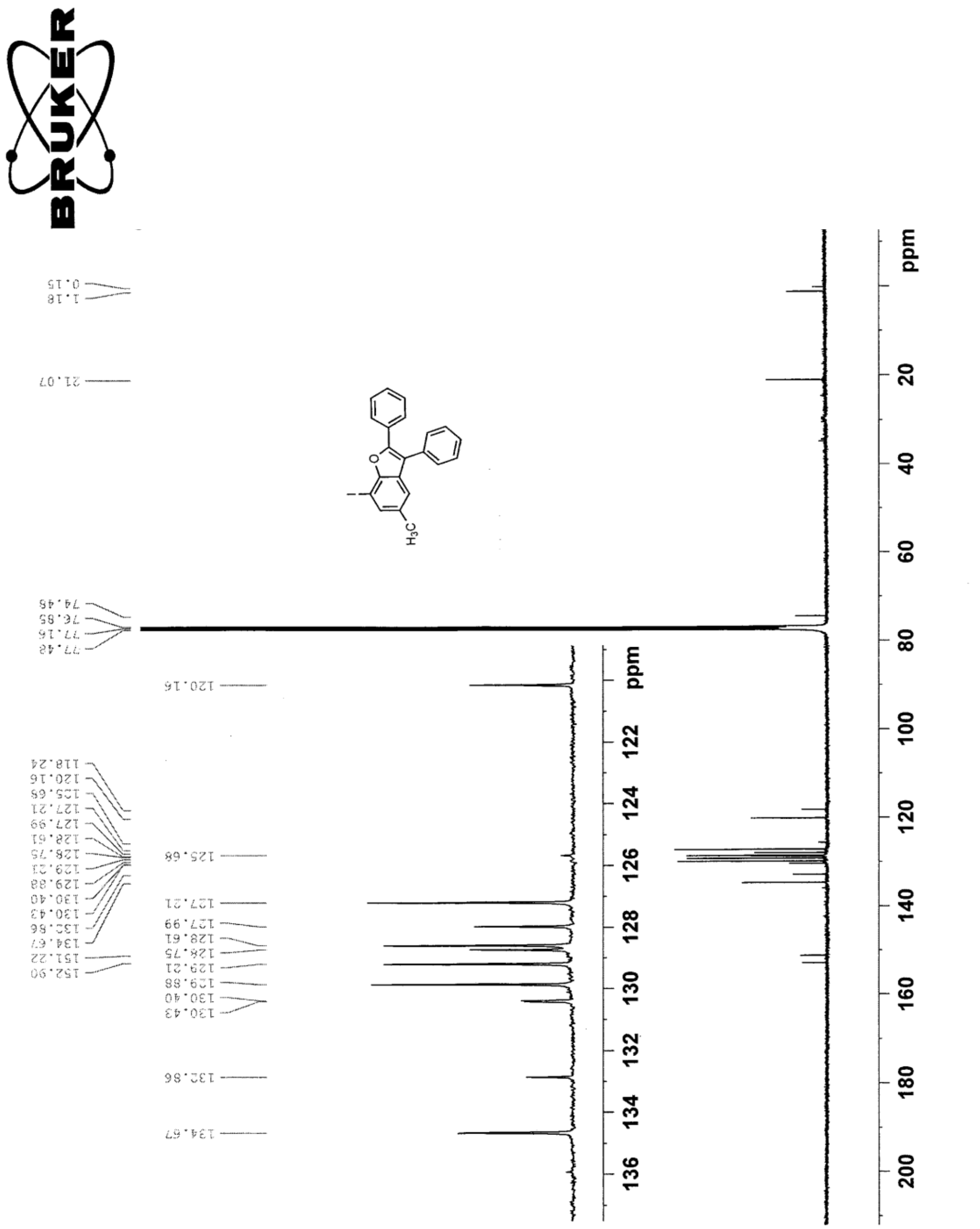

1.3.13 ^{1}H-NMR of 2-(4-chlorophenyl)-7-iodo-5-methyl-3-phenylbenzofuran (7g) in d-$CDCl_3$ at 25 ^{o}C.

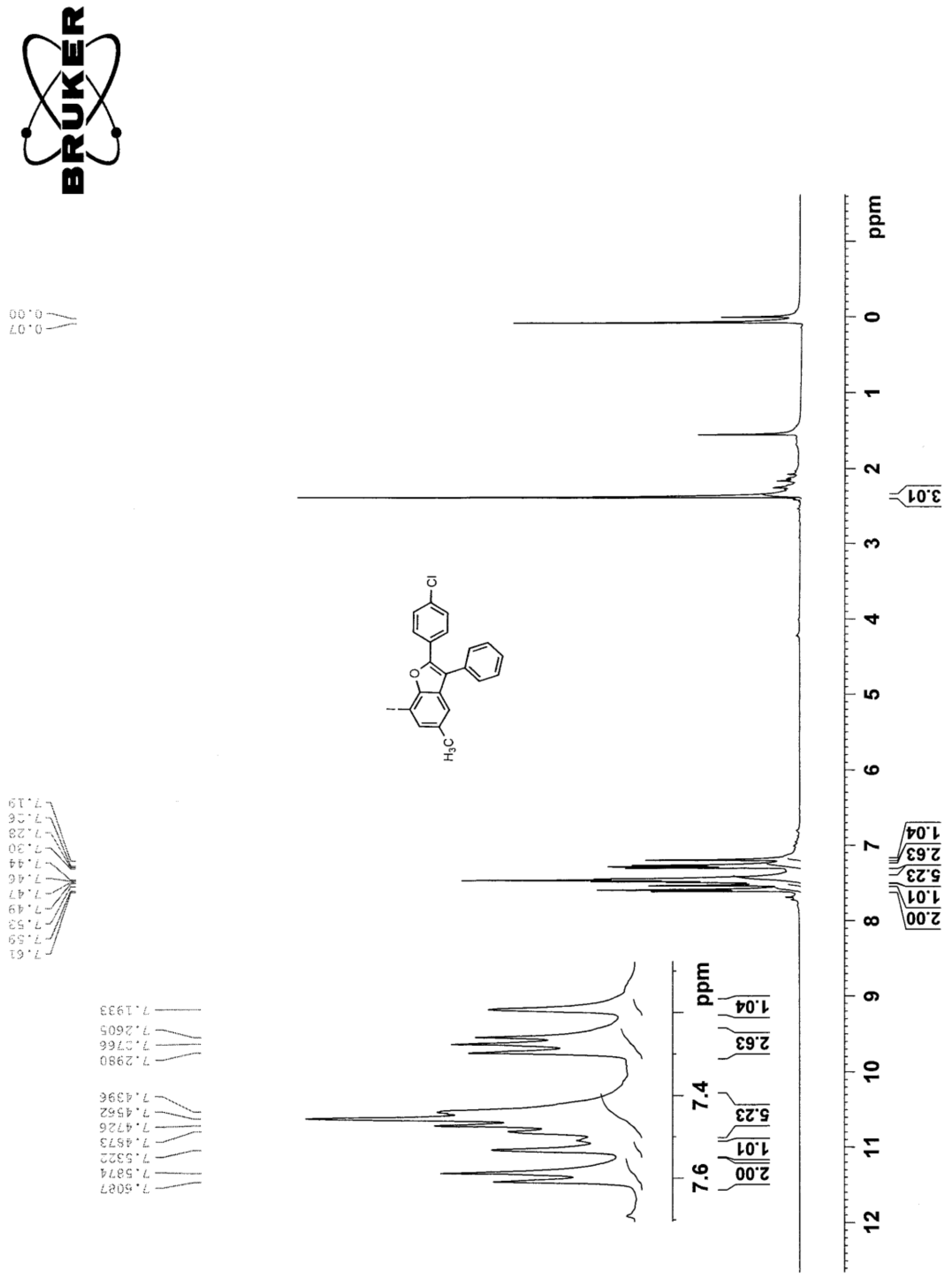

1.3.14 ^{13}C-NMR of 2-(4-chlorophenyl)-7-iodo-5-methyl-3-phenylbenzofuran (7g) in d-$CDCl_3$ at 25 °C.

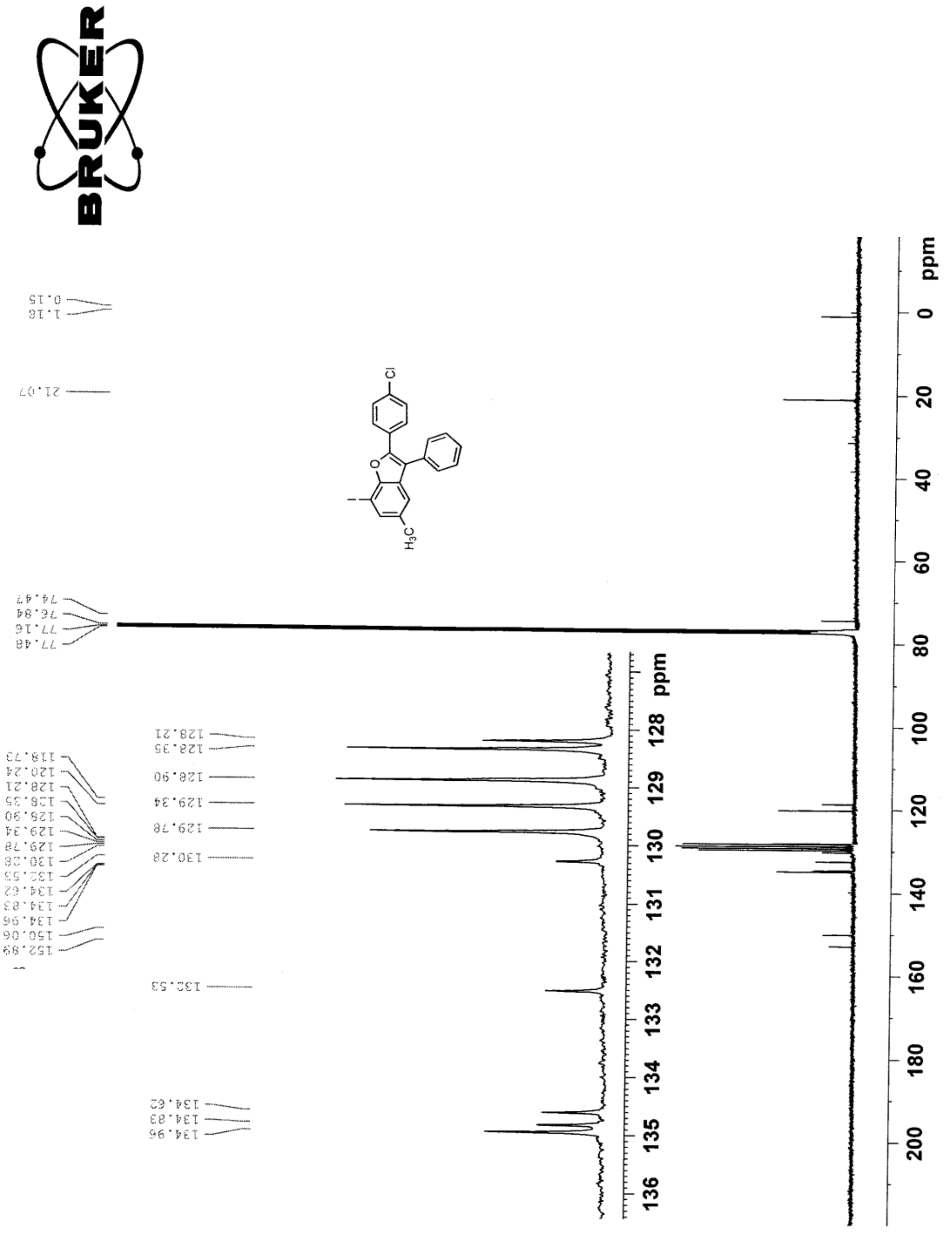

R. M. Al-Zoubi, W. K. Al-Jammal, M.J. Ferguson and G. K. Murphy

1.3.15 ^{1}H-NMR of 7-iodo-5-methoxy-2,3-bis(4-methoxyphenyl)benzofuran (7h) in d-$CDCl_3$ at 25 ^{o}C.

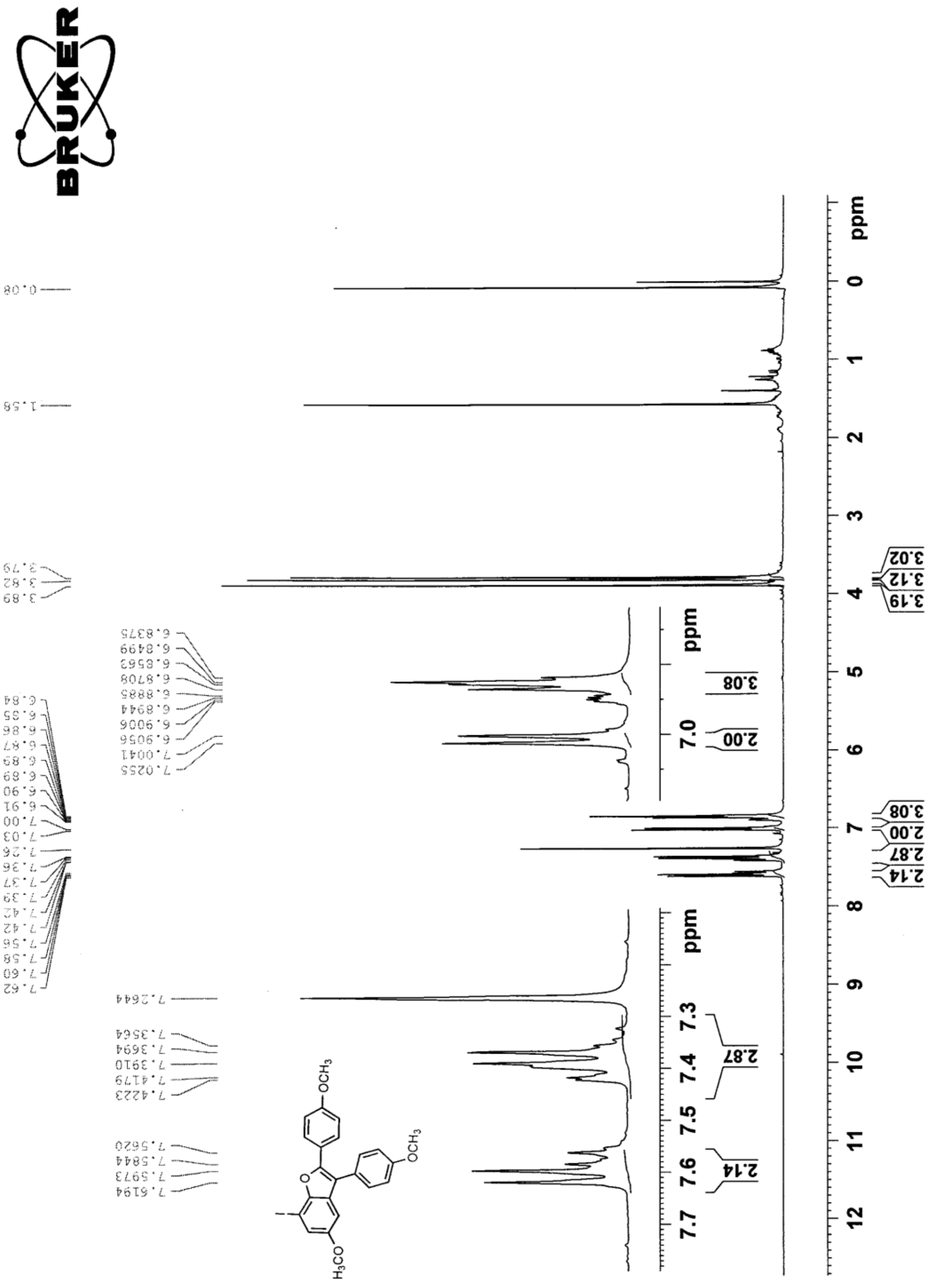

1.3.16 ^{13}C-NMR of 7-iodo-5-methoxy-2,3-bis(4-methoxyphenyl)benzofuran (7h) in d-CDCl$_3$ at 25 ^{o}C.

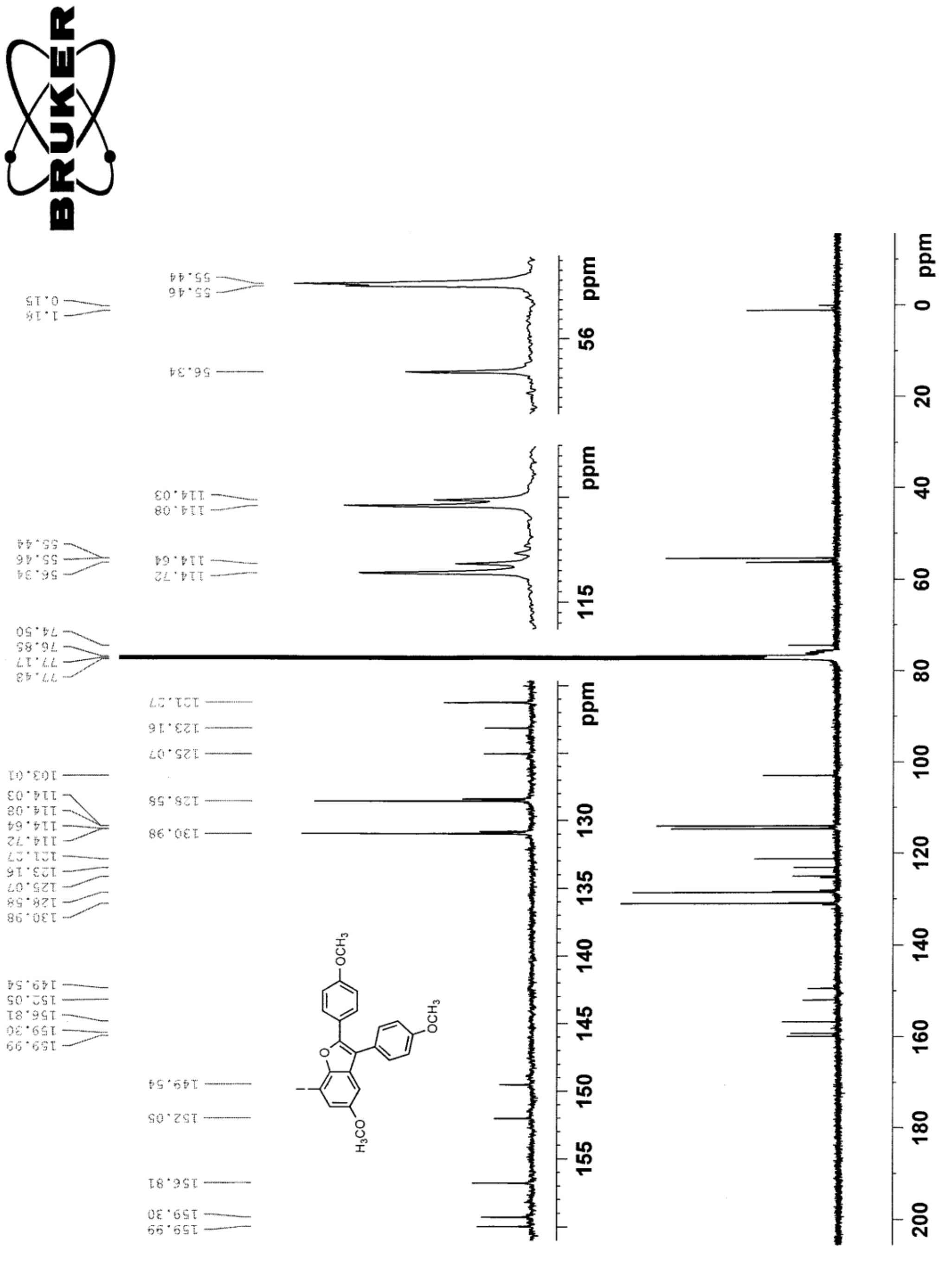

1.3.17 ^{1}H-NMR of 7-iodo-5-methoxy-2,3-diphenylbenzofuran (7i) in d-$CDCl_3$ at 25 ^{o}C.

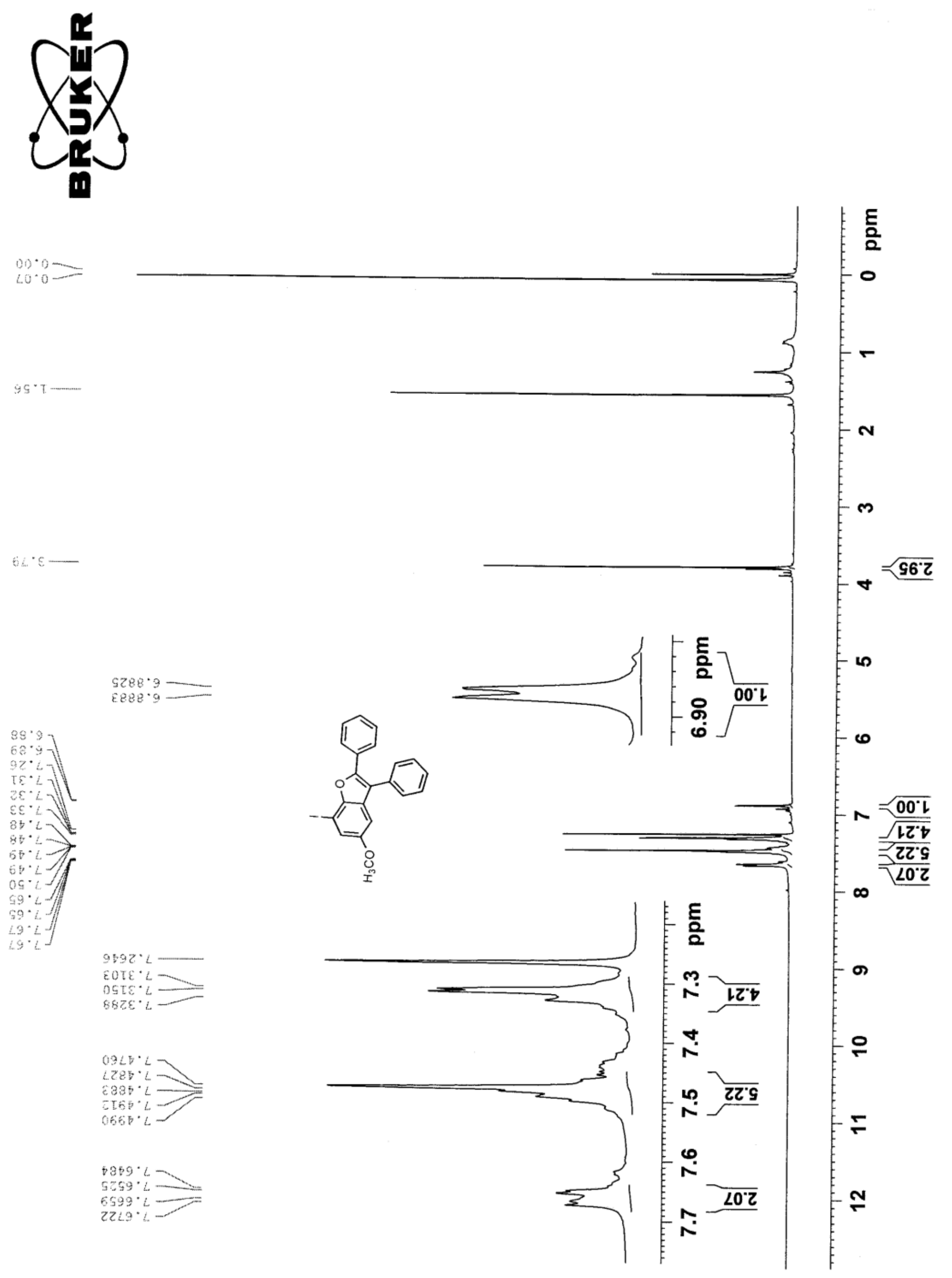

1.3.18 ^{13}C-NMR of 7-iodo-5-methoxy-2,3-diphenylbenzofuran (7i) in d-$CDCl_3$ at 25 °C.

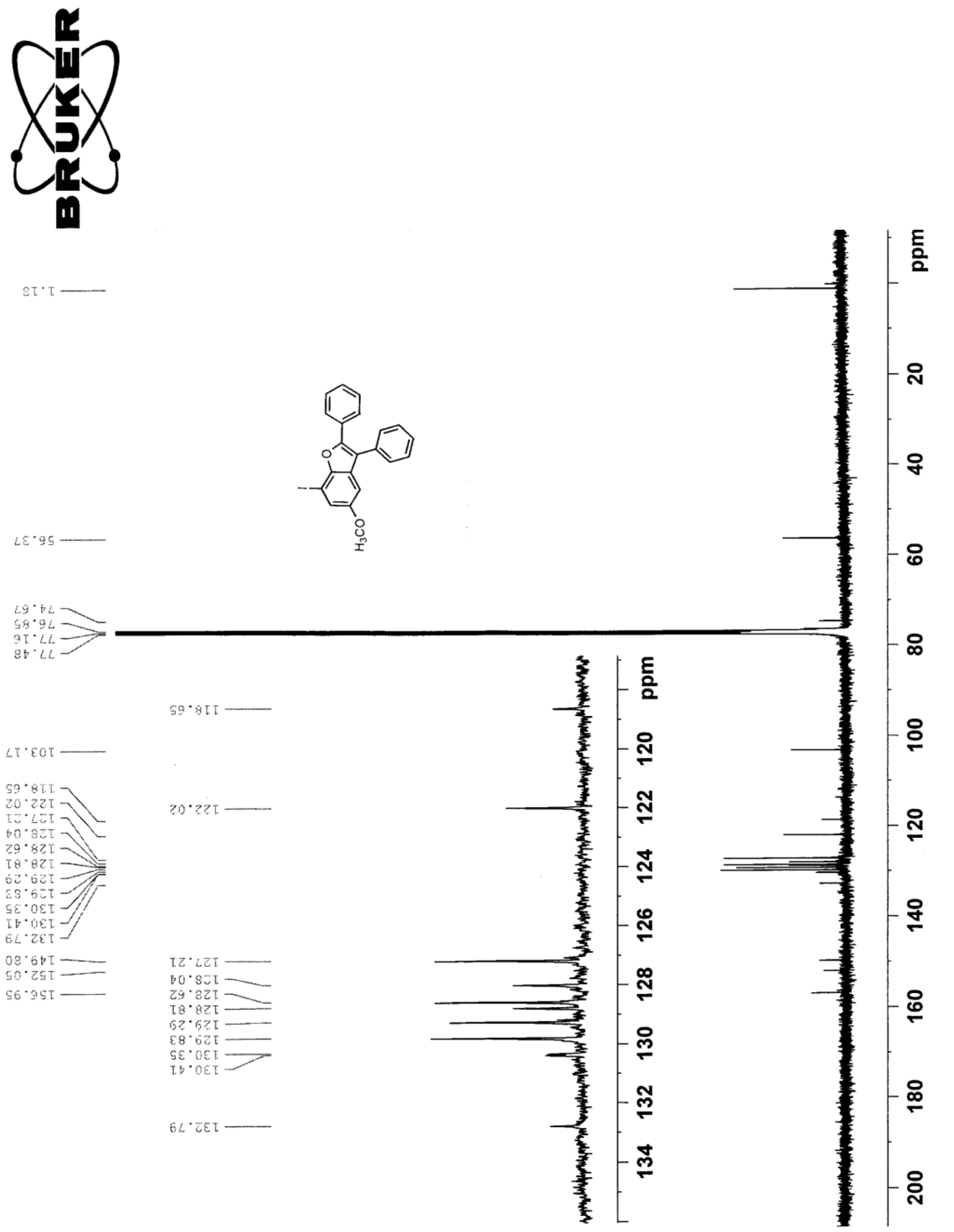

1.3.19 ¹H-NMR of 7-iodo-5-methoxy-3-(4-methoxyphenyl)-2-methylbenzofuran (7j) in d-$CDCl_3$ at 25 °C.

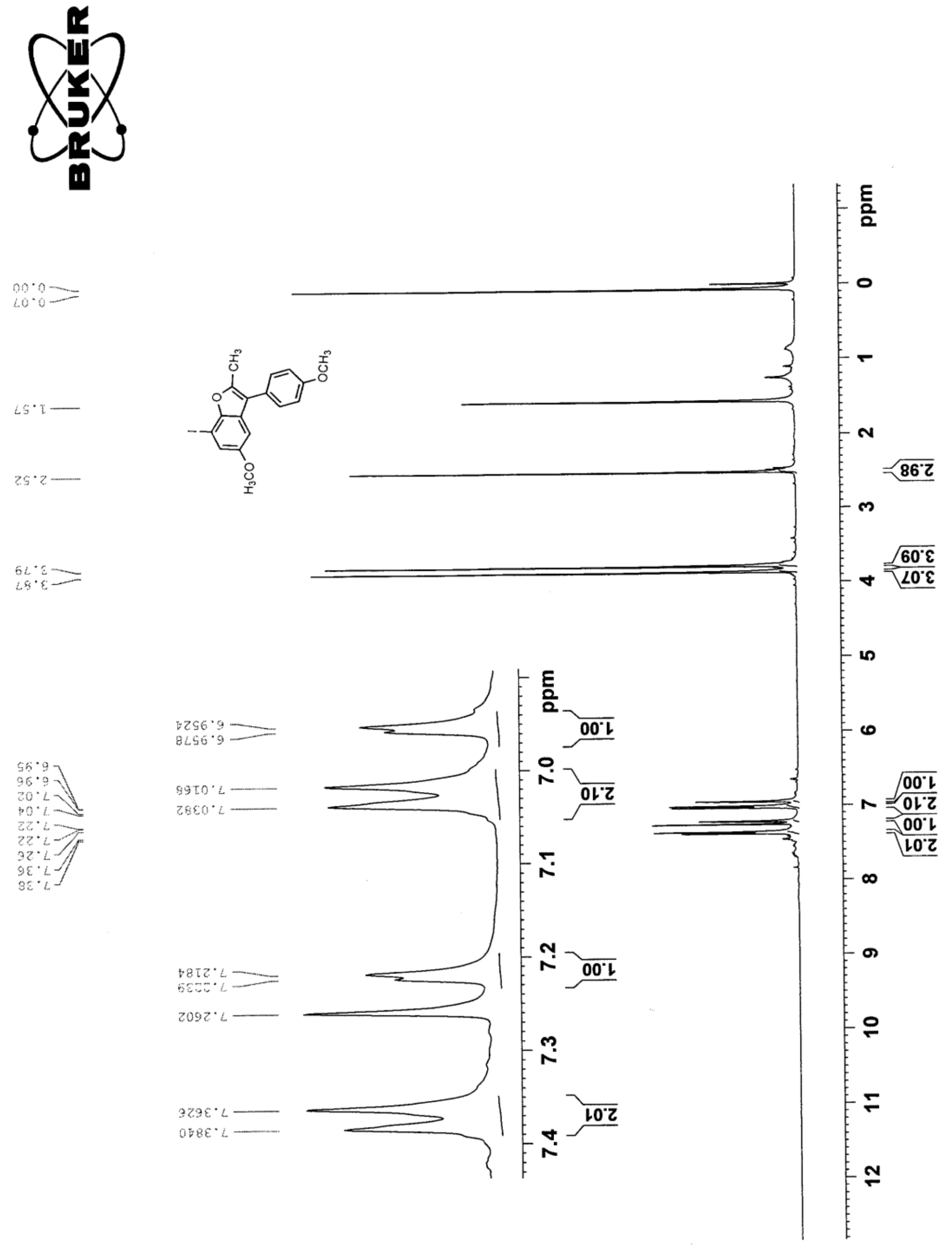

1.3.20 ^{13}C-NMR of 7-iodo-5-methoxy-3-(4-methoxyphenyl)-2-methylbenzofuran (7j) in d-$CDCl_3$ at 25 °C.

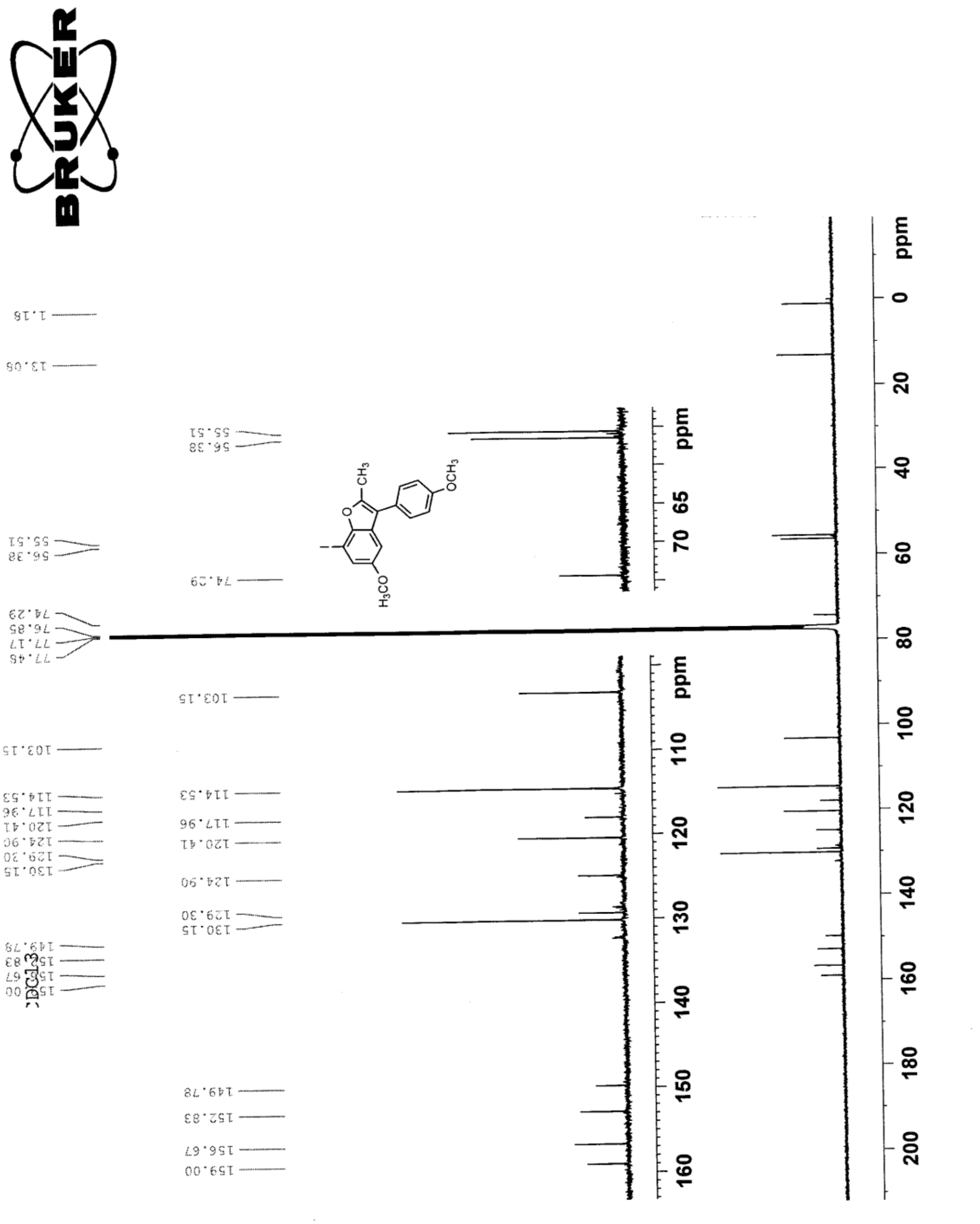

R. M. Al-Zoubi, W. K. Al-Jammal, M.J. Ferguson and G. K. Murphy

1.3.21 ^{1}H-NMR of 5-fluoro-7-iodo-2,3-bis(4-methoxyphenyl)benzofuran nzofuran (7k) in d-CDCl$_3$ at 25 ^{o}C

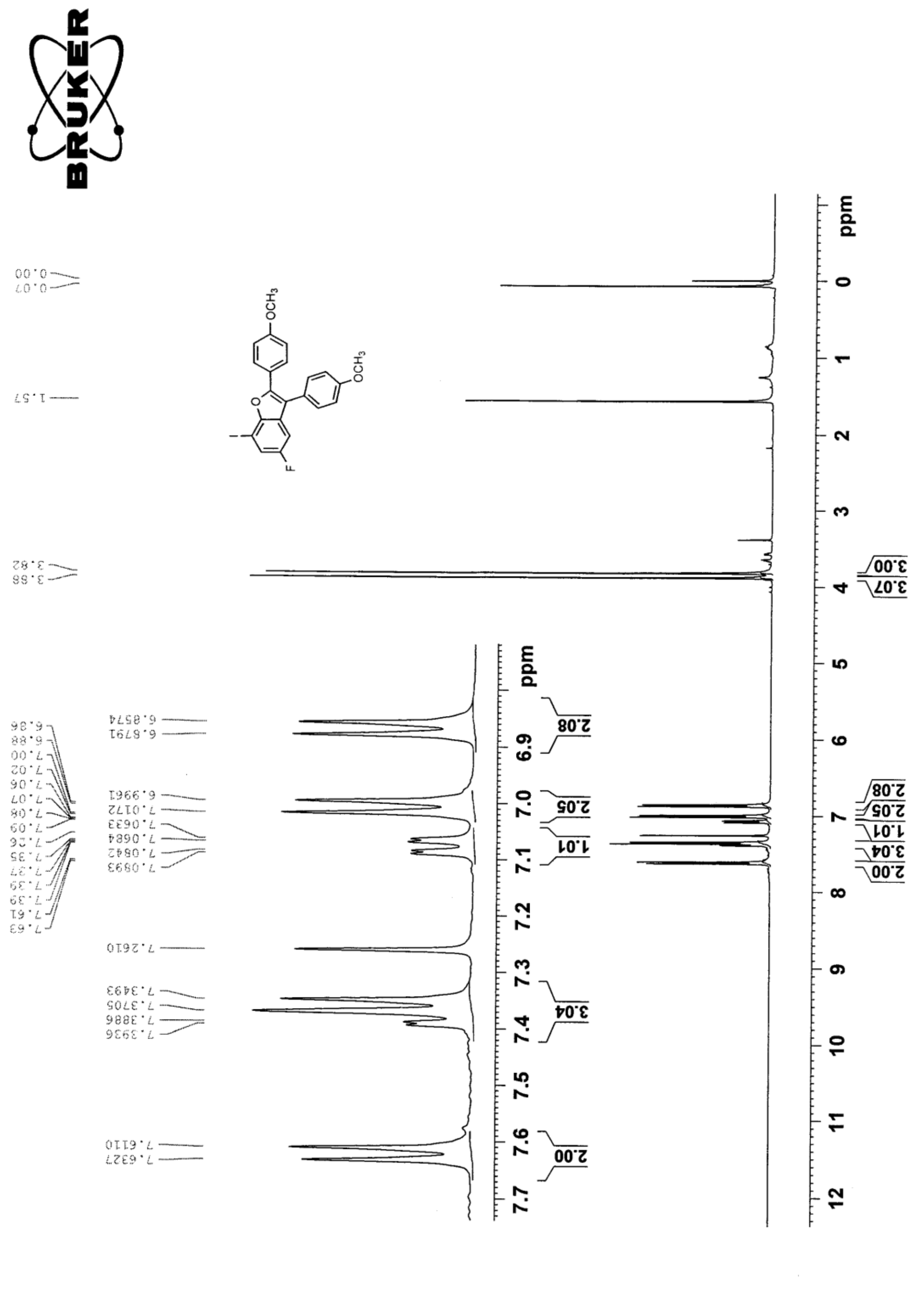

1.3.22 ^{13}C-NMR of 5-fluoro-7-iodo-2,3-bis(4-methoxyphenyl)benzofuran (7k) in d-$CDCl_3$ at 25 ^{o}C.

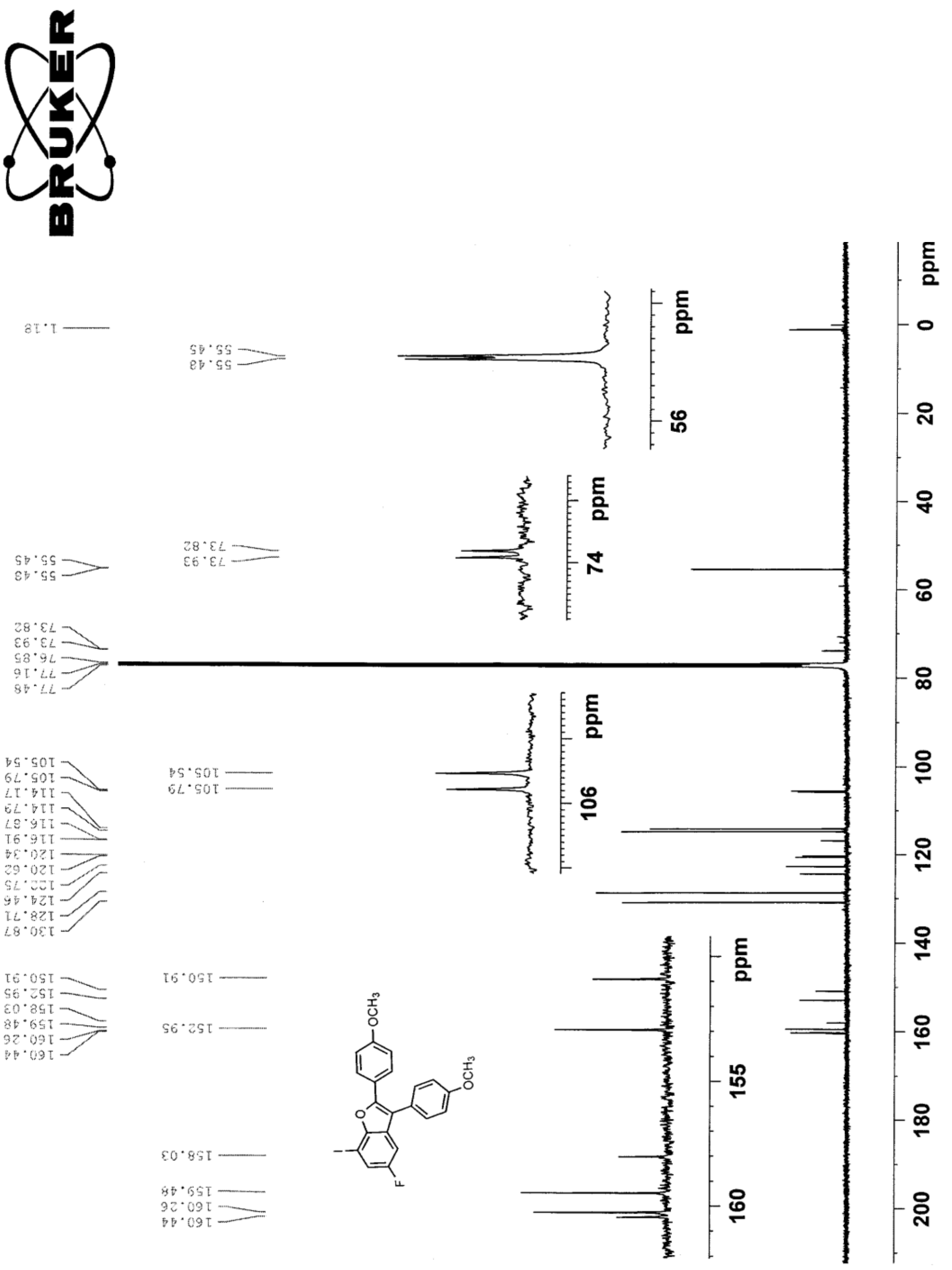

R. M. Al-Zoubi, W. K. Al-Jammal, M.J. Ferguson and G. K. Murphy

1.3.23 ^{1}H-NMR of 5-chloro-7-iodo-2,3-bis(4-methoxyphenyl)benzofuran (7l) in d-$CDCl_3$ at 25 °C

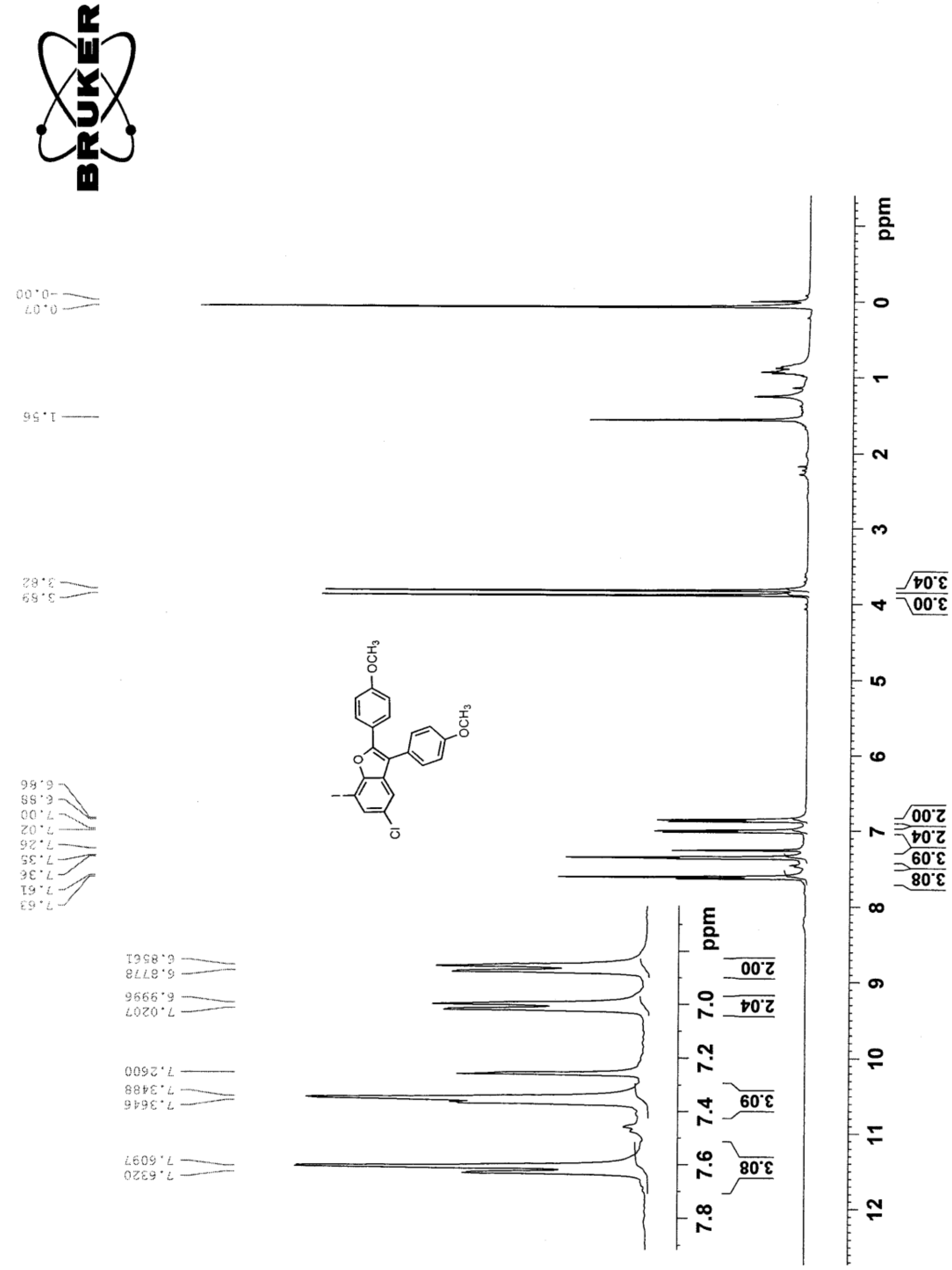

1.3.24 ^{13}C-NMR of 5-chloro-7-iodo-2,3-bis(4-methoxyphenyl)benzofuran (7l) in d-$CDCl_3$ at 25 ^oC.

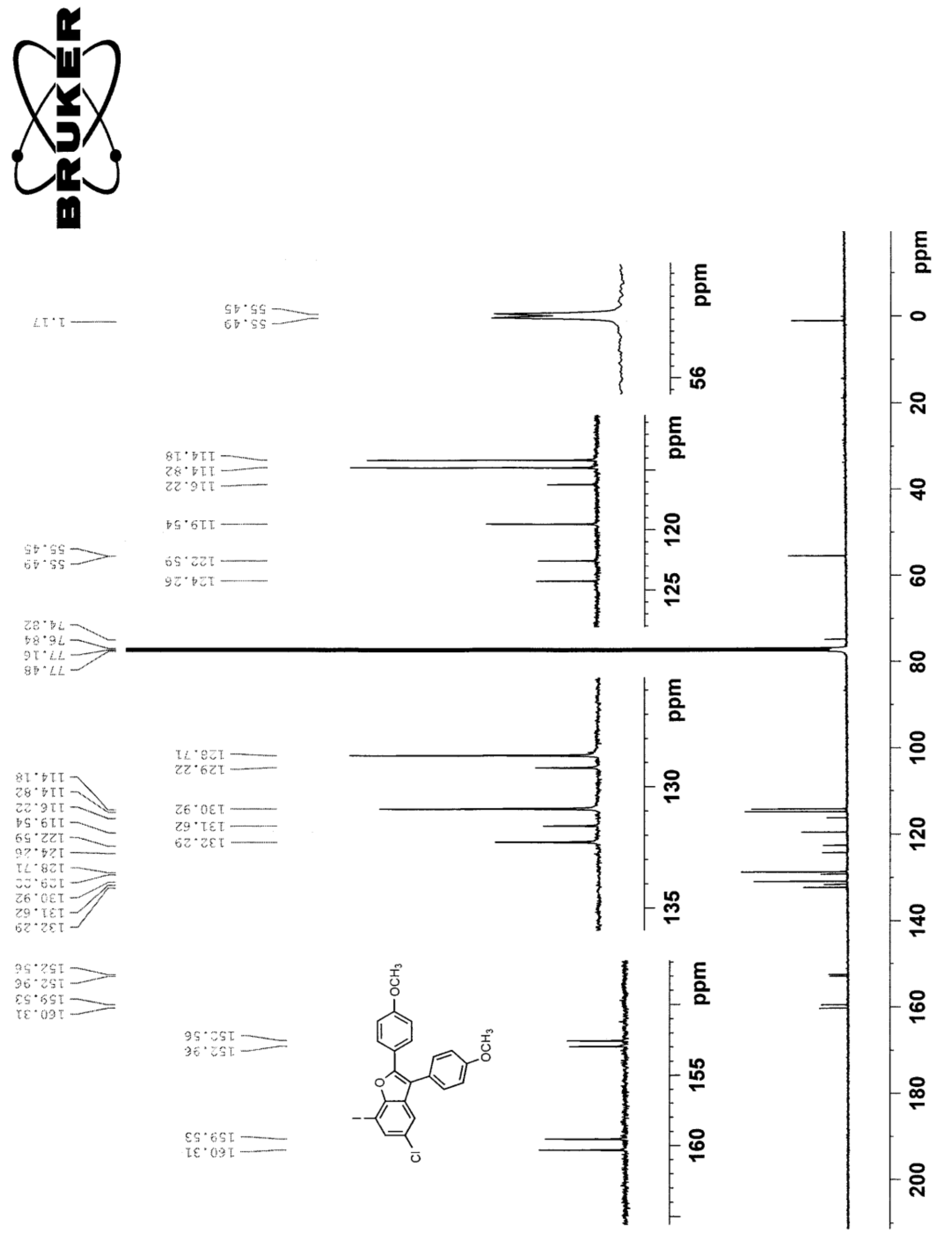

1.3.25 ^{1}H-NMR of 5-chloro-7-iodo-2,3-diphenylbenzofuran (7m) in d-$CDCl_3$ at 25 ^{o}C

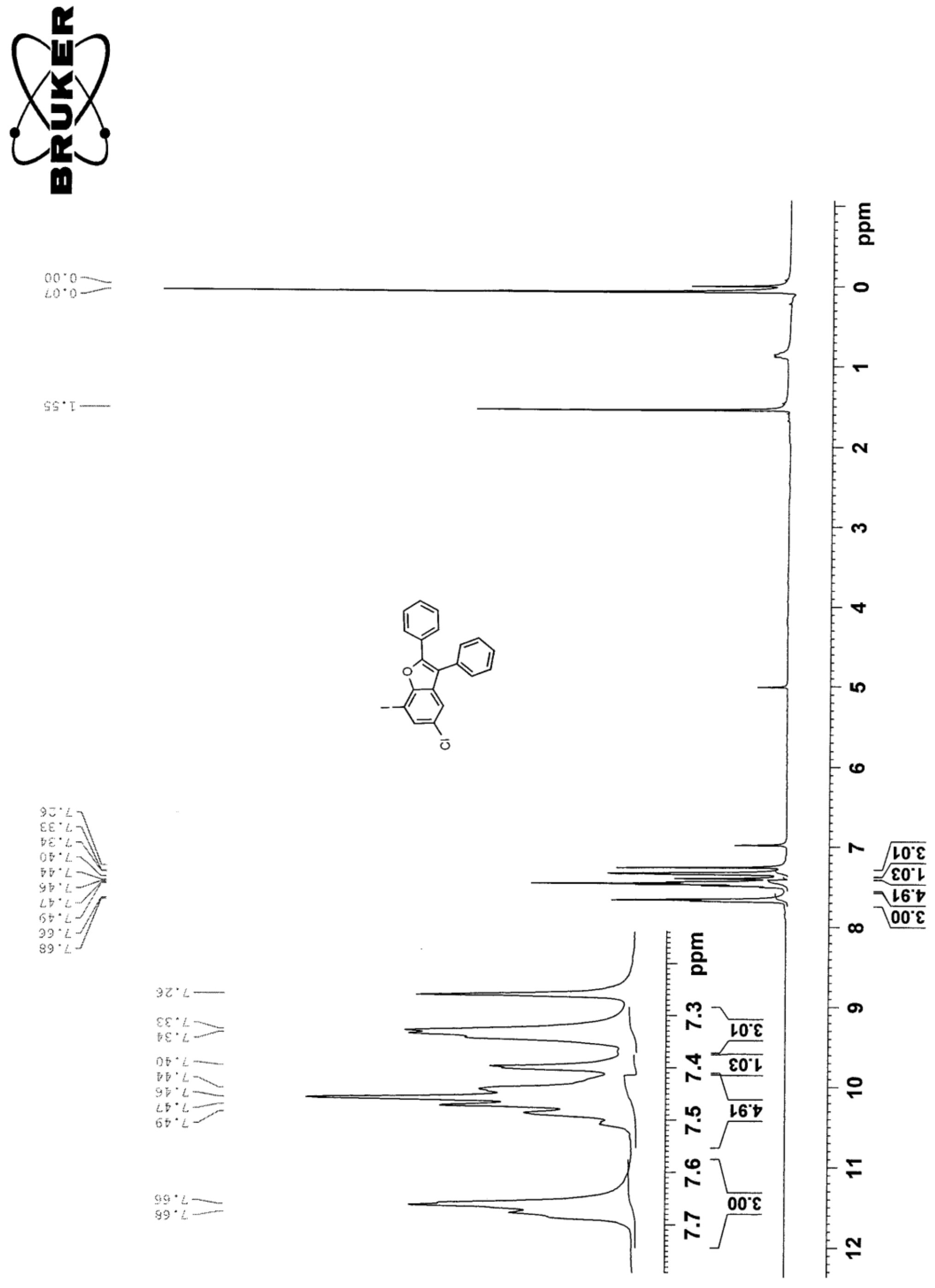

1.3.26 ^{13}C-NMR of 5-chloro-7-iodo-2,3-diphenylbenzofuran (7m) in d-$CDCl_3$ at 25 ^{o}C.

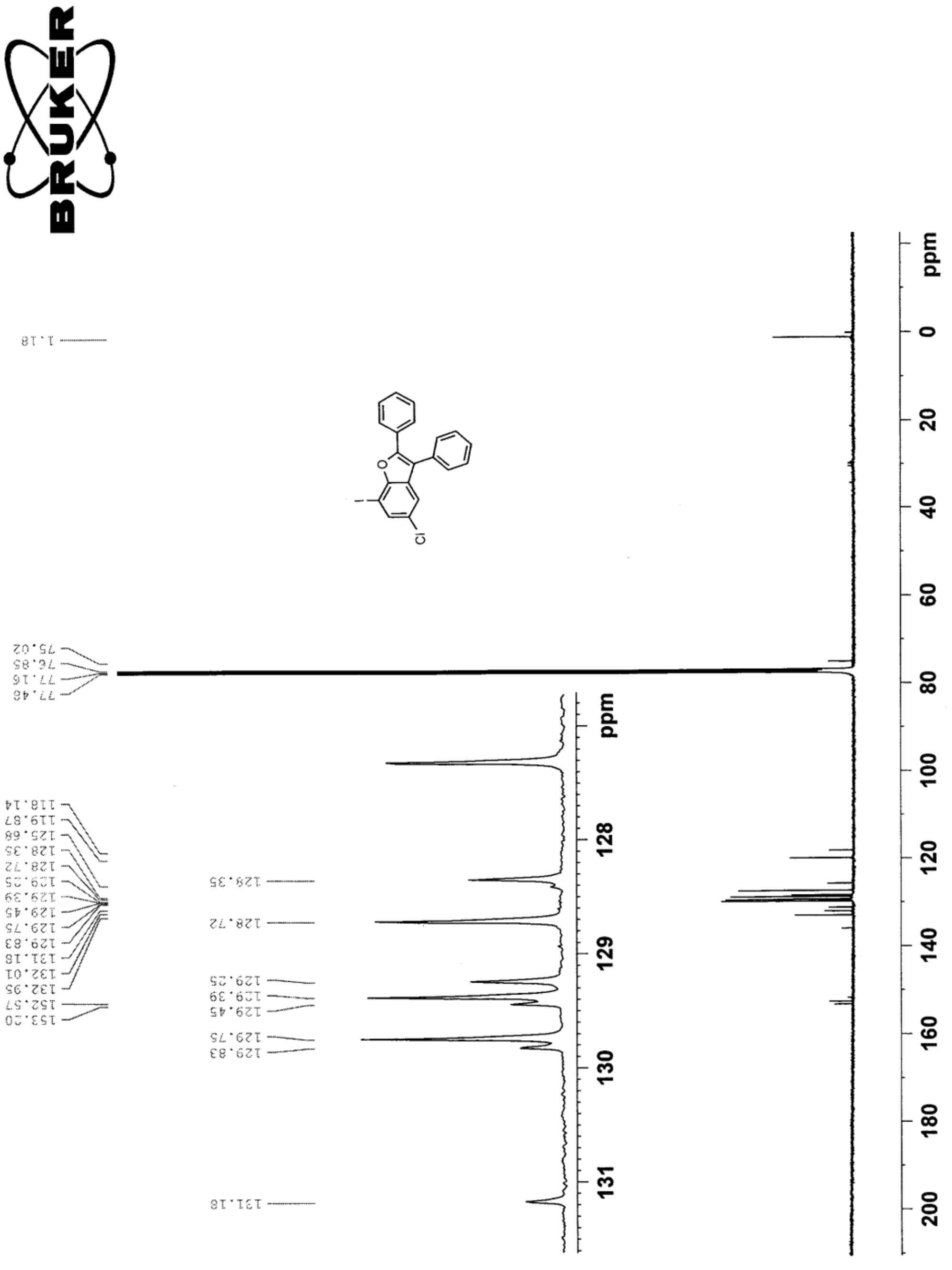

R. M. Al-Zoubi, W. K. Al-Jammal, M.J. Ferguson and G. K. Murphy

1.3.27 ^{1}H-NMR of 2-benzyl-5-chloro-7-iodo-3-phenylbenzofuran (7n) in d-$CDCl_3$ at 25 °C

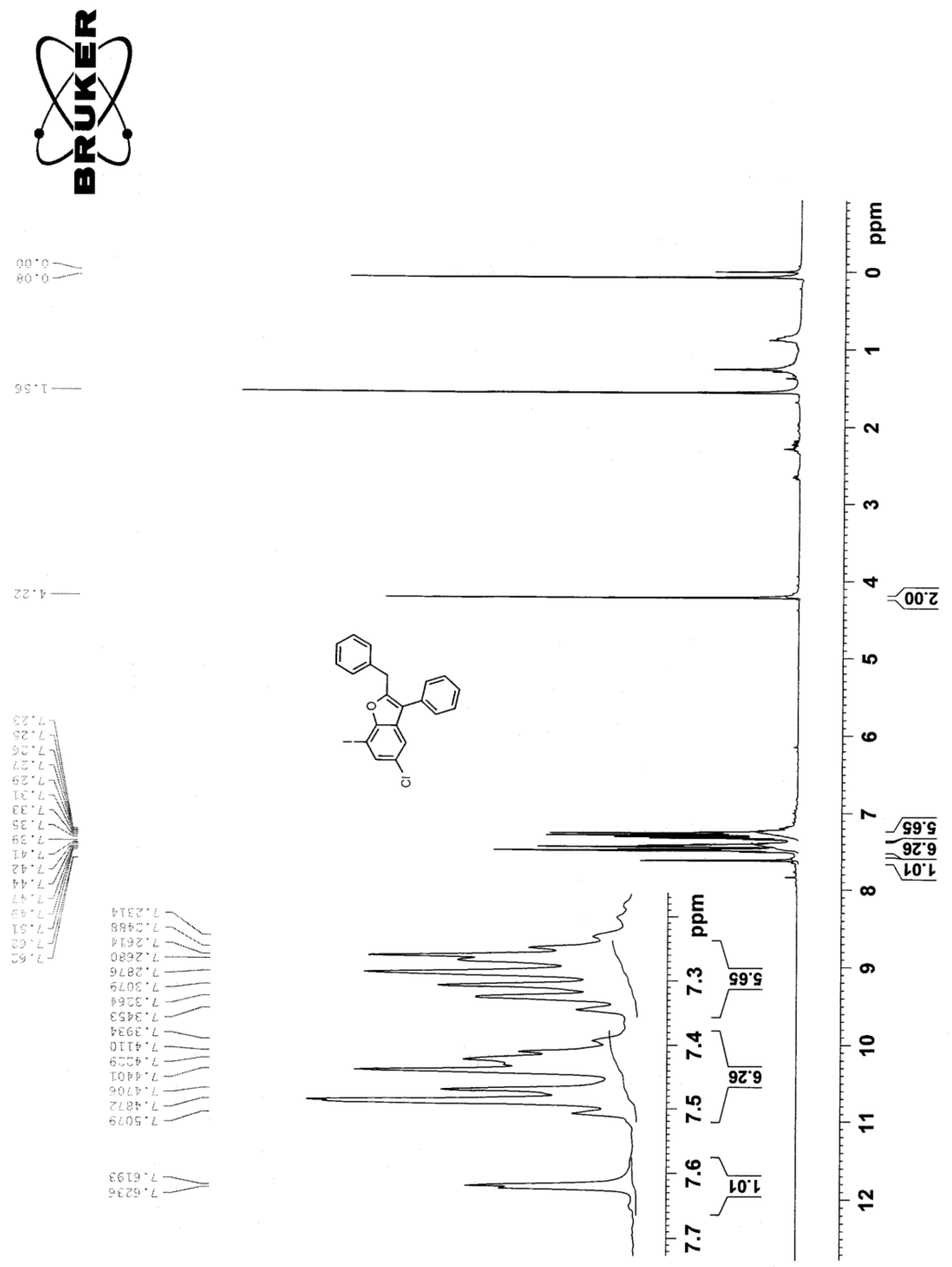

1.3.28 ^{13}C-NMR of 2-benzyl-5-chloro-7-iodo-3-phenylbenzofuran (7n) in d-$CDCl_3$ at 25 °C.

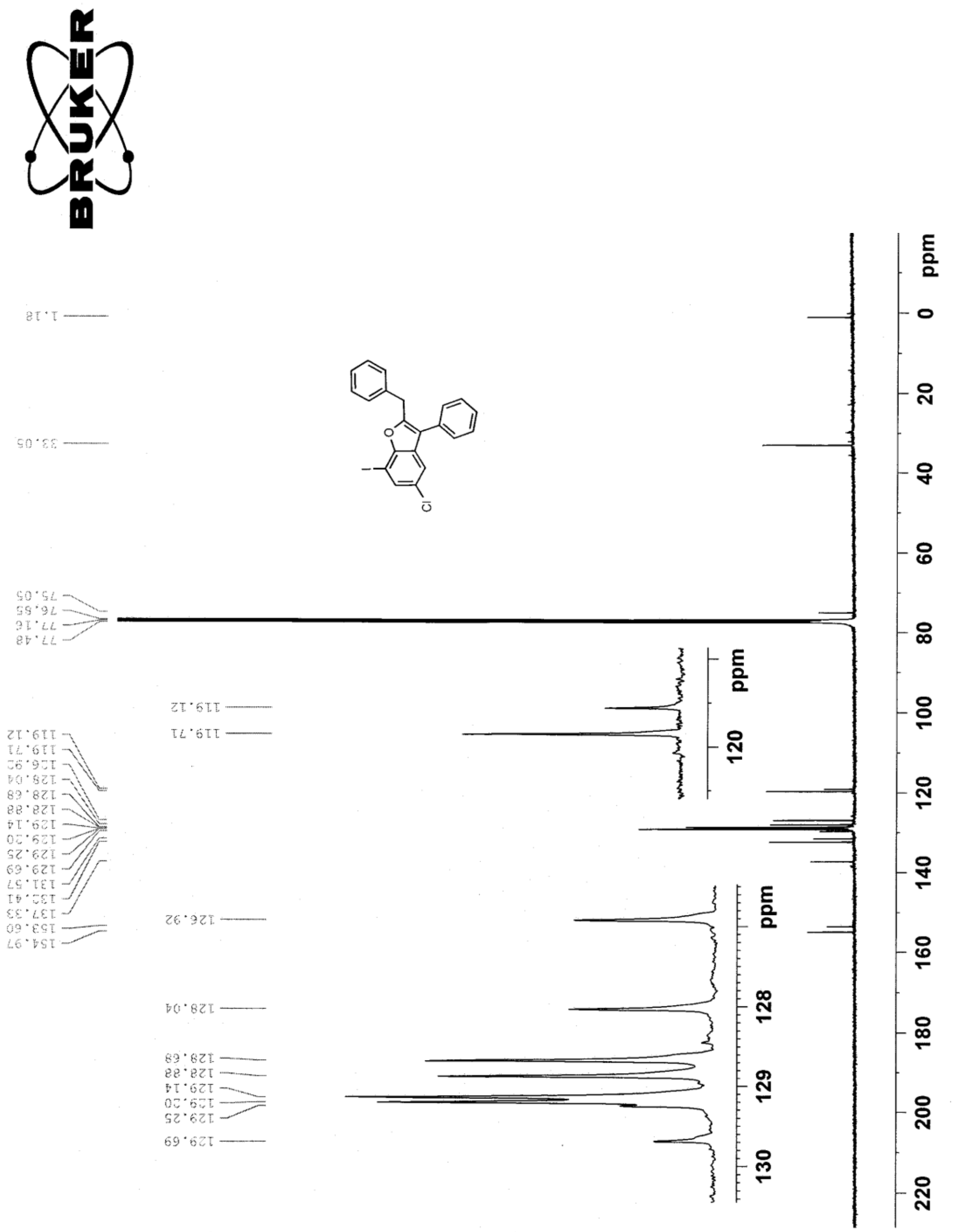

1.3.29 ^{1}H-NMR of 5-bromo-7-iodo-2,3-bis(4-methoxyphenyl)benzofuran (7p) in d-$CDCl_3$ at 25 ^oC

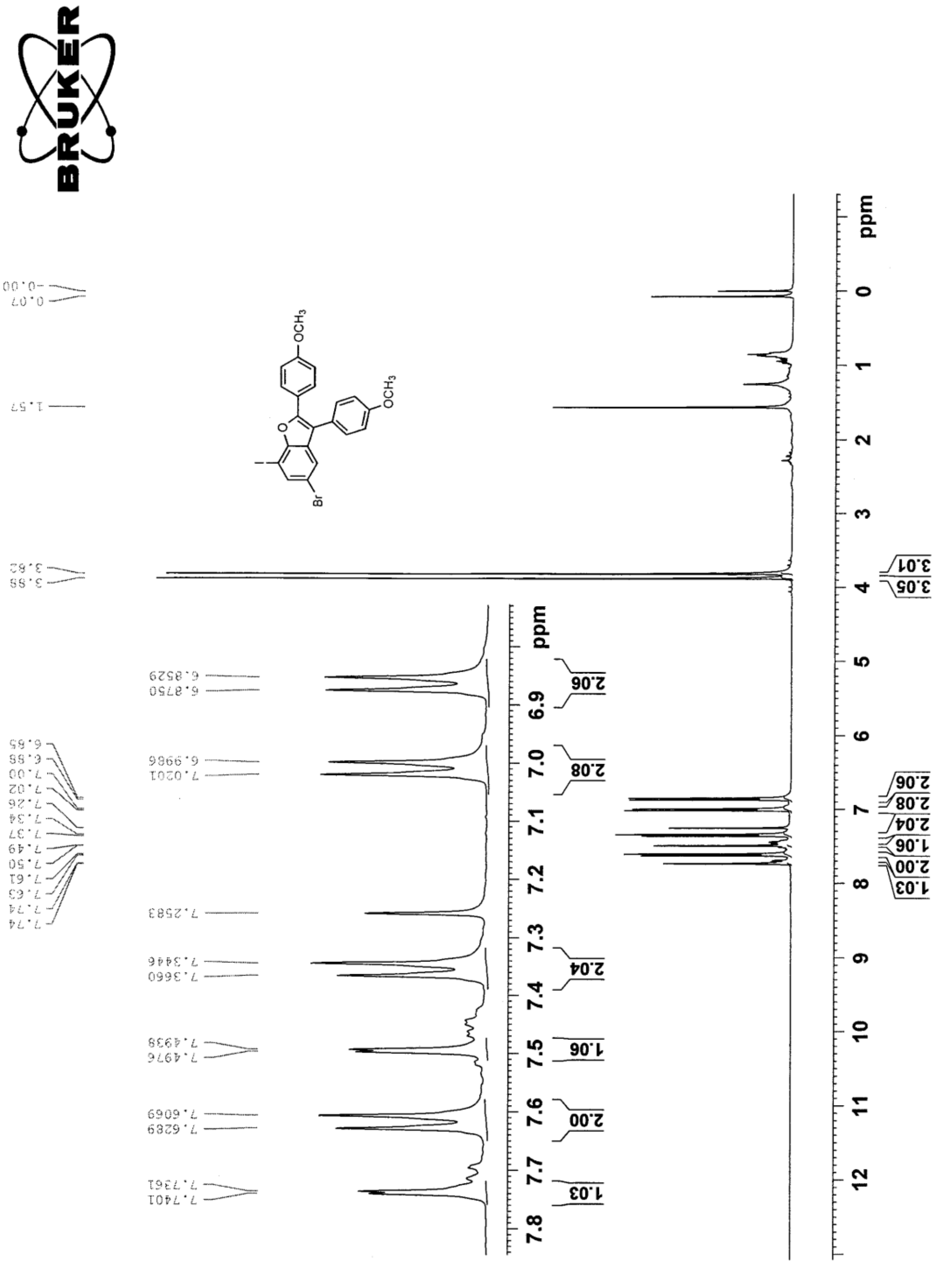

1.3.30 ^{13}C-NMR of 5-bromo-7-iodo-2,3-bis(4-methoxyphenyl)benzofuran (7p) in d-$CDCl_3$ at 25 °C.

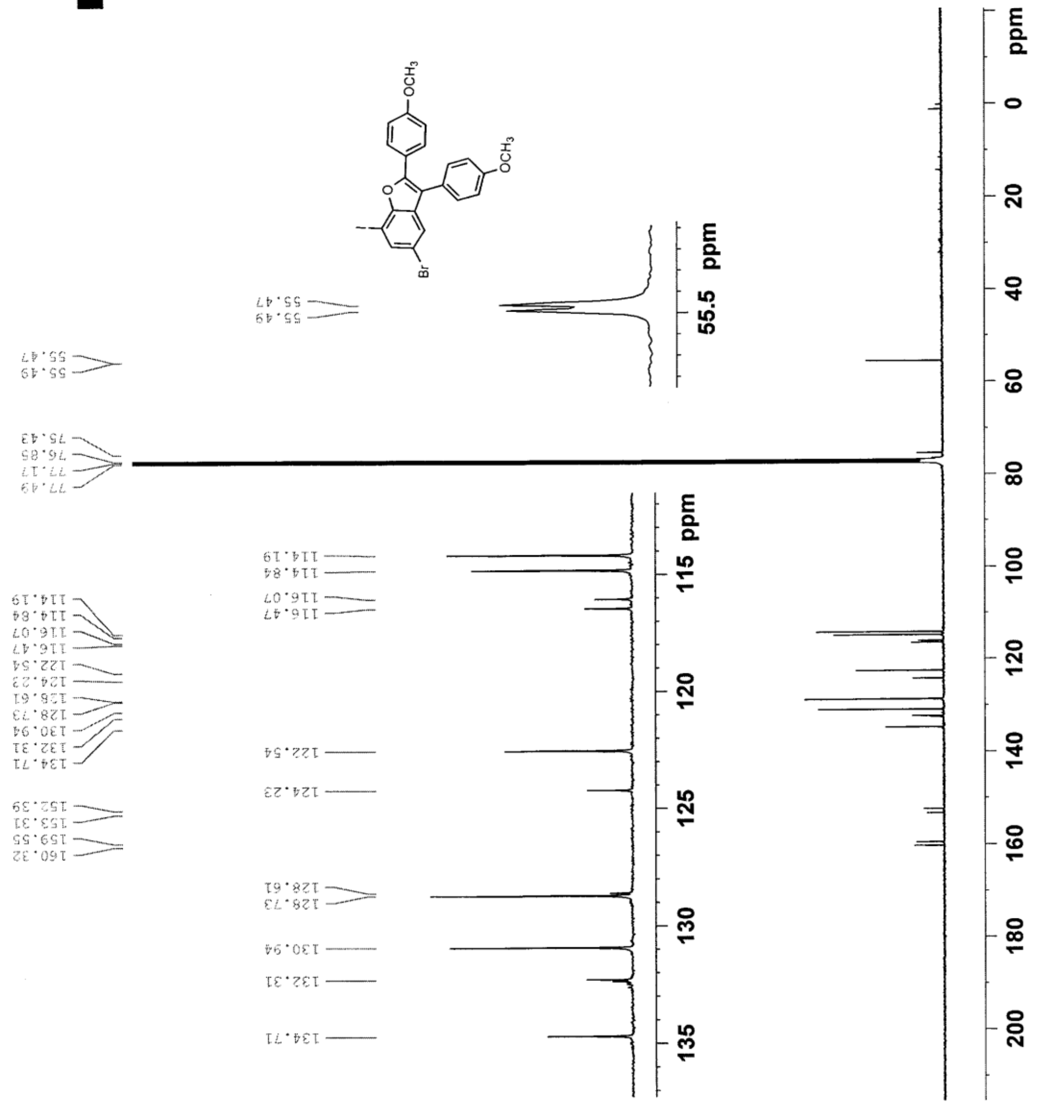

1.3.31 ¹H-NMR of methyl 7-iodo-2,3-bis(4-methoxyphenyl)benzofuran-5-carboxylate (7q) in d-$CDCl_3$ at 25 °C

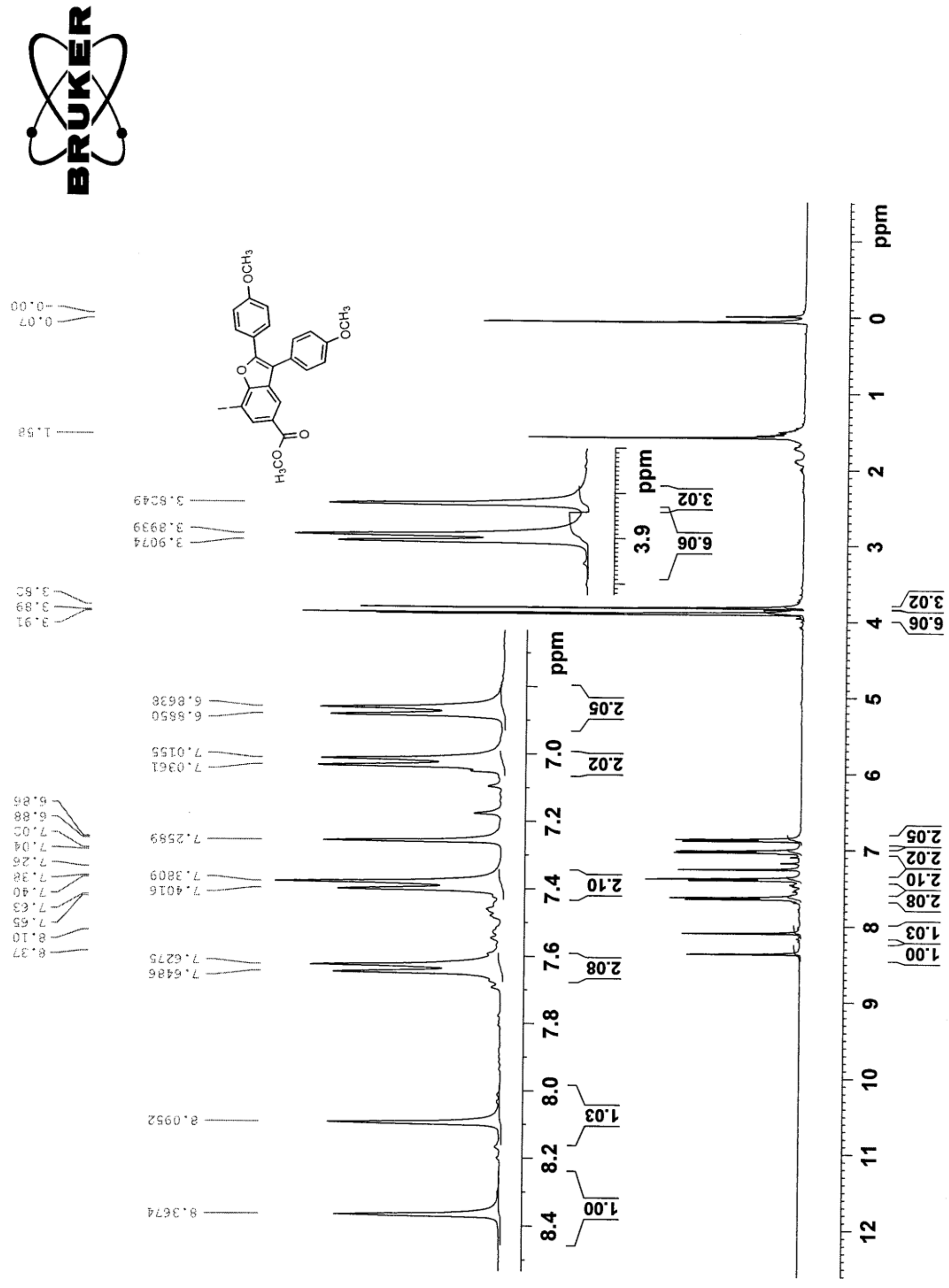

1.3.32 ^{13}C-NMR of methyl 7-iodo-2,3-bis(4-methoxyphenyl)benzofuran-5-carboxylate (7q) in d-$CDCl_3$ at 25 °C.

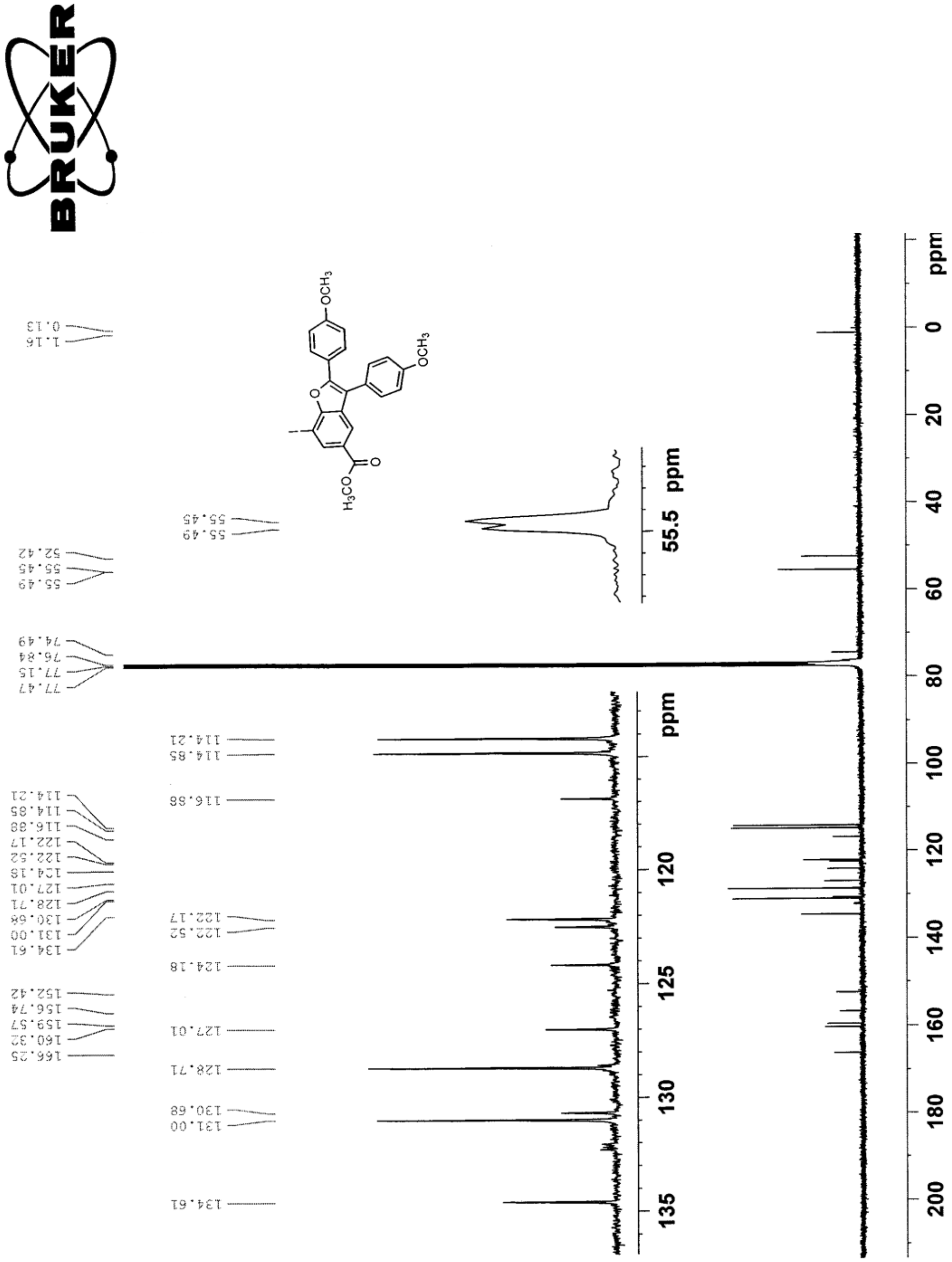

1.3.33 ^{1}H-NMR of 7-chloro-2,3-bis(4-methoxyphenyl)benzofuran (7r) in d-$CDCl_3$ at 25 ^oC

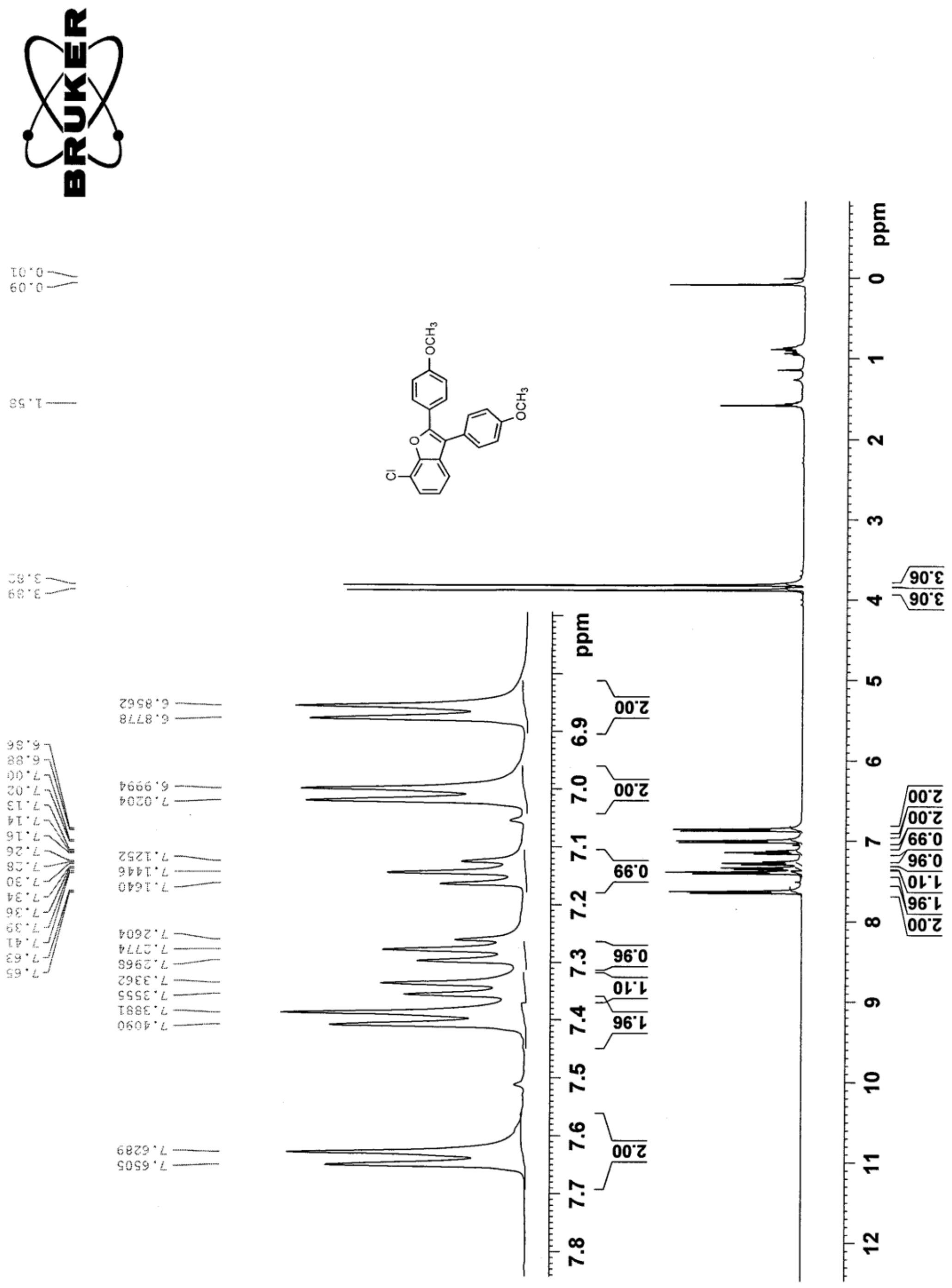

1.3.34 ^{13}C-NMR of 7-chloro-2,3-bis(4-methoxyphenyl)benzofuran (7r) in d-$CDCl_3$ at 25 °C.

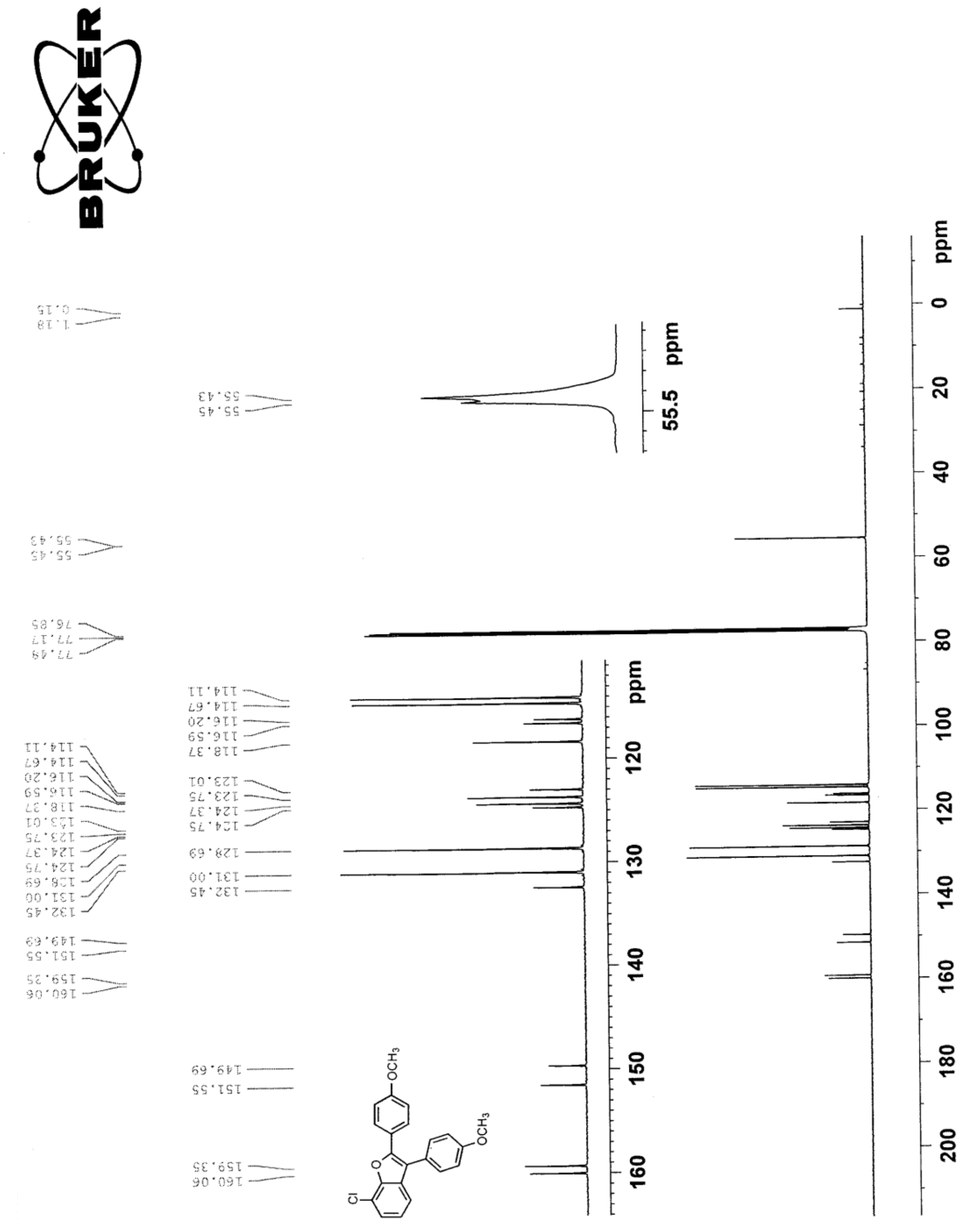

1.3.35 ^{1}H-NMR of 7-bromo-2,3-bis(4-methoxyphenyl)benzofuran (7t) in d-CDCl$_3$ at 25 ^{o}C

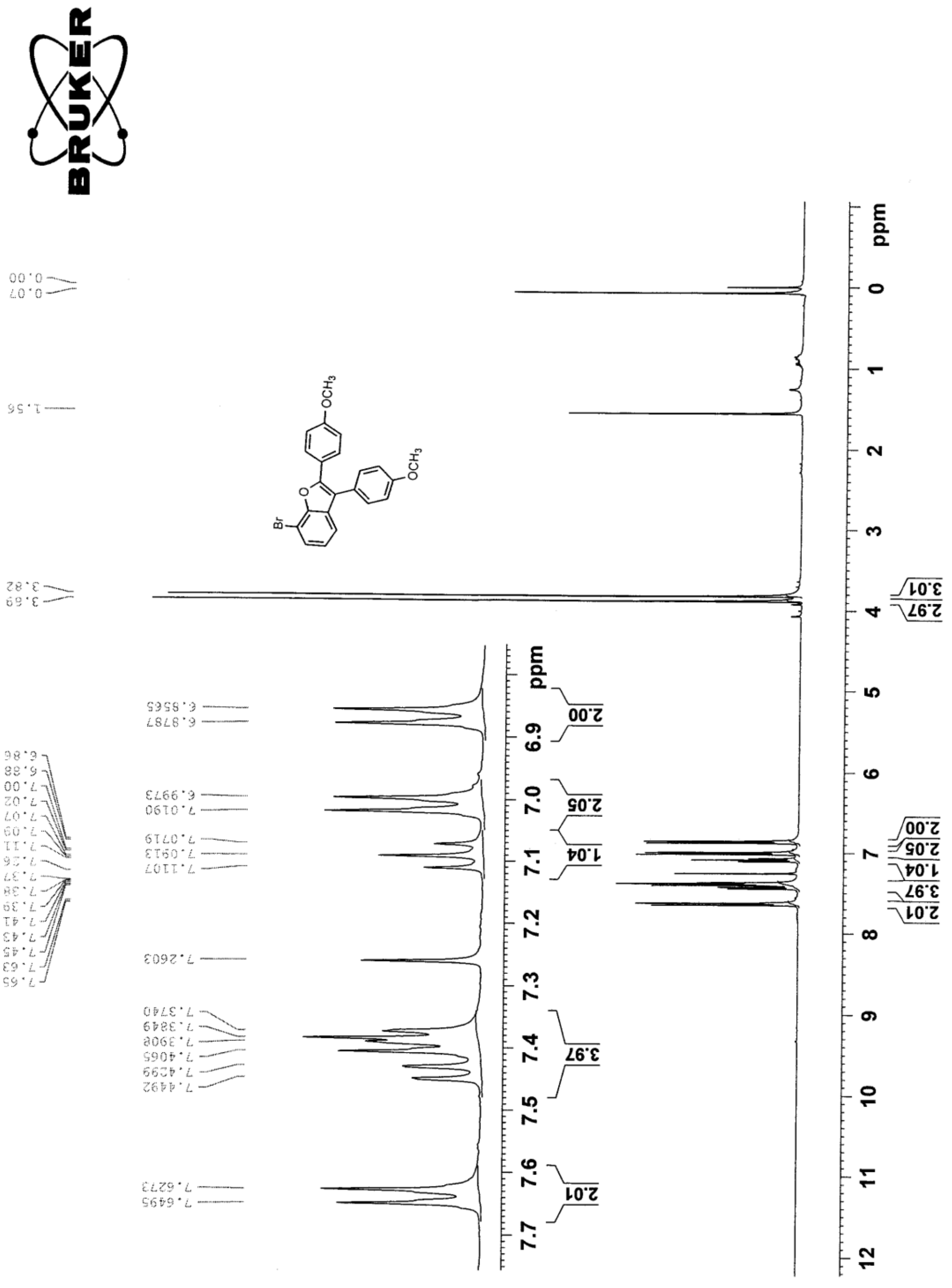

1.3.36 ¹³C-NMR of 7-bromo-2,3-bis(4-methoxyphenyl)benzofuran (7t) in d-CDCl₃ at 25 °C.

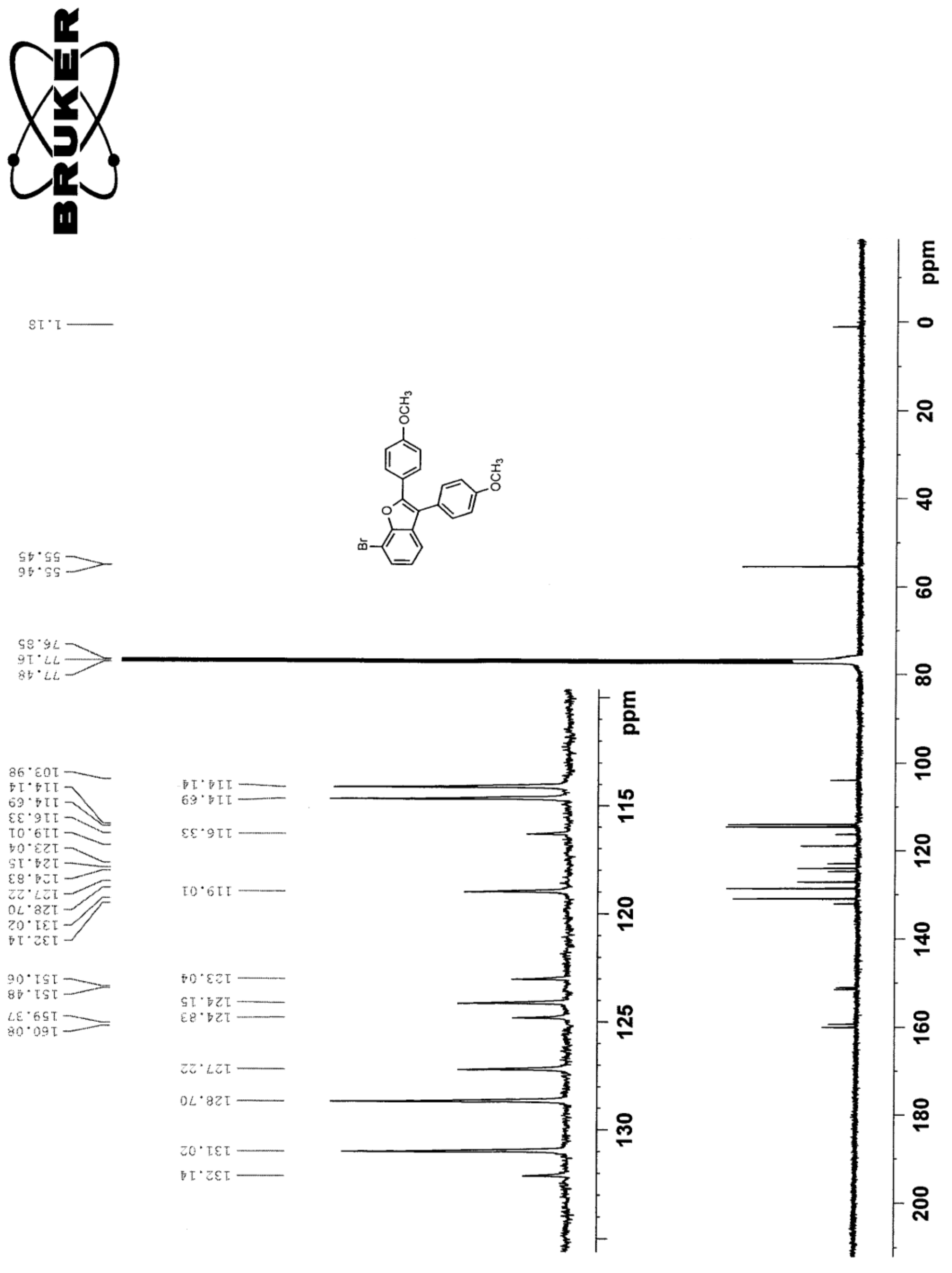

1.3.37 ^{1}H-NMR of 1-(5-chloro-2,3-diiodophenyl)-1-(4-methoxyphenyl)propan-2-one in d-$CDCl_3$ at 25 ^{o}C

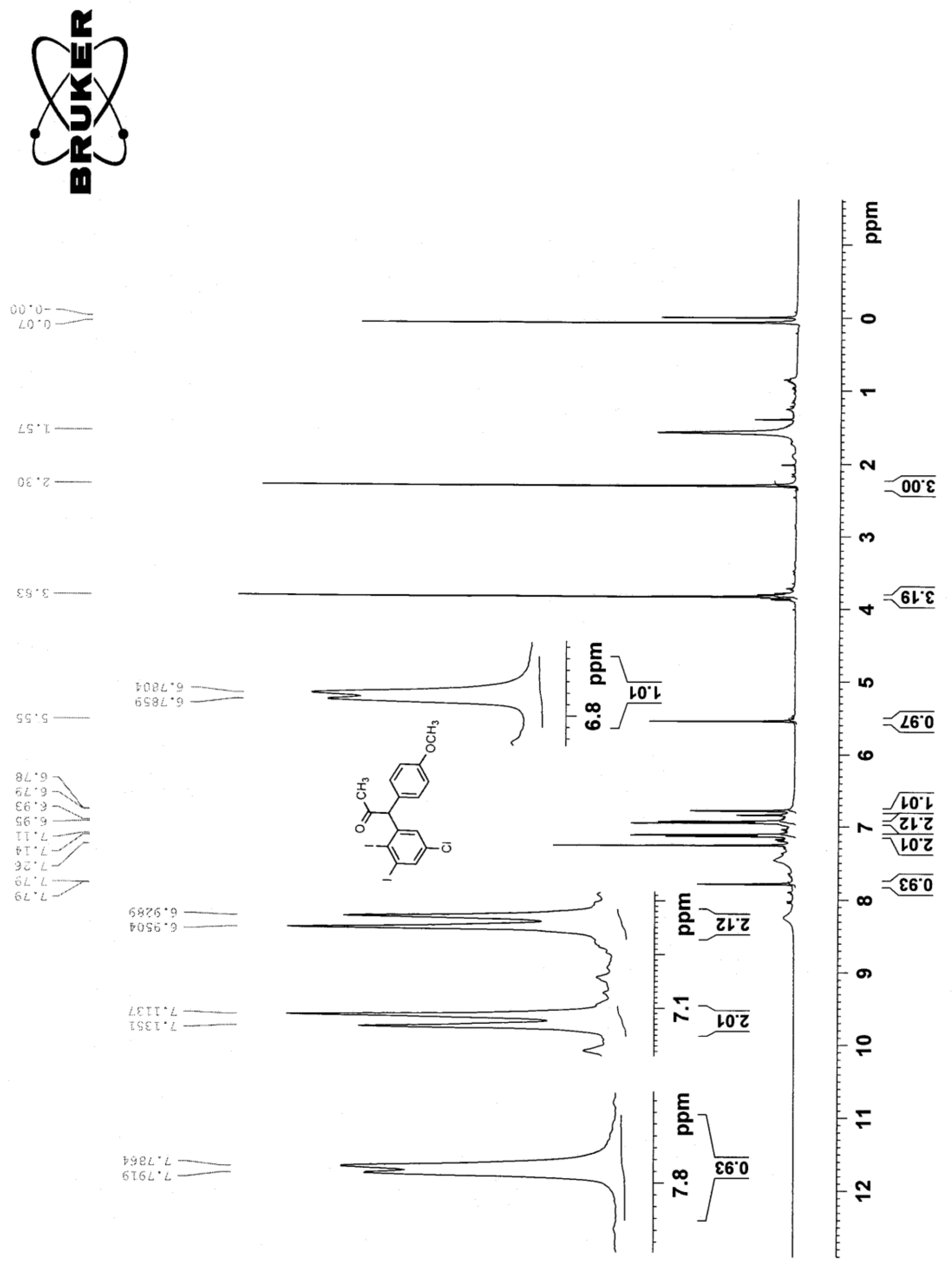

1.3.38 ^{13}C-NMR of 1-(5-chloro-2,3-diiodophenyl)-1-(4-methoxyphenyl)propan-2-one in d-$CDCl_3$ at 25 °C.

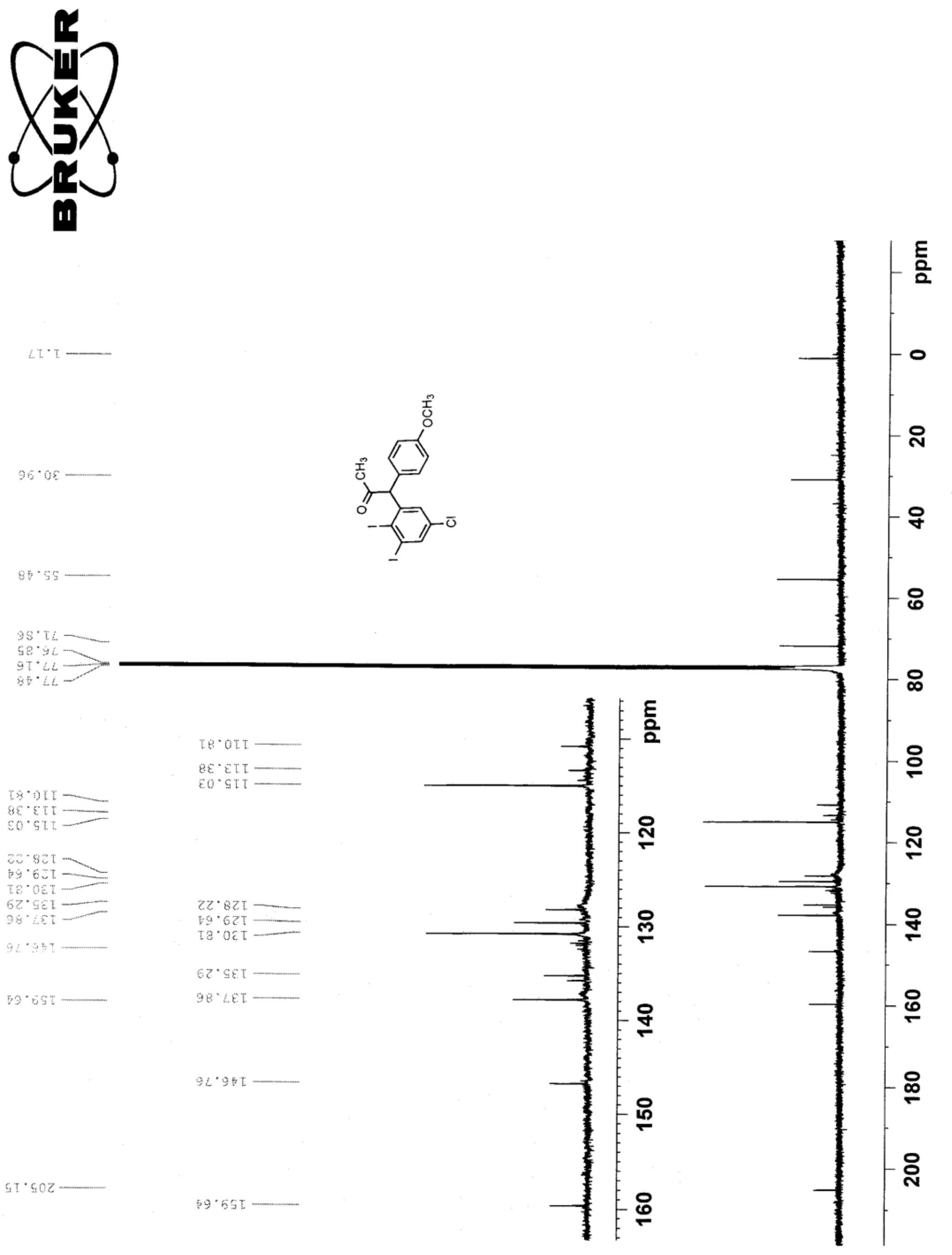

1.4.1 X-ray data of 2-(4-chlorophenyl)-7-iodo-3-phenyl-1-benzofuran (**7c**)

STRUCTURE REPORT

XCL Code: JUS1802 **Date:** 19 September 2018

Compound: 2-(4-chlorophenyl)-7-iodo-3-phenyl-1-benzofuran
Formula: $C_{20}H_{12}ClIO$

Supervisor: R. Al-Zoubi, Jordan University of Science and Technology

Crystallographer: M. J. Ferguson

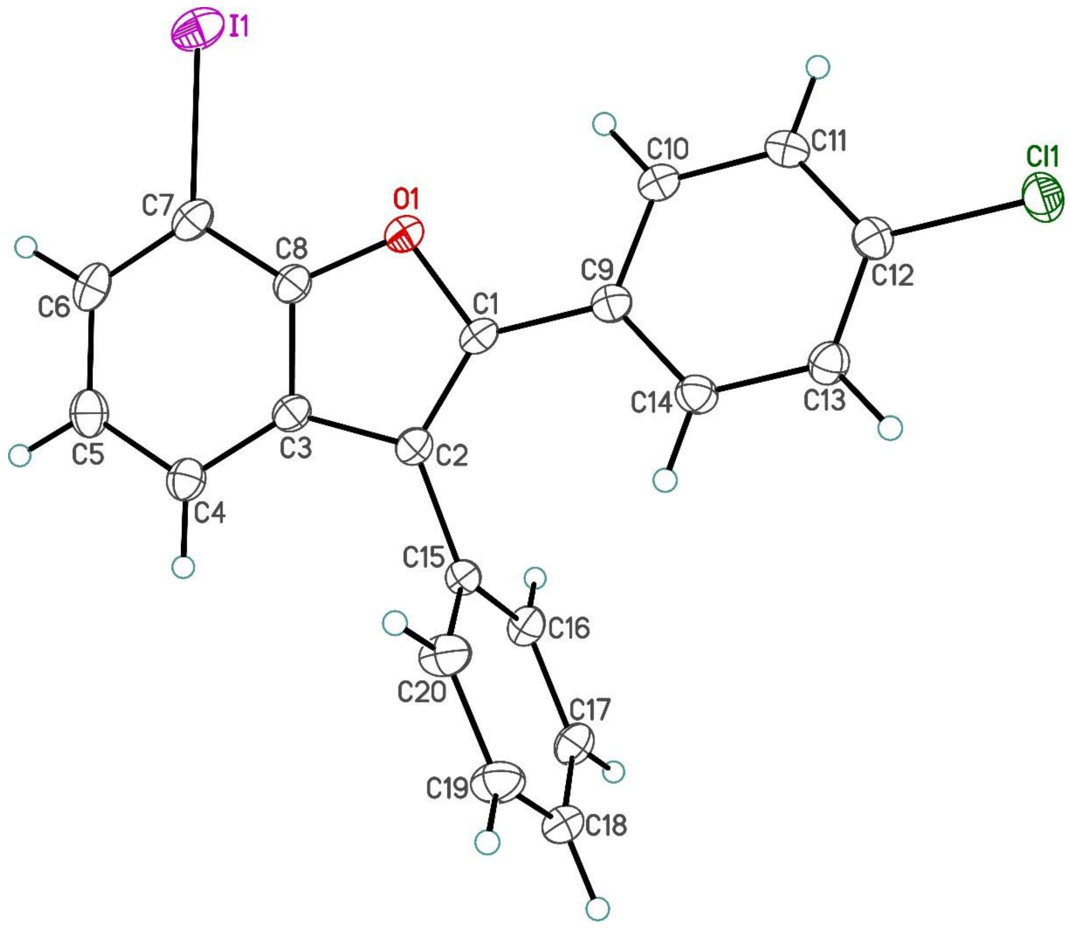

Figure Legends

Figure 1. Perspective view of the 2-(4-chlorophenyl)-7-iodo-3-phenyl-1-benzofuran molecule showing the atom labelling scheme. Non-hydrogen atoms are represented by Gaussian ellipsoids at the 30% probability level. Hydrogen atoms are shown with arbitrarily small thermal parameters.

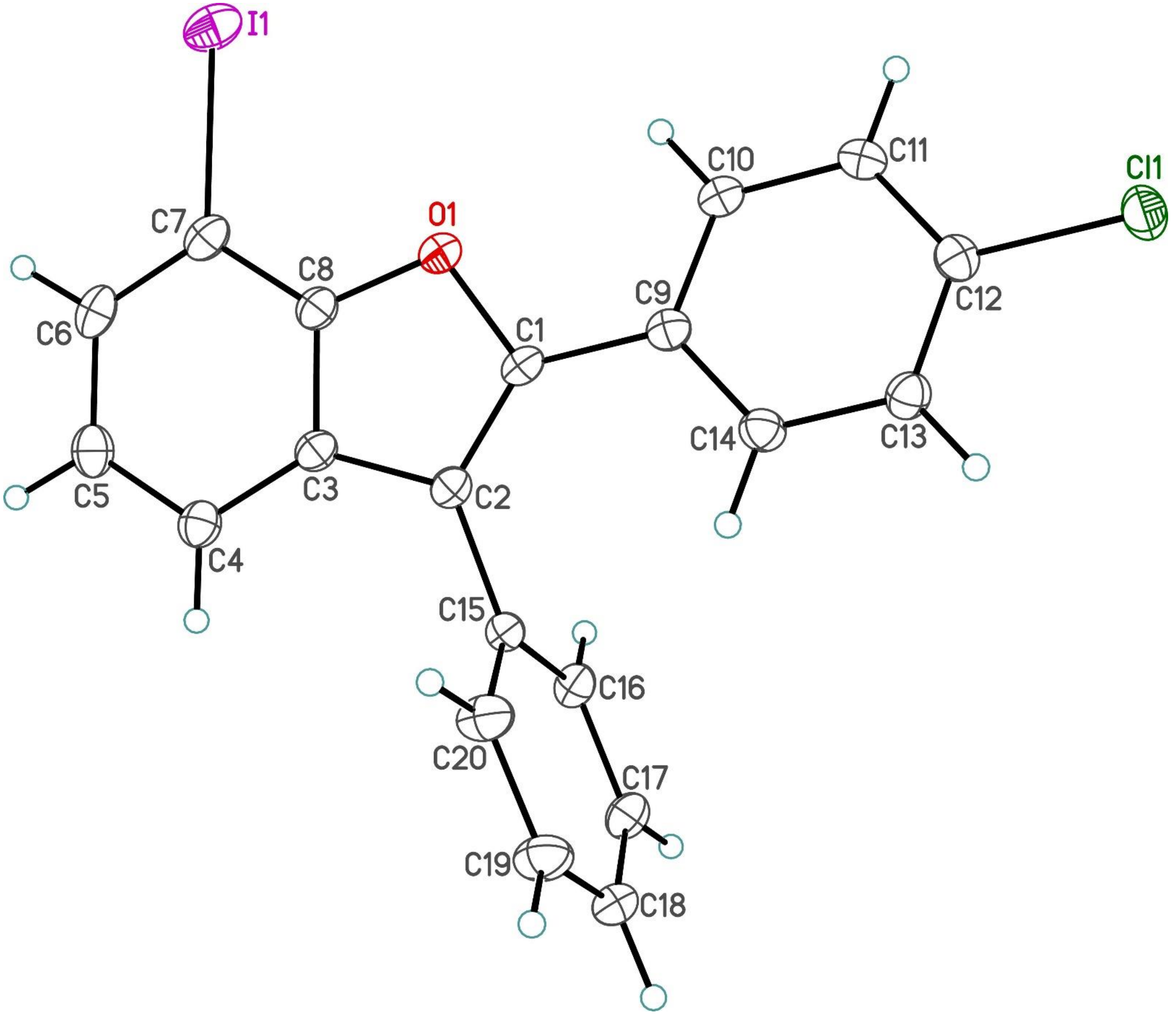
I1
O1
Cl1
C1
C2
C3
C4
C5
C6
C7
C8
C9
C10
C11
C12
C13
C14
C15
C16
C17
C18
C19
C20

List of Tables

Table 1. Crystallographic Experimental Details

Table 2. Atomic Coordinates and Equivalent Isotropic Displacement Parameters

Table 3. Selected Interatomic Distances

Table 4. Selected Interatomic Angles

Table 5. Torsional Angles

Table 6. Anisotropic Displacement Parameters

Table 7. Derived Atomic Coordinates and Displacement Parameters for Hydrogen Atoms

Table 1. Crystallographic Experimental Details

A. Crystal Data	
formula	$C_{20}H_{12}ClIO$
formula weight	430.65
crystal dimensions (mm)	0.23 × 0.19 × 0.04
crystal system	triclinic
space group	$P\bar{1}$ (No. 2)
unit cell parameters[a]	
a (Å)	9.244(2)
b (Å)	9.988(3)
c (Å)	10.515(3)
α (deg)	86.607(3)
β (deg)	65.448(3)
γ (deg)	70.670(3)
V (Å^3)	829.8(4)
Z	2
ρ_{calcd} (g cm^{-3})	1.724
μ (mm^{-1})	2.092
B. Data Collection and Refinement Conditions	
diffractometer	Bruker PLATFORM/APEX II CCD[b]
radiation (λ [Å])	graphite-monochromated Mo Kα (0.71073)
temperature (°C)	–80
scan type	ω scans (0.3°) (20 s exposures)
data collection 2θ limit (deg)	55.27
total data collected	7499 ($-12 \le h \le 12$, $-13 \le k \le 13$, $-13 \le l \le 13$)
independent reflections	3843 (R_{int} = 0.0149)
number of observed reflections (*NO*)	3365 [$F_o^2 \ge 2\sigma(F_o^2)$]
structure solution method	intrinsic phasing (*SHELXT-2014*[c])
refinement method	full-matrix least-squares on F^2 (*SHELXL–2016*[d])
absorption correction method	Gaussian integration (face-indexed)
range of transmission factors	0.9393–0.7307
data/restraints/parameters	3843 / 0 / 208
goodness-of-fit (S)[e] [all data]	1.051
final R indices[f]	
R_1 [$F_o^2 \ge 2\sigma(F_o^2)$]	0.0265
wR_2 [all data]	0.0676
largest difference peak and hole	0.636 and –0.727 e Å^{-3}

[a]Obtained from least-squares refinement of 6231 reflections with 4.34° < 2θ < 55.24°.

(continued)

Table 1. Crystallographic Experimental Details (continued)

[b]Programs for diffractometer operation, data collection, data reduction and absorption correction were those supplied by Bruker

[c]Sheldrick, G. M. *Acta Crystallogr.* **2015**, *A71*, 3–8. (*SHELXT-2014*)

[d]Sheldrick, G. M. *Acta Crystallogr.* **2015**, *C71*, 3–8. (*SHELXL-2016*)

[e]$S = [\Sigma w(F_o^2 - F_c^2)^2/(n - p)]^{1/2}$ (n = number of data; p = number of parameters varied; $w = [\sigma^2(F_o^2) + (0.0329P)^2 + 0.3747P]^{-1}$ where $P = [\mathrm{Max}(F_o^2, 0) + 2F_c^2]/3$).

[f]$R_1 = \Sigma||F_o| - |F_c||/\Sigma|F_o|$; $wR_2 = [\Sigma w(F_o^2 - F_c^2)^2/\Sigma w(F_o^4)]^{1/2}$.

Table 2. Atomic Coordinates and Equivalent Isotropic Displacement Parameters

Atom	*x*	*y*	*z*	U_{eq}, Å^2
I1	0.79344(2)	-0.01350(2)	0.34954(2)	0.04862(8)*
Cl1	-0.27026(7)	0.67509(6)	0.82007(6)	0.03918(14)*
O1	0.52940(19)	0.31735(15)	0.37816(16)	0.0291(3)*
C1	0.4391(3)	0.4592(2)	0.3730(2)	0.0267(4)*
C2	0.5323(3)	0.5162(2)	0.2616(2)	0.0274(4)*
C3	0.6931(3)	0.4057(2)	0.1897(2)	0.0275(4)*
C4	0.8424(3)	0.3965(3)	0.0697(2)	0.0330(5)*
C5	0.9737(3)	0.2671(3)	0.0317(3)	0.0368(5)*
C6	0.9607(3)	0.1487(3)	0.1081(3)	0.0360(5)*
C7	0.8149(3)	0.1571(2)	0.2265(2)	0.0326(5)*
C8	0.6826(3)	0.2866(2)	0.2648(2)	0.0283(4)*
C9	0.2664(3)	0.5130(2)	0.4836(2)	0.0271(4)*
C10	0.1913(3)	0.4185(2)	0.5657(2)	0.0300(4)*
C11	0.0271(3)	0.4674(2)	0.6693(2)	0.0324(5)*
C12	-0.0633(3)	0.6122(2)	0.6910(2)	0.0301(4)*
C13	0.0079(3)	0.7085(2)	0.6113(2)	0.0311(5)*
C14	0.1720(3)	0.6590(2)	0.5086(2)	0.0304(5)*
C15	0.4773(3)	0.6594(2)	0.2136(2)	0.0267(4)*
C16	0.3862(3)	0.6816(2)	0.1326(2)	0.0311(5)*
C17	0.3306(3)	0.8156(2)	0.0891(3)	0.0350(5)*
C18	0.3652(3)	0.9283(2)	0.1257(3)	0.0372(5)*
C19	0.4568(4)	0.9065(3)	0.2050(3)	0.0427(6)*
C20	0.5144(3)	0.7729(3)	0.2481(3)	0.0386(5)*

Anisotropically-refined atoms are marked with an asterisk (*). The form of the anisotropic displacement parameter is: $\exp[-2\pi^2(h^2a^{*2}U_{11} + k^2b^{*2}U_{22} + l^2c^{*2}U_{33} + 2klb^*c^*U_{23} + 2hla^*c^*U_{13} + 2hka^*b^*U_{12})]$.

Table 3. Selected Interatomic Distances (Å)

Atom1	Atom2	Distance	Atom1	Atom2	Distance
I1	C7	2.090(2)	C7	C8	1.391(3)
Cl1	C12	1.744(2)	C9	C10	1.399(3)
O1	C1	1.397(2)	C9	C14	1.403(3)
O1	C8	1.369(3)	C10	C11	1.389(3)
C1	C2	1.359(3)	C11	C12	1.387(3)
C1	C9	1.467(3)	C12	C13	1.388(3)
C2	C3	1.442(3)	C13	C14	1.384(3)
C2	C15	1.485(3)	C15	C16	1.390(3)
C3	C4	1.409(3)	C15	C20	1.394(3)
C3	C8	1.397(3)	C16	C17	1.390(3)
C4	C5	1.387(3)	C17	C18	1.382(3)
C5	C6	1.400(4)	C18	C19	1.379(4)
C6	C7	1.384(3)	C19	C20	1.389(4)

Table 4. Selected Interatomic Angles (deg)

Atom1	Atom2	Atom3	Angle	Atom1	Atom2	Atom3	Angle
C1	O1	C8	105.83(16)	C1	C9	C10	120.22(19)
O1	C1	C2	111.13(18)	C1	C9	C14	121.30(19)
O1	C1	C9	114.90(18)	C10	C9	C14	118.5(2)
C2	C1	C9	133.95(19)	C9	C10	C11	121.0(2)
C1	C2	C3	106.62(18)	C10	C11	C12	119.2(2)
C1	C2	C15	127.57(19)	Cl1	C12	C11	119.73(17)
C3	C2	C15	125.60(19)	Cl1	C12	C13	119.20(17)
C2	C3	C4	134.8(2)	C11	C12	C13	121.1(2)
C2	C3	C8	105.60(19)	C12	C13	C14	119.4(2)
C4	C3	C8	119.6(2)	C9	C14	C13	120.9(2)
C3	C4	C5	117.6(2)	C2	C15	C16	119.56(19)
C4	C5	C6	122.0(2)	C2	C15	C20	121.5(2)
C5	C6	C7	120.8(2)	C16	C15	C20	118.9(2)
I1	C7	C6	122.23(17)	C15	C16	C17	120.4(2)
I1	C7	C8	120.32(17)	C16	C17	C18	120.5(2)
C6	C7	C8	117.4(2)	C17	C18	C19	119.4(2)
O1	C8	C3	110.80(18)	C18	C19	C20	120.7(2)
O1	C8	C7	126.6(2)	C15	C20	C19	120.1(2)
C3	C8	C7	122.6(2)				

Table 5. Torsional Angles (deg)

Atom1	Atom2	Atom3	Atom4	Angle	Atom1	Atom2	Atom3	Atom4	Angle
C8	O1	C1	C2	-0.6(2)	C3	C4	C5	C6	0.2(3)
C8	O1	C1	C9	177.81(17)	C4	C5	C6	C7	-0.6(3)
C1	O1	C8	C3	1.2(2)	C5	C6	C7	I1	-177.03(17)
C1	O1	C8	C7	-179.9(2)	C5	C6	C7	C8	0.7(3)
O1	C1	C2	C3	-0.1(2)	I1	C7	C8	O1	-1.6(3)
O1	C1	C2	C15	174.78(19)	I1	C7	C8	C3	177.24(15)
C9	C1	C2	C3	-178.2(2)	C6	C7	C8	O1	-179.41(19)
C9	C1	C2	C15	-3.3(4)	C6	C7	C8	C3	-0.6(3)
O1	C1	C9	C10	-13.2(3)	C1	C9	C10	C11	-178.9(2)
O1	C1	C9	C14	167.77(18)	C14	C9	C10	C11	0.2(3)
C2	C1	C9	C10	164.8(2)	C1	C9	C14	C13	178.7(2)
C2	C1	C9	C14	-14.2(4)	C10	C9	C14	C13	-0.4(3)
C1	C2	C3	C4	-179.8(2)	C9	C10	C11	C12	0.1(3)
C1	C2	C3	C8	0.8(2)	C10	C11	C12	Cl1	179.61(17)
C15	C2	C3	C4	5.2(4)	C10	C11	C12	C13	-0.1(3)
C15	C2	C3	C8	-174.21(19)	Cl1	C12	C13	C14	-179.83(17)
C1	C2	C15	C16	-81.0(3)	C11	C12	C13	C14	-0.1(3)
C1	C2	C15	C20	98.9(3)	C12	C13	C14	C9	0.3(3)
C3	C2	C15	C16	93.0(3)	C2	C15	C16	C17	178.7(2)
C3	C2	C15	C20	-87.1(3)	C20	C15	C16	C17	-1.2(3)
C2	C3	C4	C5	-179.4(2)	C2	C15	C20	C19	-178.1(2)
C8	C3	C4	C5	0.0(3)	C16	C15	C20	C19	1.7(4)
C2	C3	C8	O1	-1.2(2)	C15	C16	C17	C18	0.1(4)
C2	C3	C8	C7	179.8(2)	C16	C17	C18	C19	0.5(4)
C4	C3	C8	O1	179.21(18)	C17	C18	C19	C20	0.1(4)
C4	C3	C8	C7	0.2(3)	C18	C19	C20	C15	-1.2(4)

Table 6. Anisotropic Displacement Parameters (U_{ij}, Å^2)

Atom	U_{11}	U_{22}	U_{33}	U_{23}	U_{13}	U_{12}
I1	0.05814(13)	0.02749(10)	0.05816(13)	0.00755(7)	-0.02792(10)	-0.00803(8)
Cl1	0.0314(3)	0.0386(3)	0.0386(3)	-0.0007(2)	-0.0057(2)	-0.0119(2)
O1	0.0299(8)	0.0243(7)	0.0321(8)	0.0041(6)	-0.0141(6)	-0.0067(6)
C1	0.0292(11)	0.0202(9)	0.0319(11)	0.0030(8)	-0.0165(9)	-0.0050(8)
C2	0.0271(10)	0.0272(10)	0.0290(11)	0.0035(8)	-0.0140(9)	-0.0075(8)
C3	0.0299(11)	0.0265(10)	0.0280(11)	0.0021(8)	-0.0152(9)	-0.0079(8)
C4	0.0318(11)	0.0357(12)	0.0326(12)	0.0015(9)	-0.0158(10)	-0.0094(9)
C5	0.0303(12)	0.0405(13)	0.0348(13)	-0.0063(10)	-0.0108(10)	-0.0081(10)
C6	0.0310(12)	0.0319(12)	0.0417(13)	-0.0071(10)	-0.0189(10)	-0.0001(9)
C7	0.0363(12)	0.0259(10)	0.0388(13)	-0.0004(9)	-0.0219(10)	-0.0057(9)
C8	0.0292(11)	0.0276(10)	0.0291(11)	0.0009(8)	-0.0146(9)	-0.0074(9)
C9	0.0303(11)	0.0274(10)	0.0277(11)	0.0032(8)	-0.0155(9)	-0.0104(9)
C10	0.0332(11)	0.0249(10)	0.0328(12)	0.0050(8)	-0.0160(10)	-0.0085(9)
C11	0.0363(12)	0.0317(11)	0.0312(12)	0.0086(9)	-0.0135(10)	-0.0157(10)
C12	0.0301(11)	0.0332(11)	0.0274(11)	0.0010(9)	-0.0115(9)	-0.0115(9)
C13	0.0313(11)	0.0266(10)	0.0334(12)	-0.0006(9)	-0.0129(9)	-0.0077(9)
C14	0.0329(11)	0.0274(10)	0.0309(11)	0.0037(9)	-0.0117(9)	-0.0125(9)
C15	0.0245(10)	0.0260(10)	0.0255(10)	0.0042(8)	-0.0083(8)	-0.0067(8)
C16	0.0294(11)	0.0276(11)	0.0359(12)	0.0002(9)	-0.0155(10)	-0.0065(9)
C17	0.0326(12)	0.0352(12)	0.0391(13)	0.0057(10)	-0.0206(10)	-0.0067(10)
C18	0.0336(12)	0.0265(11)	0.0471(14)	0.0082(10)	-0.0157(11)	-0.0072(9)
C19	0.0521(15)	0.0318(12)	0.0565(16)	0.0108(11)	-0.0303(13)	-0.0205(11)
C20	0.0480(14)	0.0354(12)	0.0490(15)	0.0121(11)	-0.0324(12)	-0.0199(11)

The form of the anisotropic displacement parameter is:
$\exp[-2\pi^2(h^2a^{*2}U_{11} + k^2b^{*2}U_{22} + l^2c^{*2}U_{33} + 2klb^*c^*U_{23} + 2hla^*c^*U_{13} + 2hka^*b^*U_{12})]$

Table 7. Derived Atomic Coordinates and Displacement Parameters for Hydrogen Atoms

Atom	*x*	*y*	*z*	U_{eq}, Å^2
H4	0.852628	0.476011	0.016856	0.040
H5	1.075609	0.258576	-0.048673	0.044
H6	1.053036	0.061524	0.078350	0.043
H10	0.253679	0.319351	0.550268	0.036
H11	-0.022786	0.402387	0.724557	0.039
H13	-0.055278	0.807474	0.627120	0.037
H14	0.221265	0.724729	0.454201	0.036
H16	0.361866	0.604755	0.106810	0.037
H17	0.268378	0.829803	0.033845	0.042
H18	0.326260	1.019935	0.096522	0.045
H19	0.480702	0.983730	0.230433	0.051
H20	0.579176	0.758781	0.301169	0.046

1.4.2 X-ray data of 2-(4-Chlorophenyl)-7-iodo-5-methyl-3-phenyl-1-benzofuran (**7g**)

STRUCTURE REPORT

XCL Code: JUS1705 **Date:** 21 June 2017

Compound: 2-(4-Chlorophenyl)-7-iodo-5-methyl-3-phenyl-1-benzofuran
Formula: $C_{21}H_{14}ClIO$

Supervisor: R. M. Al-Zoubi, Jordan University of Science and Technology
Crystallographer: R. McDonald

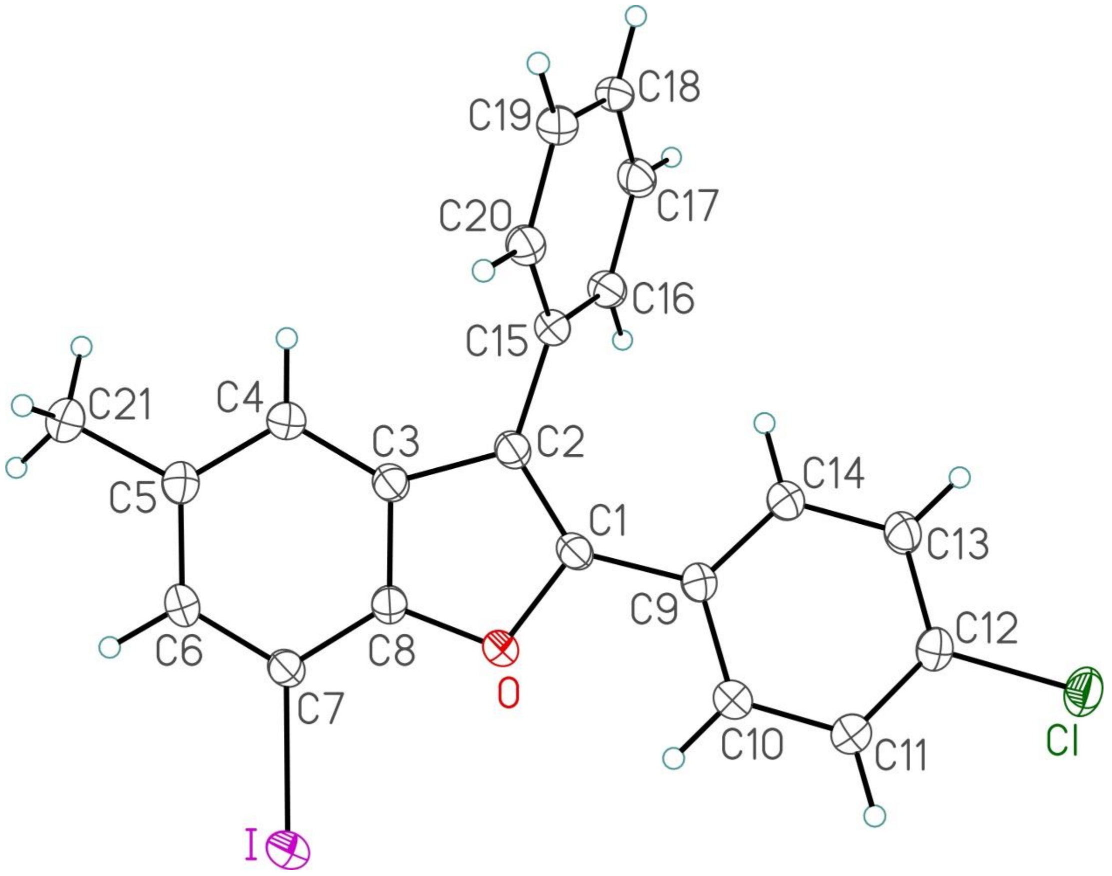

Figure Legends

Figure 1. Perspective view of the 2-(4-chlorophenyl)-7-iodo-5-methyl-3-phenyl-1-benzofuran molecule showing the atom labelling scheme. Non-hydrogen atoms are represented by Gaussian ellipsoids at the 30% probability level. Hydrogen atoms are shown with arbitrarily small thermal parameters.

Figure 2. Alternate view of the molecule.

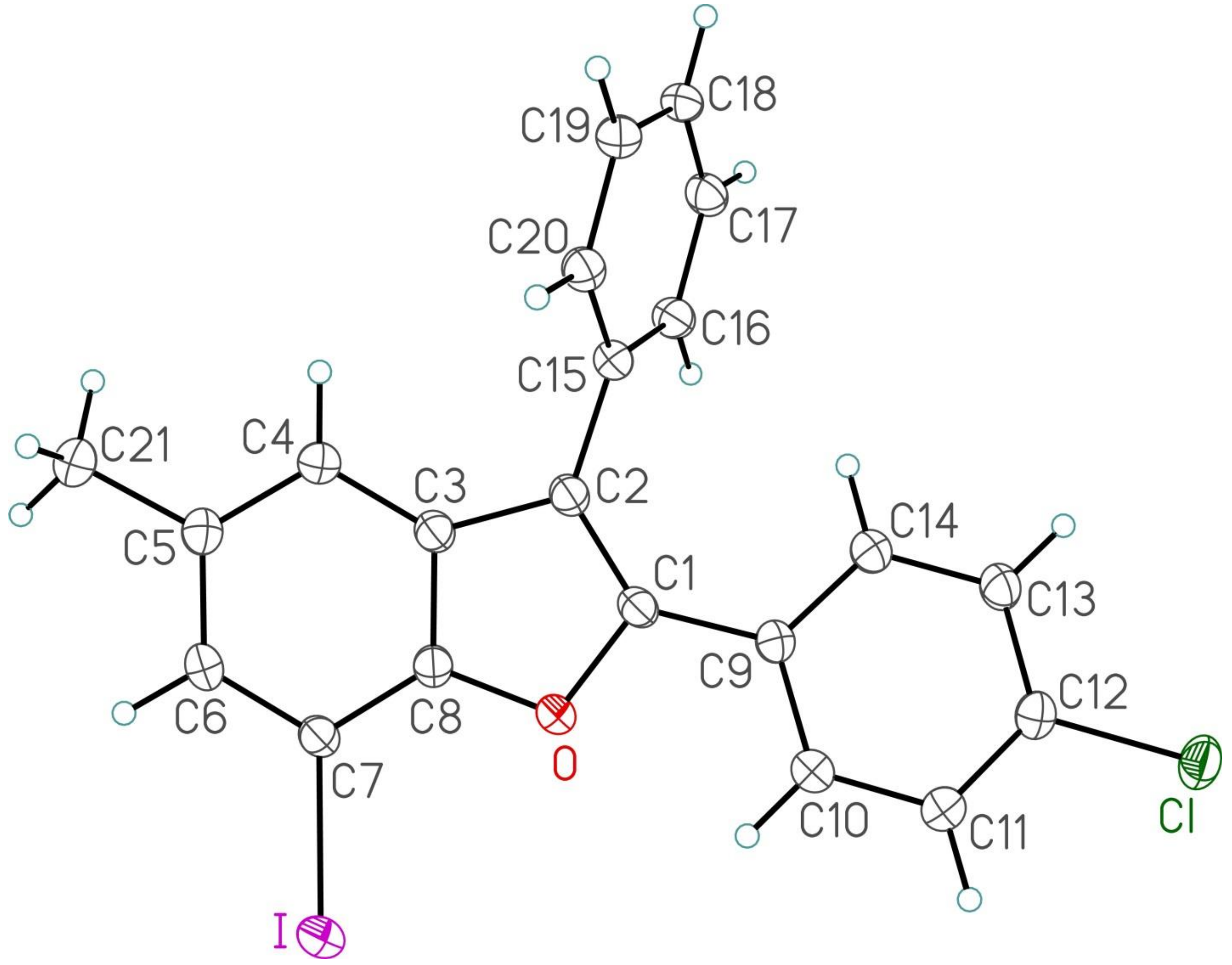
C18
C19
C17
C20
C16
C15
C21
C4
C3
C2
C14
C5
C1
C13
C9
C12
C6
C8
C7
O
C10
C11
Cl
I

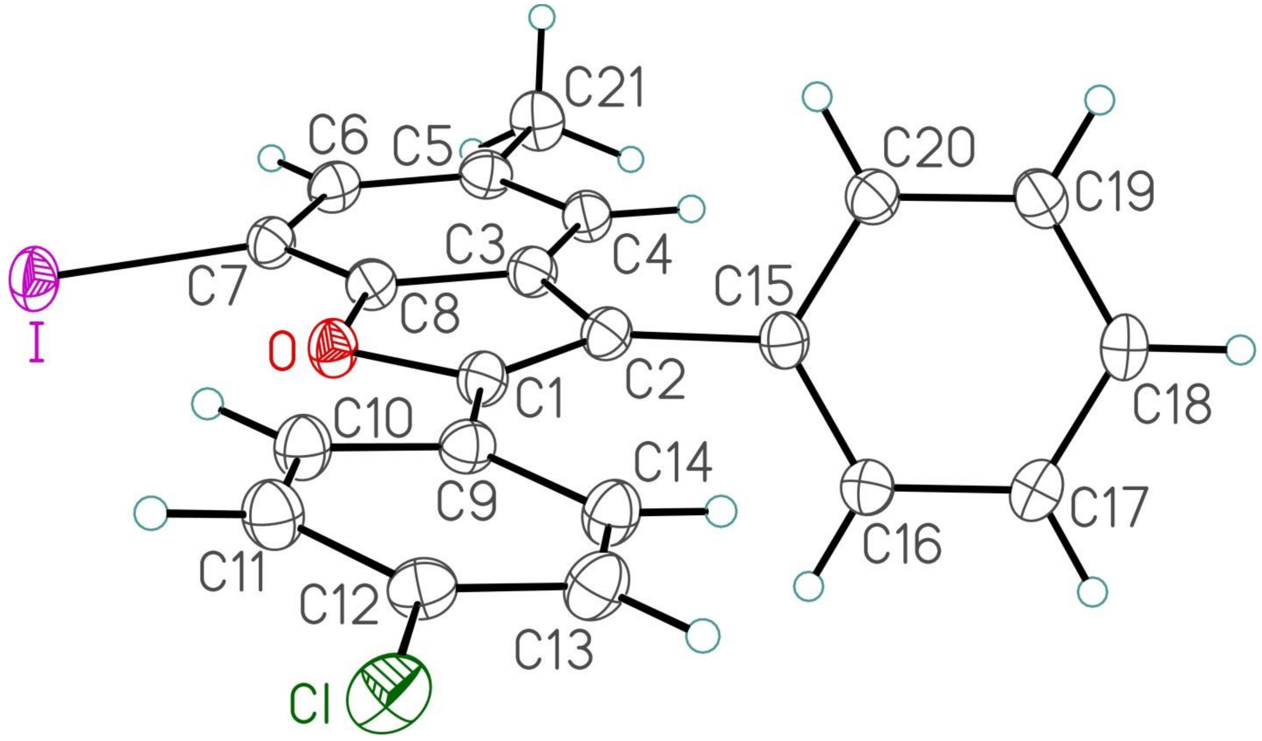
C21
C6
C5
C20
C19
C3
C4
I
C7
C8
C15
O
C2
C18
C1
C10
C14
C9
C17
C16
C11
C12
C13
Cl

List of Tables

Table 1. Crystallographic Experimental Details

Table 2. Atomic Coordinates and Equivalent Isotropic Displacement Parameters

Table 3. Selected Interatomic Distances

Table 4. Selected Interatomic Angles

Table 5. Torsional Angles

Table 6. Anisotropic Displacement Parameters

Table 7. Derived Atomic Coordinates and Displacement Parameters for Hydrogen Atoms

Table 1. Crystallographic Experimental Details

A. Crystal Data	
formula	$C_{21}H_{14}ClIO$
formula weight	444.67
crystal dimensions (mm)	0.34 × 0.13 × 0.11
crystal system	triclinic
space group	$P\bar{1}$ (No. 2)
unit cell parameters[a]	
a (Å)	8.1585 (3)
b (Å)	10.0268 (4)
c (Å)	11.7398 (5)
α (deg)	82.2199 (4)
β (deg)	69.7716 (4)
γ (deg)	70.2331 (4)
V (Å^3)	847.84 (6)
Z	2
ρ_{calcd} (g cm^{-3})	1.742
μ (mm^{-1})	2.050
B. Data Collection and Refinement Conditions	
diffractometer	Bruker PLATFORM/APEX II CCD[b]
radiation (λ [Å])	graphite-monochromated Mo Kα (0.71073)
temperature (°C)	–80
scan type	ω scans (0.3°) (15 s exposures)
data collection 2θ limit (deg)	58.13
total data collected	8279 ($-10 \le h \le 11$, $-13 \le k \le 13$, $-15 \le l \le 15$)
independent reflections	4253 (R_{int} = 0.0088)
number of observed reflections (*NO*)	4008 [$F_o^2 \ge 2\sigma(F_o^2)$]
structure solution method	Patterson/structure expansion (*DIRDIF–2008*[c])
refinement method	full-matrix least-squares on F^2 (*SHELXL–2014*[d])
absorption correction method	Gaussian integration (face-indexed)
range of transmission factors	0.0365–0.0125
data/restraints/parameters	4253 / 0 / 218
goodness-of-fit (S)[e] [all data]	1.053
final R indices[f]	
R_1 [$F_o^2 \ge 2\sigma(F_o^2)$]	0.0338
wR_2 [all data]	0.0944
largest difference peak and hole	3.038 and –0.273 e Å^{-3}

[a]Obtained from least-squares refinement of 9922 reflections with 4.32° < 2θ < 57.58°.

(continued)

Table 1. Crystallographic Experimental Details (continued)

[b]Programs for diffractometer operation, data collection, data reduction and absorption correction were those supplied by Bruker.

[c]Beurskens, P. T.; Beurskens, G.; de Gelder, R.; Smits, J. M. M.; Garcia-Granda, S.; Gould, R. O. (2008). The *DIRDIF-2008* program system. Crystallography Laboratory, Radboud University Nijmegen, The Netherlands.

[d]Sheldrick, G. M. *Acta Crystallogr.* **2015**, *C71*, 3–8.

[e]$S = [\Sigma w(F_o^2 - F_c^2)^2/(n - p)]^{1/2}$ (n = number of data; p = number of parameters varied; $w = [\sigma^2(F_o^2) + (0.0555P)^2 + 0.8241P]^{-1}$ where $P = [\mathrm{Max}(F_o^2, 0) + 2F_c^2]/3$).

[f]$R_1 = \Sigma||F_o| - |F_c||/\Sigma|F_o|$; $wR_2 = [\Sigma w(F_o^2 - F_c^2)^2/\Sigma w(F_o^4)]^{1/2}$.

Table 2. Atomic Coordinates and Equivalent Isotropic Displacement Parameters

Atom	*x*	*y*	*z*	U_{eq}, Å^2
I	-0.16274(3)	1.01251(2)	0.38126(2)	0.04528(9)*
Cl	0.45284(12)	0.31982(8)	0.82656(7)	0.04725(17)*
O	0.0996(3)	0.67582(19)	0.40136(17)	0.0340(4)*
C1	0.1987(3)	0.5312(3)	0.3946(2)	0.0326(5)*
C2	0.2207(3)	0.4777(3)	0.2874(2)	0.0325(5)*
C3	0.1325(3)	0.5952(3)	0.2200(2)	0.0328(5)*
C4	0.1058(4)	0.6091(3)	0.1072(2)	0.0350(5)*
C5	0.0076(4)	0.7423(3)	0.0707(2)	0.0361(5)*
C6	-0.0619(4)	0.8572(3)	0.1483(2)	0.0360(5)*
C7	-0.0389(4)	0.8439(3)	0.2617(2)	0.0339(5)*
C8	0.0604(3)	0.7121(3)	0.2949(2)	0.0321(5)*
C9	0.2623(3)	0.4759(3)	0.4994(2)	0.0317(5)*
C10	0.2462(4)	0.5707(3)	0.5825(3)	0.0372(5)*
C11	0.3065(4)	0.5239(3)	0.6818(3)	0.0394(5)*
C12	0.3806(4)	0.3798(3)	0.7000(2)	0.0359(5)*
C13	0.4003(4)	0.2823(3)	0.6191(3)	0.0398(6)*
C14	0.3412(4)	0.3309(3)	0.5189(3)	0.0385(5)*
C15	0.3167(3)	0.3325(3)	0.2393(2)	0.0325(5)*
C16	0.2530(4)	0.2188(3)	0.2925(3)	0.0367(5)*
C17	0.3407(4)	0.0852(3)	0.2418(3)	0.0390(5)*
C18	0.4917(4)	0.0628(3)	0.1376(3)	0.0371(5)*
C19	0.5566(4)	0.1767(3)	0.0828(2)	0.0387(5)*
C20	0.4691(4)	0.3100(3)	0.1339(2)	0.0361(5)*
C21	-0.0254(5)	0.7632(4)	-0.0501(3)	0.0458(6)*

Anisotropically-refined atoms are marked with an asterisk (*). The form of the anisotropic displacement parameter is: $\exp[-2\pi^2(h^2a^{*2}U_{11} + k^2b^{*2}U_{22} + l^2c^{*2}U_{33} + 2klb^*c^*U_{23} + 2hla^*c^*U_{13} + 2hka^*b^*U_{12})]$.

Table 3. Selected Interatomic Distances (Å)

Atom1	Atom2	Distance	Atom1	Atom2	Distance
I	C7	2.087(3)	C7	C8	1.382(3)
Cl	C12	1.744(3)	C9	C10	1.397(4)
O	C1	1.399(3)	C9	C14	1.399(4)
O	C8	1.368(3)	C10	C11	1.384(4)
C1	C2	1.366(4)	C11	C12	1.383(4)
C1	C9	1.467(3)	C12	C13	1.388(4)
C2	C3	1.450(3)	C13	C14	1.390(4)
C2	C15	1.482(3)	C15	C16	1.394(4)
C3	C4	1.396(4)	C15	C20	1.396(4)
C3	C8	1.396(4)	C16	C17	1.391(4)
C4	C5	1.402(4)	C17	C18	1.382(4)
C5	C6	1.402(4)	C18	C19	1.405(4)
C5	C21	1.508(4)	C19	C20	1.390(4)
C6	C7	1.389(4)			

Table 4. Selected Interatomic Angles (deg)

Atom1	Atom2	Atom3	Angle	Atom1	Atom2	Atom3	Angle
C1	O	C8	106.08(19)	C3	C8	C7	122.1(2)
O	C1	C2	111.0(2)	C1	C9	C10	119.2(2)
O	C1	C9	113.4(2)	C1	C9	C14	122.5(2)
C2	C1	C9	135.5(2)	C10	C9	C14	118.3(2)
C1	C2	C3	106.3(2)	C9	C10	C11	121.4(3)
C1	C2	C15	130.5(2)	C10	C11	C12	119.0(3)
C3	C2	C15	123.1(2)	Cl	C12	C11	119.2(2)
C2	C3	C4	134.2(2)	Cl	C12	C13	119.4(2)
C2	C3	C8	105.6(2)	C11	C12	C13	121.4(2)
C4	C3	C8	120.1(2)	C12	C13	C14	119.0(3)
C3	C4	C5	118.8(2)	C9	C14	C13	120.9(3)
C4	C5	C6	119.3(2)	C2	C15	C16	121.7(2)
C4	C5	C21	121.0(3)	C2	C15	C20	119.3(2)
C6	C5	C21	119.7(2)	C16	C15	C20	118.8(2)
C5	C6	C7	122.3(2)	C15	C16	C17	120.4(3)
I	C7	C6	121.41(19)	C16	C17	C18	120.7(3)
I	C7	C8	121.20(19)	C17	C18	C19	119.3(2)
C6	C7	C8	117.3(2)	C18	C19	C20	119.8(3)
O	C8	C3	110.9(2)	C15	C20	C19	120.8(3)
O	C8	C7	126.9(2)				

Table 5. Torsional Angles (deg)

Atom1	Atom2	Atom3	Atom4	Angle	Atom1	Atom2	Atom3	Atom4	Angle
C8	O	C1	C2	-0.3(3)	C3	C4	C5	C21	-179.9(3)
C8	O	C1	C9	176.5(2)	C4	C5	C6	C7	-0.7(4)
C1	O	C8	C3	-0.3(3)	C21	C5	C6	C7	178.8(3)
C1	O	C8	C7	178.6(3)	C5	C6	C7	I	-174.8(2)
O	C1	C2	C3	0.8(3)	C5	C6	C7	C8	1.6(4)
O	C1	C2	C15	179.2(2)	I	C7	C8	O	-3.7(4)
C9	C1	C2	C3	-175.0(3)	I	C7	C8	C3	175.02(19)
C9	C1	C2	C15	3.4(5)	C6	C7	C8	O	179.9(2)
O	C1	C9	C10	-10.0(3)	C6	C7	C8	C3	-1.4(4)
O	C1	C9	C14	170.9(2)	C1	C9	C10	C11	-179.1(3)
C2	C1	C9	C10	165.7(3)	C14	C9	C10	C11	0.1(4)
C2	C1	C9	C14	-13.4(5)	C1	C9	C14	C13	179.8(3)
C1	C2	C3	C4	-179.0(3)	C10	C9	C14	C13	0.7(4)
C1	C2	C3	C8	-0.9(3)	C9	C10	C11	C12	-1.4(4)
C15	C2	C3	C4	2.4(4)	C10	C11	C12	Cl	-178.8(2)
C15	C2	C3	C8	-179.5(2)	C10	C11	C12	C13	1.9(4)
C1	C2	C15	C16	67.0(4)	Cl	C12	C13	C14	179.5(2)
C1	C2	C15	C20	-116.6(3)	C11	C12	C13	C14	-1.2(4)
C3	C2	C15	C16	-114.8(3)	C12	C13	C14	C9	-0.2(4)
C3	C2	C15	C20	61.5(3)	C2	C15	C16	C17	176.8(2)
C2	C3	C4	C5	178.5(3)	C20	C15	C16	C17	0.4(4)
C8	C3	C4	C5	0.7(4)	C2	C15	C20	C19	-176.6(2)
C2	C3	C8	O	0.8(3)	C16	C15	C20	C19	-0.2(4)
C2	C3	C8	C7	-178.2(2)	C15	C16	C17	C18	-0.3(4)
C4	C3	C8	O	179.2(2)	C16	C17	C18	C19	0.0(4)
C4	C3	C8	C7	0.2(4)	C17	C18	C19	C20	0.2(4)
C3	C4	C5	C6	-0.4(4)	C18	C19	C20	C15	-0.1(4)

Table 6. Anisotropic Displacement Parameters (U_{ij}, Å^2)

Atom	U_{11}	U_{22}	U_{33}	U_{23}	U_{13}	U_{12}
I	0.05562(14)	0.03080(12)	0.04463(13)	-0.00449(8)	-0.01609(9)	-0.00624(9)
Cl	0.0623(4)	0.0477(4)	0.0436(4)	0.0100(3)	-0.0305(3)	-0.0224(3)
O	0.0386(9)	0.0267(8)	0.0332(8)	-0.0012(7)	-0.0133(7)	-0.0038(7)
C1	0.0321(11)	0.0263(11)	0.0353(12)	0.0001(9)	-0.0103(9)	-0.0048(9)
C2	0.0333(11)	0.0286(11)	0.0340(11)	0.0011(9)	-0.0118(9)	-0.0075(9)
C3	0.0321(11)	0.0301(12)	0.0332(11)	0.0006(9)	-0.0099(9)	-0.0072(9)
C4	0.0356(12)	0.0335(12)	0.0341(12)	-0.0019(10)	-0.0105(10)	-0.0089(10)
C5	0.0362(12)	0.0378(13)	0.0340(12)	0.0035(10)	-0.0120(10)	-0.0119(10)
C6	0.0373(12)	0.0314(12)	0.0387(13)	0.0060(10)	-0.0164(10)	-0.0084(10)
C7	0.0355(12)	0.0283(11)	0.0349(12)	0.0001(9)	-0.0111(10)	-0.0068(9)
C8	0.0331(11)	0.0316(12)	0.0306(11)	0.0012(9)	-0.0116(9)	-0.0084(9)
C9	0.0323(11)	0.0310(12)	0.0314(11)	0.0022(9)	-0.0111(9)	-0.0098(9)
C10	0.0394(13)	0.0316(12)	0.0391(13)	-0.0016(10)	-0.0148(11)	-0.0064(10)
C11	0.0428(14)	0.0386(14)	0.0364(13)	-0.0035(10)	-0.0151(11)	-0.0090(11)
C12	0.0375(12)	0.0389(13)	0.0342(12)	0.0051(10)	-0.0149(10)	-0.0146(10)
C13	0.0478(14)	0.0323(13)	0.0425(14)	0.0053(11)	-0.0214(12)	-0.0118(11)
C14	0.0470(14)	0.0310(12)	0.0387(13)	0.0014(10)	-0.0184(11)	-0.0098(11)
C15	0.0333(11)	0.0297(12)	0.0332(11)	-0.0021(9)	-0.0130(9)	-0.0053(9)
C16	0.0367(12)	0.0330(13)	0.0374(12)	-0.0011(10)	-0.0102(10)	-0.0091(10)
C17	0.0446(14)	0.0298(12)	0.0452(14)	0.0004(10)	-0.0188(12)	-0.0109(11)
C18	0.0425(13)	0.0293(12)	0.0400(13)	-0.0072(10)	-0.0184(11)	-0.0039(10)
C19	0.0374(13)	0.0384(14)	0.0338(12)	-0.0034(10)	-0.0089(10)	-0.0052(11)
C20	0.0372(12)	0.0334(13)	0.0347(12)	0.0005(10)	-0.0108(10)	-0.0088(10)
C21	0.0537(17)	0.0459(16)	0.0381(14)	0.0006(12)	-0.0210(12)	-0.0101(13)

The form of the anisotropic displacement parameter is:
$\exp[-2\pi^2(h^2a^{*2}U_{11} + k^2b^{*2}U_{22} + l^2c^{*2}U_{33} + 2klb^*c^*U_{23} + 2hla^*c^*U_{13} + 2hka^*b^*U_{12})]$

Table 7. Derived Atomic Coordinates and Displacement Parameters for Hydrogen Atoms

Atom	*x*	*y*	*z*	U_{eq}, Å^2
H4	0.1533	0.5298	0.0561	0.042
H6	-0.1270	0.9473	0.1226	0.043
H10	0.1927	0.6695	0.5705	0.045
H11	0.2972	0.5897	0.7366	0.047
H13	0.4535	0.1837	0.6319	0.048
H14	0.3544	0.2649	0.4629	0.046
H16	0.1489	0.2327	0.3640	0.044
H17	0.2963	0.0085	0.2791	0.047
H18	0.5510	-0.0287	0.1033	0.045
H19	0.6600	0.1627	0.0109	0.046
H20	0.5136	0.3867	0.0967	0.043
H21A	-0.1452	0.8346	-0.0427	0.055
H21B	-0.0241	0.6734	-0.0750	0.055
H21C	0.0716	0.7953	-0.1111	0.055
